# Supplementary material for: Aortic pressure and forward and backward wave components in children, adolescents and young-adults: Agreement between brachial oscillometry, radial and carotid tonometry data and analysis of factors associated with their differences
Source: PLoS One. 2019 Dec 19;14(12):e0226709. doi: 10.1371/journal.pone.0226709 (PMC6922407; doi:10.1371/journal.pone.0226709)
Supplement: S1 Appendix — A. Wave separation analysis (WSA) [SphygmoCor and Mobil-O-Graph]. B. cSBP, cPP, Pf and Pb absolute and relative intra (repeatability) and inter-observer (reproducibility) variability. (DOCX) [file pone.0226709.s001.docx]

**S1 Appendix (Supplementary Material)**

**Article:** Zinoveev A, Castro J, García-Espinosa V, Marin M, Chiesa P, Bia D, Zócalo Y. Aortic pressure and forward and backward wave components in children, adolescents and young-adults: agreement between brachial oscillometry, radial and carotid tonometry data and analysis of factors associated with their differences. Plos One, 2019.

------------------------------------------------------------------------------------------------------------------------------------------

**A. Wave separation analysis (WSA) [SphygmoCor and Mobil-O-Graph]**

**A.1. SphygmoCor: WSA in records obtained by radial tonometry.**

The wave separation analysis (WSA) quantifies the total amount of arterial wave reflection considering both aortic pulse and flow waves [1]. Using SphygmoCor wave separation software backward (Pb) and forward (Pf) pressure wave components were obtained using the "triangulation method" [1]. This method assumes a triangular shape for the aortic flow velocity wave. Assuming that there is no flow during diastole, the beginning and end of the triangle are lined up with the beginning ("foot" of the aortic pressure wave upstroke during systole) and end (dicrotic notch) of the systole in the aortic pressure wave. The peak (maximum) of the triangle corresponds to the first systolic shoulder (inflection point) of the aortic pressure wave [dashed line, Figure 1]. Pf is the arithmetic mean between measured aortic pressure waveform and the triangle (flow waveform) (white waveform, Figure 1), and Pb is the area between Pf and the aortic pressure waveform (shaded area, Figure 1). Thus, peak Pb would be the maximum distance between Pf and the aortic pressure waveform [1,2].


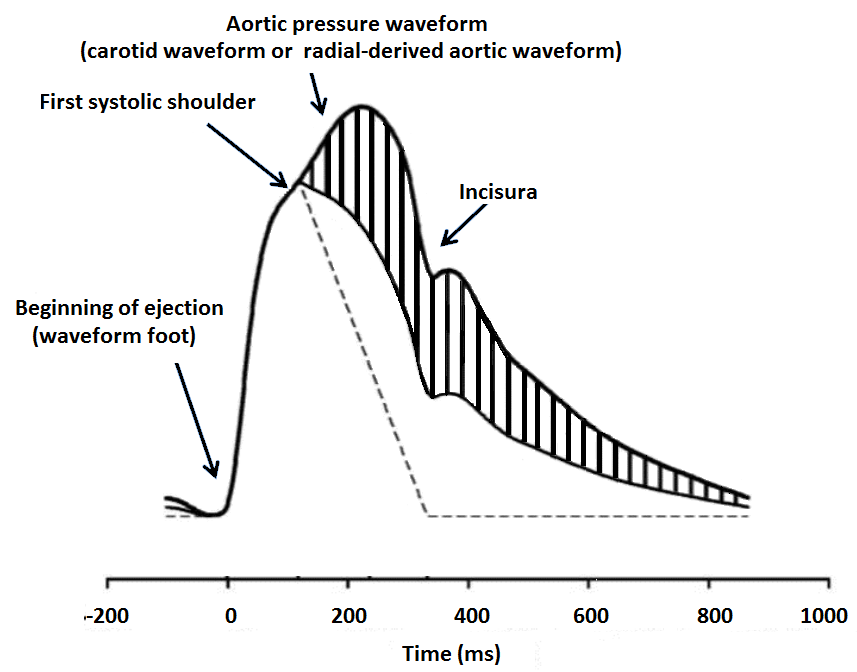


Figure 1. Modified from [2].

After opening the SphygmoCor Software (SCOR; v.9, AtCor-Medical, Australia) in the section “Pulse Wave Analysis” [#1, Figure 2-A], you can obtain radial wave signals by tonometry. Once radial waves were recorded, obtained data can be visualized in the “Report” window [#2, Figure 2-B]. There, you can opt for obtaining “Detailed” data [#3, Figure 2-B]. If so, the (average) radial waveform appears on the left side of the screen and the aortic wave (average; obtained by the application of a generalized transfer function (GTF) on the right. To perform WSA, you must click “Modify” [#4, Figure 2-B]. Then, a new screen appears, to check and complete data, as shown in the figure [#5, Figure 2-C]. At this time you can modify patient data and/or re-calibrate recorded waves (e.g. considering different diastolic blood pressure and mean blood pressure values - as we did in this article -). Thereafter, click “Modify” [#6, Figure 2-C] and you will see a new window named “Wave Separation” [#7, Figure 2-D].

Wave separation or reflection report contains a graphical representation of the aortic pulse including forward and backward pulses and a flow wave, as well as wave reflection parameters. The aortic pressure waveform appears on the left side. This waveform is the sum of two pressure "components or waveforms": the pulse wave generated by cardiac ejection is called Forward Pulse (Pf) and the pulse wave reflected from the arteries back to the heart is called Backward or Reflected Pulse (Pb). The aortic flow waveform is shown in the middle of the screen (top), whereas wave separation analysis data (middle bottom) and aortic forward (blue) and backward (black) components appear on the right side. The reported wave reflection parameters are:

• Forward pulse height or amplitude (Pf; mmHg)

• Backward or reflected pulse height or amplitude (Pb; mmHg)

• Reflection magnitude: Backward pulse Height to forward pulse height ratio, expressed as percentage.


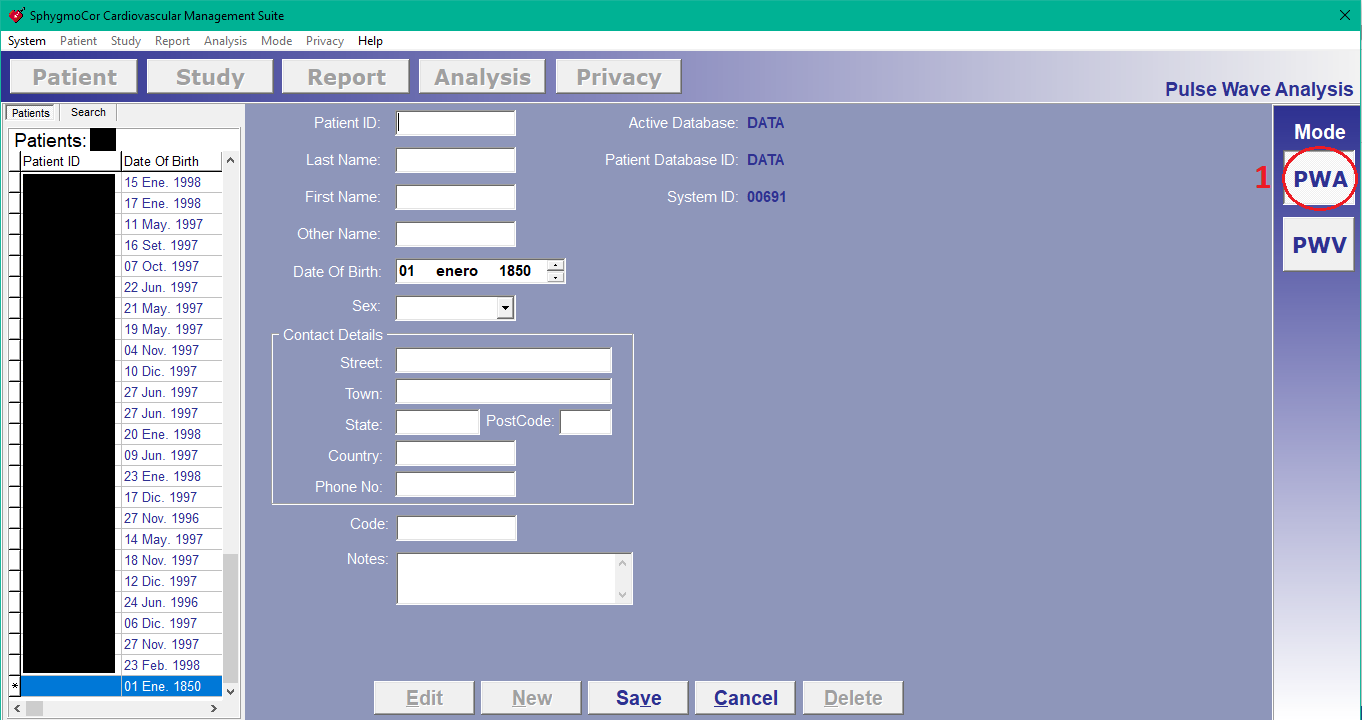


Figure 2-A


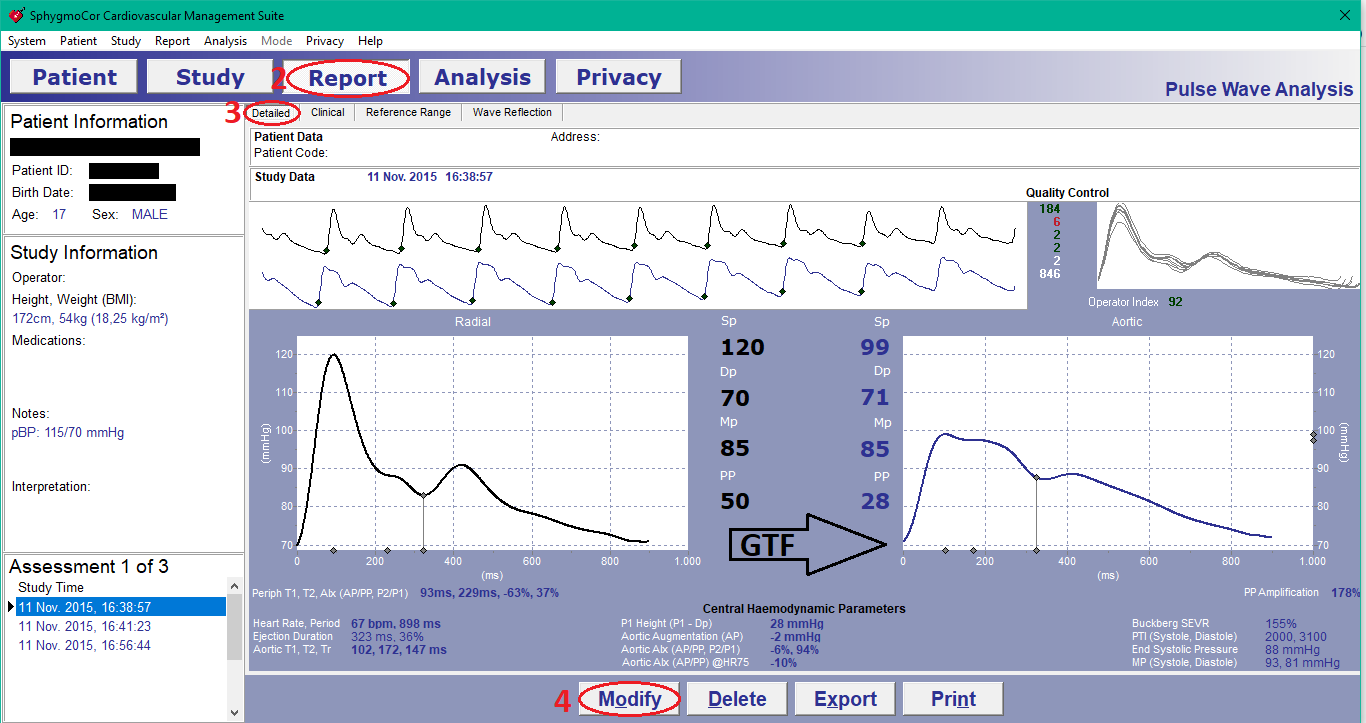


Figure 2-B


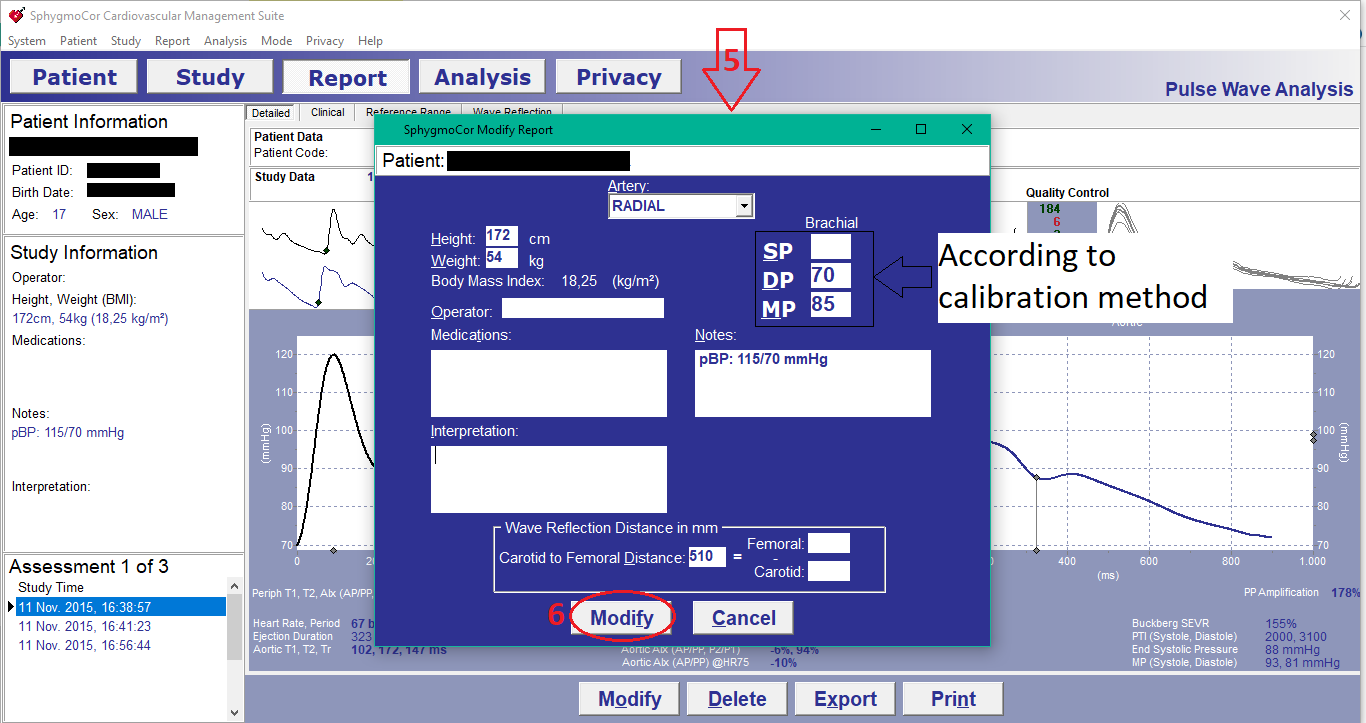


Figure 2-C


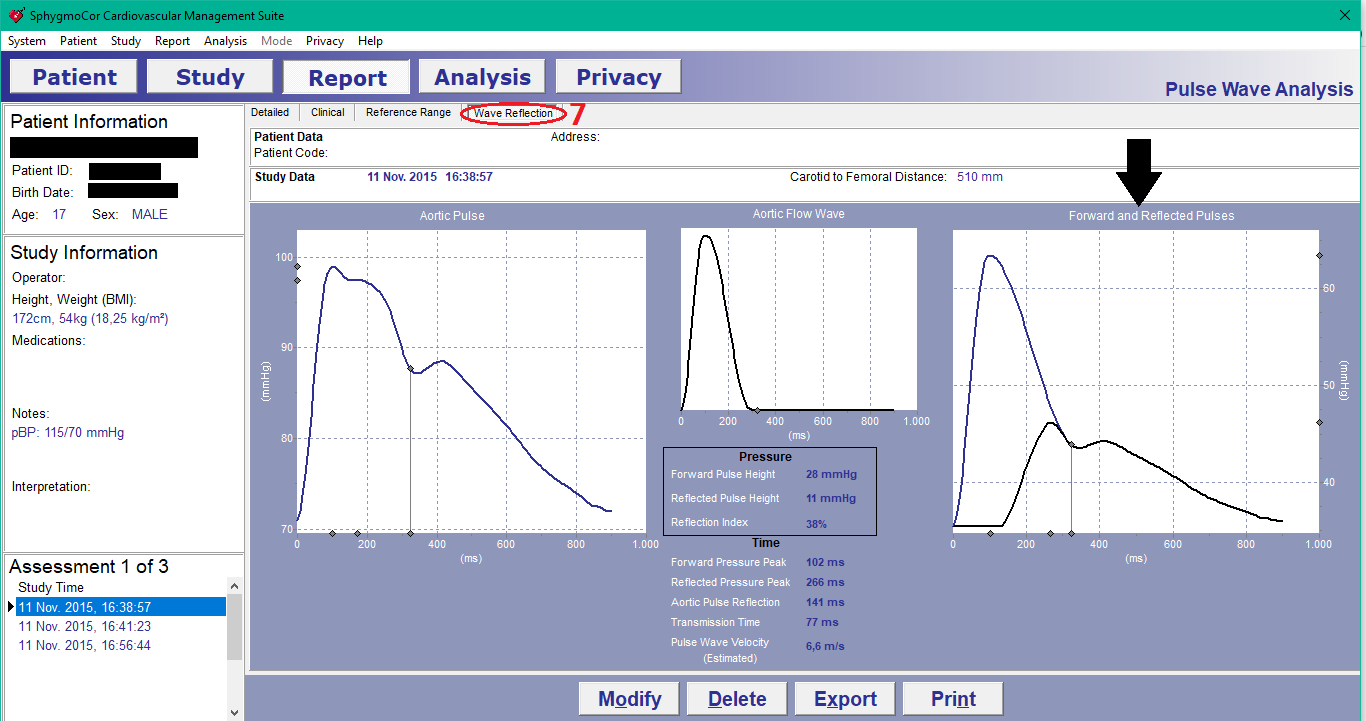


Figure 2-D

**A.2. SphygmoCor: WSA in records obtained from carotid tonometry.**

Similar to what was reported for radial records, it is possible to see "Report" and "Details" of records obtained from the carotid artery [#8 and #9, Figure 2-E]. In that section, as was explained for radial tonometry two registers can be seen, in this case they are identical because the method of estimation of the aortic wave (on the right side) assumes it is identical to the carotid wave (on the left side “Nproc”). To perform WSA, in the bottom of the screen you must click “Modify” [#10, Figure 2-E]. Then, a new screen appears, to check and complete data, as shown on the figure [#11, Figure 2-F]. At this time you can modify patient data and/or re-calibrate the recorded waves (e.g. to different diastolic and mean blood pressure values- as we did in this article -). Then, if you click “Modify” [#12, Figure 2-F] and you will see a new window named “Wave Reflection” [#13, Figure 2-G]. This screen reports the same information previously described (for aortic waves obtained from radial records) [Figure 2-D].


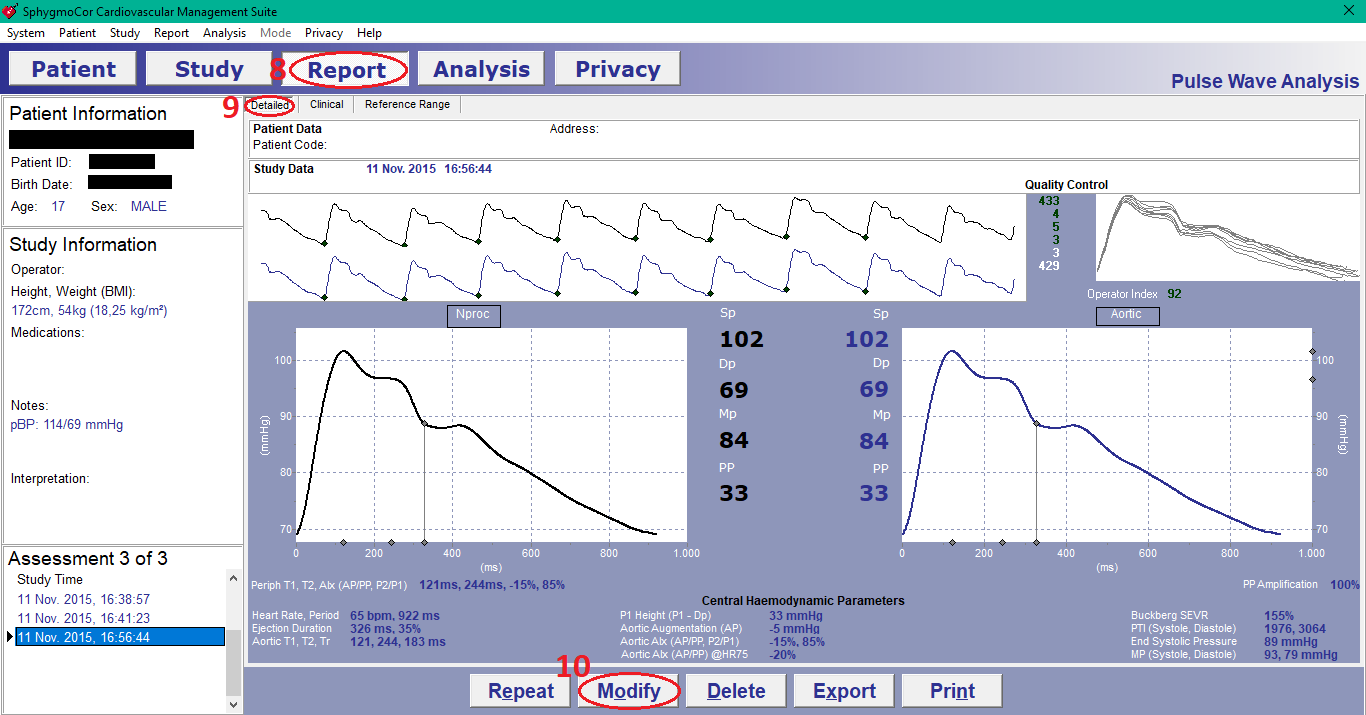


Figure 2-E

**
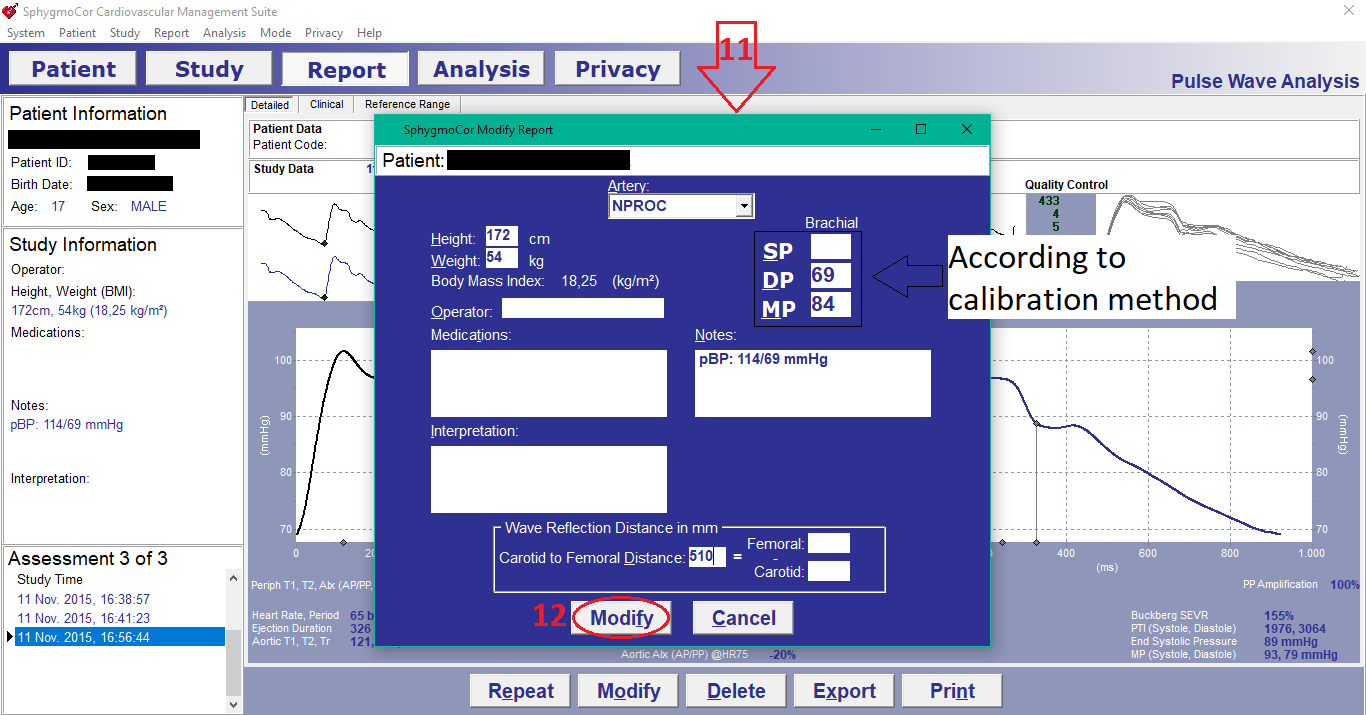
**

Figure 2-F


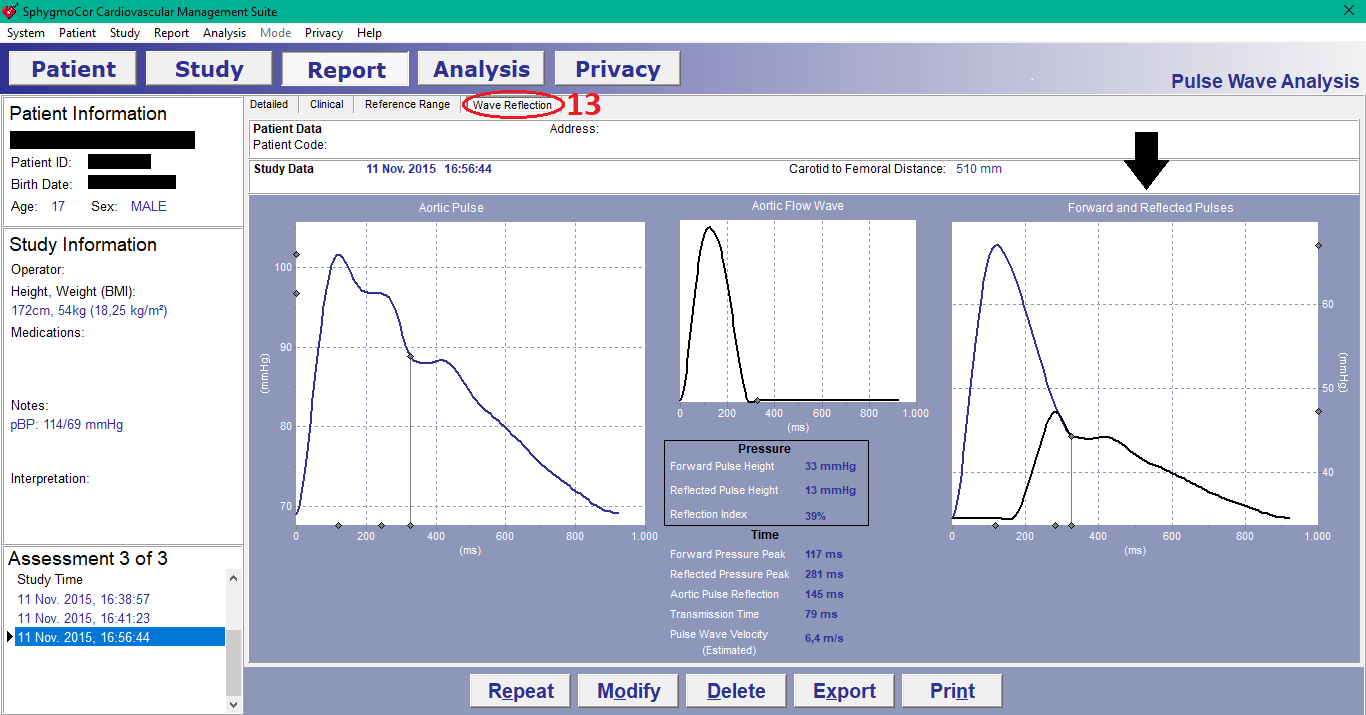


Figure 2-G

**A.3. Mobil-O-Graph: WSA in records obtained from brachial oscillometry.**

The Mobil-O-Graph uses an aortic blood flow model based on higher order Windkessel theory (ARCSolver), to quantify arterial wave reflections parameters [1]. Basically, the method states that the "triangular flow approximation" differs from physiological waves, and consequently applies a different method to estimate flow waves based on pressure curves, and uses the estimated flow waves for WSA. Briefly, aortic pressure curves are derived from the Mobil-O-Graph system. Then, aortic flow curves are estimated from these curves, based on 3 element Windkessel models, where the outflow of the left ventricle is described as a dynamic system of second order. Windkessel equations are formulated as an isoperimetric problem with a constraint to minimize hydraulic work, and mathematical solutions, pressure waveform area fitting, and a second-order linear delay element lead to the final flow shape. Then, WSA is performed in the frequency domain [3].

Unlike the SphygmoCor, the Mobil-O-Graph system (software; screen "Pulse wave analysis") shows the peripheral (brachial) blood pressure (beat-to-beat) waveforms ("Peripheral pulse wave (measured)") and the corresponding "averaged" aortic wave obtained by applying a GTF ("Central pulse wave (calculated)"). At the same time, it shows forward and backward aortic wave components (central part of the screen). Like Sphygmocor software, the wave reflection parameters reported by the Mobil-O-Graph software are:

• Forward pulse height or amplitude (Pf; mmHg)

• Backward or reflected pulse height or amplitude (Pb; mmHg)

• Reflection magnitude: Backward pulse height to forward pulse height ratio, expressed as percentage.


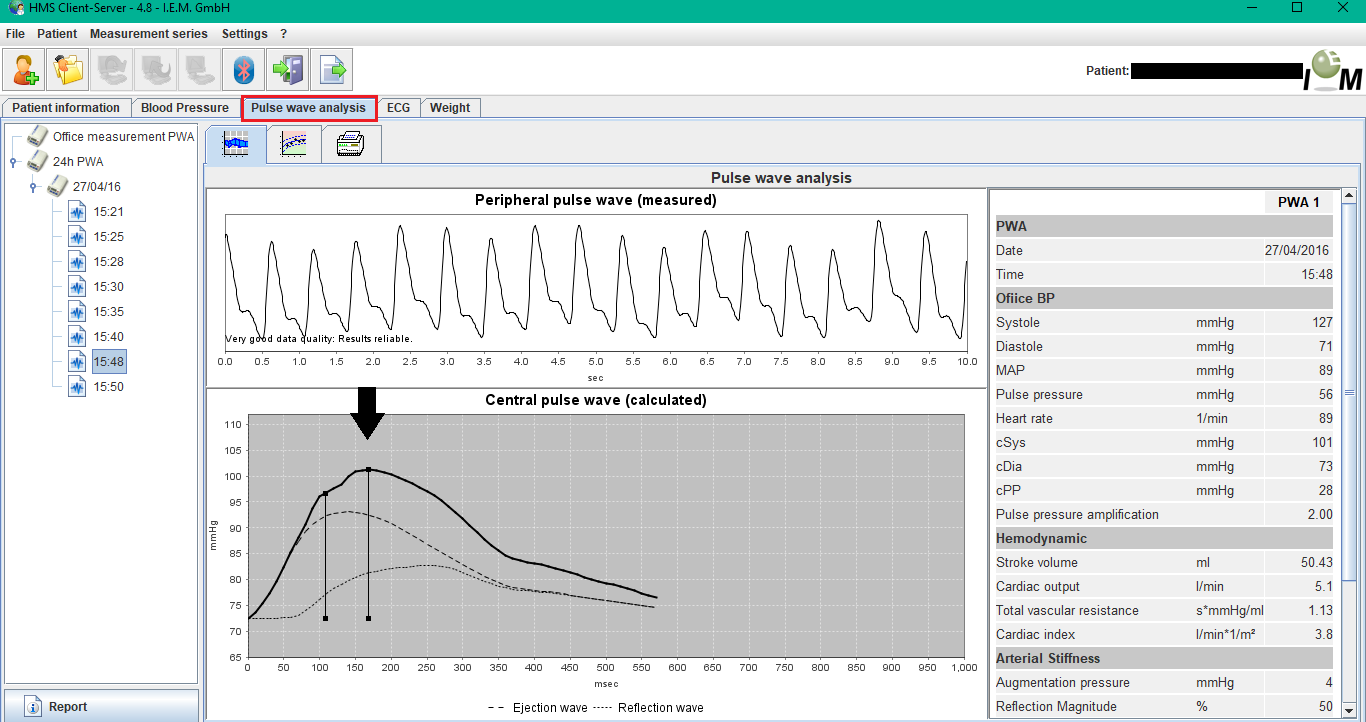


Figure 3.

------------------------------------------------------------------------------------------------------------------------------------------

**B. cSBP, cPP, Pf and Pb absolute and relative intra (repeatability) and inter-observer (reproducibility) variability**

We analyzed absolute and relative intra (repeatability) and inter-observer (reproducibility) variability of cSBP, cPP, Pf and Pb data obtained (total pool of 245 subjects) considering different methodological approaches (RT, CT and BOSC). A single investigator (Y.Z.) performed 2 measurements on the same day on 245 subjects, with an intra-observer difference of 0.1±4.3 mmHg (RT), 0.2±4.1 mmHg (CT) and -0.1±3.8 mmHg (BOSC) for cSBP; 0.7±3.4 mmHg (RT), 0.5±4.0 mmHg (CT) and 0.6±3.4 mmHg (BOSC) for cPP; 0.1±2.7 mmHg (RT), 0.1±2.6 mmHg (CT) and -0.2±2.8 mm Hg (BOSC) for Pf; 0.1±2.7 mmHg (RT), 0.1±2.6 mmHg (CT) and 0.1±2.3 mmHg (BOSC) for cPb. For the inter-observer studies, two investigators (Y.Z., D.B) performed two measurements on two separate occasions, resulting in an inter-observer difference of 0.1±4.0 mmHg (RT), 0.1±3.9 mmHg (CT) and 0.1±4.0 mmHg (BOSC) for cSBP; 0.6±3.2 mmHg (RT), 0.4±4.1 mmHg (CT) and 0.5±3.0 mmHg (BOSC) for cPP; -0.1±2.9 mmHg (RT), 0.1±2.8 mmHg (CT) and 0.2±2.9 mmHg (BOSC) for Pf; -0.1±2.6 mmHg (RT), -0.1±2.5 mmHg (CT) and 0.1±2.2 mmHg (BOSC) for cPb. There were no significant differences in cSBP, cPP, Pf or Pb within visits, between the two records or between the researchers, indicating excellent repeatability and intra- and inter-observer reproducibility, as previously suggested [4,5,6,7]. In all cases, the relative inter- and intraobserver variability was ˂6%.

**Supplementary References**

1. Hametner B, Wassertheurer S, Kropf J, Mayer C, Holzinger A, Eber B, et al. Wave reflection quantification based on pressure waveforms alone--methods, comparison, and clinical covariates. Comput Methods Programs Biomed. 2013;109(3):250-259.
2. Imraan Ballim. Aortic backward waves derived from wave separation analysis, and end-organ changes. Master of Science Thesis. Faculty of Health Sciences, University of the Witwatersrand, Johannesburg; Johannesburg, 2016. <https://pdfs.semanticscholar.org/69b2/a75e16e35044bf839b881860030643c5f10d.pdf>
3. Weber T, Wassertheurer S, Rammer M, Haiden A, Hametner B, Eber B. Wave reflections, assessed with a novel method for pulse wave separation, are associated with end-organ damage and clinical outcomes. Hypertension. 2012;60(2):534-541.
4. Frimodt-Møller M, Nielsen AH, Kamper AL, Strandgaard S. Reproducibility of pulse-wave analysis and pulse-wave velocity determination in chronic kidney disease. Nephrol Dial Transplant. 2008;23(2):594-600.
5. Savage MT, Ferro CJ, Pinder SJ, Tomson CR. Reproducibility of derived central arterial waveforms in patients with chronic renal failure. Clin Sci (Lond). 2002;103(1):59-65.
6. Laugesen E, Rossen NB, Høyem P, Christiansen JS, Knudsen ST, Hansen KW, et al. Reproducibility of pulse wave analysis and pulse wave velocity in patients with type 2 diabetes. Scand J Clin Lab Invest. 2013;73(5):428-35.
7. Adji A, Hirata K, O'Rourke MF. Clinical use of indices determined non-invasively from the radial and carotid pressure waveforms. Blood Press Monit. 2006 Aug;11(4):215-21.
