# Supplementary material for: Aortic pressure and forward and backward wave components in children, adolescents and young-adults: Agreement between brachial oscillometry, radial and carotid tonometry data and analysis of factors associated with their differences
Source: PLoS One. 2019 Dec 19;14(12):e0226709. doi: 10.1371/journal.pone.0226709 (PMC6922407; doi:10.1371/journal.pone.0226709)
Supplement: S1 Fig — (DOCX) [file pone.0226709.s002.docx]

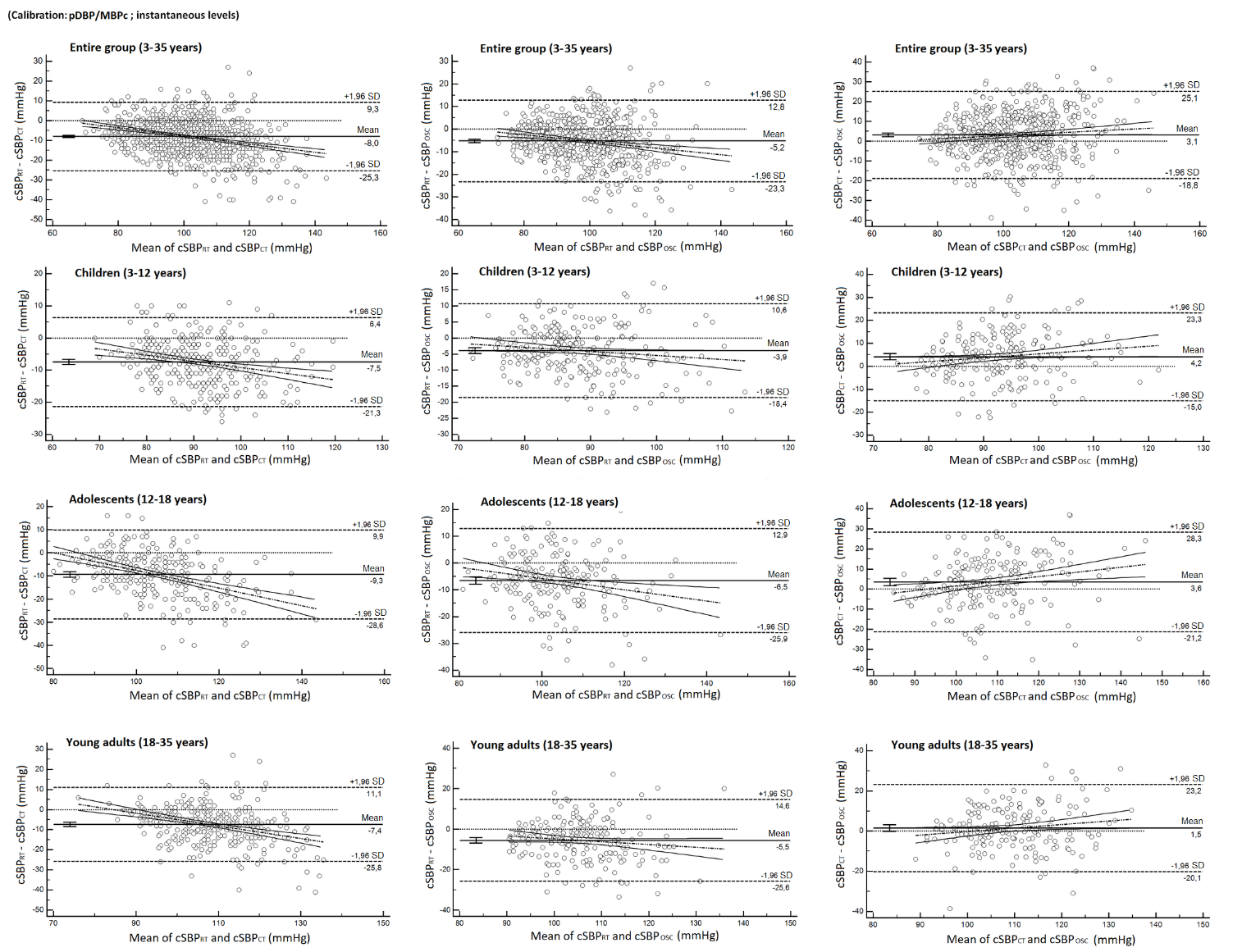


**S1 Fig. Bland-Altman graphs for cSBP (Calibration: pDBP/MBPc; instantaneous levels): entire and age-related groups.** cSBP: central (aortic) systolic blood pressure. pSBP and pDBP: peripheral (brachial) systolic and diastolic blood pressure.MBPc: mean blood pressure calculated as pDBP+((pSBP-pDBP)/3). MBPosc: mean blood pressure measured by oscillometry.RT: radial applanation tonometry record, obtained with SphygmoCor device (SCOR). CT: carotid applanation tonometry record, obtained with SCOR. BOSC: brachial oscillometry/plethysmography record, obtained with Mobil-O-Graph device (MOG).
