# Supplementary material for: Aortic pressure and forward and backward wave components in children, adolescents and young-adults: Agreement between brachial oscillometry, radial and carotid tonometry data and analysis of factors associated with their differences
Source: PLoS One. 2019 Dec 19;14(12):e0226709. doi: 10.1371/journal.pone.0226709 (PMC6922407; doi:10.1371/journal.pone.0226709)
Supplement: S16 Fig — (DOCX) [file pone.0226709.s017.docx]

**

S16 Fig. Bland-Altman-derived Pb Mean Error levels obtained with three different recording methods and calibration schemes: pDBP/MBPc (instantaneous levels), pDBP/ MBPc (equal levels) and pDBP/MBPosc (equal levels).** Analysis for the entire group and children, adolescents and young adults subgroups.cSBP: central (aortic) systolic blood pressure. pSBP and pDBP: peripheral (brachial) systolic and diastolic blood pressure.MBPc: mean blood pressure calculated as pDBP+((pSBP-pDBP)/3). MBPosc: mean blood pressure measured by oscillometry.RT: radial applanation tonometry record, obtained with SphygmoCor device (SCOR). CT: carotid applanation tonometry record, obtained with SCOR. BOSC: brachial oscillometry/plethysmography record, obtained with Mobil-O-Graph device (MOG).
