# Supplementary material for: Aortic pressure and forward and backward wave components in children, adolescents and young-adults: Agreement between brachial oscillometry, radial and carotid tonometry data and analysis of factors associated with their differences
Source: PLoS One. 2019 Dec 19;14(12):e0226709. doi: 10.1371/journal.pone.0226709 (PMC6922407; doi:10.1371/journal.pone.0226709)
Supplement: S17 Fig — (DOCX) [file pone.0226709.s018.docx]

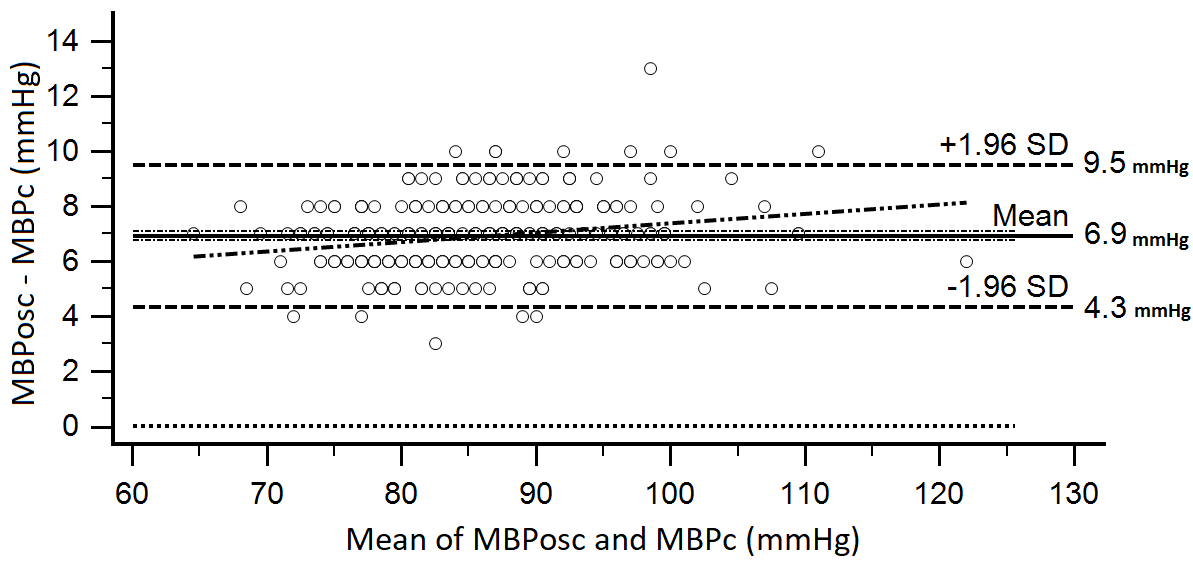


**S17 Fig. Bland-Altman-derived mean and systematic error levels for MBPosc and MBPc differences.** MBPc: mean blood pressure calculated as pDBP+((pSBP-pDBP)/3). MBPosc: mean blood pressure measured by oscillometry.
