# Supplementary material for: Aortic pressure and forward and backward wave components in children, adolescents and young-adults: Agreement between brachial oscillometry, radial and carotid tonometry data and analysis of factors associated with their differences
Source: PLoS One. 2019 Dec 19;14(12):e0226709. doi: 10.1371/journal.pone.0226709 (PMC6922407; doi:10.1371/journal.pone.0226709)
Supplement: S1 Table — (DOCX) [file pone.0226709.s019.docx]

| **S1 Table. Clinical features and cardiovascular risk factors for the entire and age-related subgroups** | | | | | | | | | | | | | | | | | | | | | | | | |
| --- | --- | --- | --- | --- | --- | --- | --- | --- | --- | --- | --- | --- | --- | --- | --- | --- | --- | --- | --- | --- | --- | --- | --- | --- |
|  |  |  |  |  |  |  |  |  |  |  |  |  |  |  |  |  |  |  |  |  |  |  |  |  |
|  | **Entire group [n = 1685]** | | | | | | **Children [3 - 12 years; n = 728]** | | | | | | **Adolescents [12 - 18 years; n = 361]** | | | | | | **Young adults [18 - 35 years; n = 596]** | | | | | |
|  | **MV** | **SD** | **Min** | **p25th** | **p75th** | **Max** | **MV** | **SD** | **Min** | **p25th** | **p75th** | **Max** | **MV** | **SD** | **Min** | **p25th** | **p75th** | **Max** | **MV** | **SD** | **Min** | **p25th** | **p75th** | **Max** |
|  |  |  |  |  |  |  |  |  |  |  |  |  |  |  |  |  |  |  |  |  |  |  |  |  |
| Age (years) | 14.4 | 7.5 | 2.8 | 6.5 | 19.7 | 34.9 | 7.2 | 1.9 | 2.8 | 5.9 | 8.3 | 12.0 | 15.4 | 2.1 | 12.0 | 13.6 | 17.7 | 18.0 | 22.6 | 4.7 | 18.0 | 19.1 | 24.2 | 34.9 |
| Sex female, n (%) |  | 854 |  | [50.7] |  |  |  | 332 |  | [45.7] |  |  |  | 175 |  | [49.0] |  |  |  | 347 |  | [58.1] |  |  |
| Body height (m) | 1.47 | 0.24 | 0.9 | 1.20 | 1.67 | 1.95 | 1.23 | 0.13 | 0.9 | 1.13 | 1.31 | 1.68 | 1.63 | 0.10 | 1.35 | 1.56 | 1.70 | 1.88 | 1.68 | 0.09 | 1.45 | 1.61 | 1.74 | 1.95 |
| Body weight (kg) | 49.0 | 22.6 | 12.3 | 25.7 | 64.0 | 130.0 | 29.2 | 12.9 | 12.3 | 20.5 | 34.0 | 91.1 | 61.8 | 16.5 | 30.0 | 50.7 | 69.3 | 130.0 | 66.2 | 14.1 | 38.1 | 55.6 | 74.2 | 127.0 |
| BMI (m/kg^2^) | 21.3 | 5.0 | 11.5 | 17.3 | 23.8 | 46.1 | 18.7 | 4.2 | 11.5 | 15.7 | 20.5 | 38.9 | 23.1 | 5.2 | 14.6 | 19.7 | 25.0 | 45.5 | 23.4 | 4.0 | 16.5 | 20.6 | 25.3 | 46.1 |
| z-BMI* (kg/m^2^) | 1.30 | 1.88 | -3.81 | 0.07 | 2.19 | 9.64 | 1.36 | 1.88 | -3.81 | 0.10 | 2.35 | 9.64 | 1.07 | 1.88 | -2.07 | -0.10 | 1.91 | 8.13 | ־ | ־ | ־ | ־ | ־ | ־ |
| Hypertension and/or HBP, n [%] |  | 206 |  | [12.2] |  |  |  | 85 |  | [11.7] |  |  |  | 54 |  | [15] |  |  |  | 67 |  | [11.3] |  |  |
| Diabetes, n [%] |  | 7 |  | [0.4] |  |  |  | 4 |  | [0.6] |  |  |  | 3 |  | [0.8] |  |  |  | 0 |  | [0] |  |  |
| Dyslipidemia, n [%] |  | 113 |  | [6.7] |  |  |  | 35 |  | [4.8] |  |  |  | 23 |  | [6.4] |  |  |  | 55 |  | [9.2] |  |  |
| Obesity, n [%] |  | 304 |  | [18.7] |  |  |  | 204 |  | [28.5] |  |  |  | 60 |  | [17.6] |  |  |  | 40 |  | [7] |  |  |
| Smoking, n [%] |  | 145 |  | [8.8] |  |  |  | 0 |  | [0] |  |  |  | 14 |  | [3.9] |  |  |  | 131 |  | [23.3] |  |  |
| Family history of CV disease [%] |  | 0 |  | [0] |  |  |  | 0 |  | [0] |  |  |  | 0 |  | [0] |  |  |  | 0 |  | [0] |  |  |
| Sedentary lifestyle, n [%] |  | 512 |  | [36.7] |  |  |  | 159 |  | [22.4] |  |  |  | 146 |  | [47.9] |  |  |  | 207 |  | [54.3] |  |  |
| MV: mean value. SD: standard deviation. Min: minimum value. max. Maximum value. p25th and p75th: percentile 25 and 75, respectively. BMI: body mass index. zBMI: z score of BMI *calculated only for under 18 years old. HBP: high blood pressure state during cardiovascular evaluation. CV: cardiovascular. | | | | | | | | | | | | | | | | | | | | | | | | |
|  |  |  |  |  |  |  |  |  |  |  |  |  |  |  |  |  |  |  |  |  |  |  |  |  |
