# Supplementary material for: Aortic pressure and forward and backward wave components in children, adolescents and young-adults: Agreement between brachial oscillometry, radial and carotid tonometry data and analysis of factors associated with their differences
Source: PLoS One. 2019 Dec 19;14(12):e0226709. doi: 10.1371/journal.pone.0226709 (PMC6922407; doi:10.1371/journal.pone.0226709)
Supplement: S2 Table — (DOCX) [file pone.0226709.s020.docx]

| **S2 Table. Haemodynamic and aortic wave-derived parameters measured with three different methods in the entire and age-related groups** | | | | | | | | | | | | | | | |
| --- | --- | --- | --- | --- | --- | --- | --- | --- | --- | --- | --- | --- | --- | --- | --- |
|  |  |  |  |  |  |  |  |  |  |  |  |  |  |  |  |
|  |  |  |  |  |  |  |  |  |  |  |  |  |  |  |  |
| **Entire group [3 - 35 years; n = 1685]** | | | | | | | | | | | | | | | |
|  | **RT (Scor)** | | | | **CT (Scor)** | | | | **BOSC (MOG)** | | | | **P value** | | |
|  | **MV** | **SD** | **Min.** | **Max.** | **MV** | **SD** | **Min.** | **Max.** | **MV** | **SD** | **Min.** | **Max.** | **RT vs CT** | **RT vs OSC** | **CT vs OSC** |
| pSBP (mmHg) | 115 | 13 | 70 | 177 | 114 | 14 | 80 | 217 | 113 | 12 | 81 | 154 | 1.000 | **0.042** | 0.205 |
| pDBP (mmHg) | 64 | 9 | 42 | 100 | 63 | 8 | 38 | 100 | 64 | 8 | 39 | 106 | **0.001** | 0.877 | 0.056 |
| MBPc (Form factor: 33%) (mmHg) | 81 | 9 | 43 | 120 | 80 | 9 | 56 | 115 | 80 | 9 | 55 | 120 | 0.080 | 0.082 | 1.000 |
| HR (beats/minute) | 76 | 14 | 42 | 151 | 75 | 14 | 43 | 145 | 79 | 15 | 44 | 134 | 1.000 | **<0.001** | **<0.001** |
| cSBP (mmHg) | 99 | 12 | 67 | 156 | 106 | 15 | 69 | 216 | 100 | 14 | 71 | 169 | **<0.001** | 0.185 | **<0.001** |
| cPP (mmHg) | 33 | 10 | 8 | 81 | 43 | 14 | 10 | 152 | 35 | 12 | 15 | 88 | **<0.001** | **0.039** | **<0.001** |
| Pf (mmHg) | 31 | 10 | 7 | 82 | 43 | 14 | 8 | 114 | 24 | 8 | 10 | 66 | **<0.001** | **<0.001** | **<0.001** |
| Pb (mmHg) | 13 | 4 | 4 | 78 | 15 | 5 | 6 | 33 | 13 | 5 | 4 | 38 | **<0.001** | 1.000 | **<0.001** |
| **Children [3 - 12 years; n = 728]** | | | | | | | | | | | | | | | |
| pSBP (mmHg) | 105 | 10 | 72 | 140 | 105 | 12 | 80 | 188 | 107 | 1 | 81 | 140 | 1.000 | **0.012** | **0.011** |
| pDBP (mmHg) | 60 | 7 | 42 | 88 | 60 | 7 | 43 | 86 | 60 | 6 | 39 | 85 | 1.000 | 1.000 | 0.324 |
| MBPc (Form factor: 33%) (mmHg) | 75 | 7 | 58 | 100 | 75 | 7 | 60 | 100 | 76 | 1 | 55 | 101 | 1.000 | 0.105 | 0.122 |
| HR (beats/minute) | 85 | 14 | 51 | 151 | 85 | 1 | 54 | 145 | 87 | 14 | 52 | 134 | 1.000 | 0.247 | 0.107 |
| cSBP (mmHg) | 87 | 9 | 67 | 119 | 95 | 11 | 69 | 152 | 90 | 9 | 71 | 128 | **<0.001** | **<0.001** | **<0.001** |
| cPP (mmHg) | 24 | 7 | 8 | 49 | 36 | 10 | 16 | 102 | 28 | 7 | 15 | 61 | **<0.001** | **0.003** | **<0.001** |
| Pf (mmHg) | 24 | 8 | 7 | 48 | 36 | 10 | 17 | 76 | 20 | 5 | 10 | 51 | **<0.001** | **<0.001** | **<0.001** |
| Pb (mmHg) | 11 | 5 | 4 | 78 | 13 | 4 | 6 | 27 | 11 | 3 | 5 | 25 | **<0.001** | **0.004** | **<0.001** |
| **Adolescents [12 - 18 years; n = 361]** | | | | | | | | | | | | | | | |
| pSBP (mmHg) | 117 | 11 | 90 | 161 | 117 | 12 | 88 | 160 | 119 | 11 | 96 | 154 | 1.000 | 0.271 | 0.717 |
| pDBP (mmHg) | 63 | 8 | 43 | 90 | 62 | 8 | 40 | 89 | 66 | 7 | 47 | 87 | 0.599 | **<0.001** | **<0.001** |
| MBPc (Form factor: 33%) (mmHg) | 81 | 8 | 43 | 108 | 81 | 8 | 56 | 105 | 83 | 8 | 63 | 107 | 1.000 | **0.005** | **0.003** |
| HR (beats/minute) | 74 | 14 | 42 | 120 | 73 | 13 | 43 | 126 | 73 | 13 | 45 | 124 | 0.700 | 1.000 | 1.000 |
| cSBP (mmHg) | 101 | 10 | 76 | 133 | 110 | 14 | 84 | 158 | 107 | 12 | 84 | 157 | **<0.001** | **<0.001** | 0.090 |
| cPP (mmHg) | 36 | 9 | 11 | 70 | 47 | 13 | 12 | 90 | 40 | 12 | 18 | 86 | **<0.001** | **<0.001** | **<0.001** |
| Pf (mmHg) | 34 | 9 | 10 | 68 | 47 | 15 | 12 | 114 | 27 | 8 | 12 | 60 | **<0.001** | **<0.001** | **<0.001** |
| Pb (mmHg) | 13 | 3 | 4 | 25 | 16 | 5 | 6 | 33 | 15 | 5 | 4 | 36 | **<0.001** | **<0.001** | 0.503 |
| **Young adults [18 - 35 years; n = 596]** | | | | | | | | | | | | | | | |
| pSBP (mmHg) | 120 | 12 | 70 | 177 | 121 | 13 | 80 | 217 | 120 | 11 | 95 | 154 | 1.000 | 1.000 | 1.000 |
| pDBP (mmHg) | 68 | 9 | 44 | 100 | 66 | 8 | 38 | 100 | 68 | 8 | 41 | 106 | **0.013** | 0.617 | **0.003** |
| MBPc (Form factor: 33%) (mmHg) | 85 | 9 | 57 | 120 | 84 | 8 | 60 | 115 | 85 | 8 | 61 | 120 | 0.292 | 1.000 | 0.273 |
| HR (beats/minute) | 71 | 12 | 43 | 113 | 70 | 12 | 43 | 104 | 69 | 11 | 44 | 101 | 0.132 | 0.079 | 1.000 |
| cSBP (mmHg) | 104 | 10 | 79 | 156 | 112 | 14 | 73 | 216 | 110 | 13 | 84 | 169 | **<0.001** | **<0.001** | 0.315 |
| cPP (mmHg) | 36 | 10 | 13 | 81 | 46 | 14 | 10 | 152 | 41 | 12 | 21 | 88 | **<0.001** | **<0.001** | **<0.001** |
| Pf (mmHg) | 34 | 10 | 9 | 82 | 46 | 14 | 8 | 98 | 27 | 8 | 14 | 66 | **<0.001** | **<0.001** | **<0.001** |
| Pb (mmHg) | 14 | 4 | 6 | 39 | 17 | 5 | 6 | 31 | 16 | 6 | 7 | 39 | **<0.001** | **<0.001** | 0.060 |
| MV: mean value. SD: standard deviation. RT: radial applanation tonometry record, obtained with SphygmoCor device. CT: carotid applanation tonometry record, obtained with SphygmoCor device. BOSC: brachial oscillometry/plethysmography record, obtained with Mobil-O-Graph device. Min. and Max.: minimum and maximum value, respectively. pSBP, pDBP, MBPc: peripheral (brachial) systolic, diastolic and mean (calculated) blood pressure, respectively. HR: heart rate. cSBP, cPP: central systolic and pulse blood pressure, respectively. Pf: forward wave height (amplitude). Pb: backward wave height (amplitude). p value was obtained from ANOVA plus Bonferroni post-hoc test. Significance level: p<0.05 (red text). | | | | | | | | | | | | | | | |
|  |  |  |  |  |  |  |  |  |  |  |  |  |  |  |  |
|  |  |  |  |  |  |  |  |  |  |  |  |  |  |  |  |
|  |  |  |  |  |  |  |  |  |  |  |  |  |  |  |  |
|  |  |  |  |  |  |  |  |  |  |  |  |  |  |  |  |
