# Supplementary material for: Aortic pressure and forward and backward wave components in children, adolescents and young-adults: Agreement between brachial oscillometry, radial and carotid tonometry data and analysis of factors associated with their differences
Source: PLoS One. 2019 Dec 19;14(12):e0226709. doi: 10.1371/journal.pone.0226709 (PMC6922407; doi:10.1371/journal.pone.0226709)
Supplement: S3 Table — (DOCX) [file pone.0226709.s021.docx]

| **S3 Table. cSBP, cPP, Pf and Pb: correlation and agreement among values obtained with three different recording methods** | | | | | | | | | | | | |
| --- | --- | --- | --- | --- | --- | --- | --- | --- | --- | --- | --- | --- |
|  |  |  |  |  |  |  |  |  |  |  |  |  |
|  | **Entire group [3-35 years; n=1685]** | | | **Children [3-12 years; n=728]** | | | **Adolescents [12-18 years; n=361]** | | | **Young adults [18-35 years; n=596]** | | |
| **cSBP** | RT- CT | RT -BOSC | CT - BOSC | RT- CT | RT -BOSC | CT - BOSC | RT- CT | RT - BOSC | CT - BOSC | RT- CT | RT - BOSC | CT - BOSC |
| r | 0.82 | 0.79 | 0.72 | 0.73 | 0.64 | 0.50 | 0.70 | 0.60 | 0.53 | 0.68 | 0.47 | 0.49 |
| p | **<0.001** | **<0.001** | **<0.001** | **<0.001** | **<0.001** | **<0.001** | **<0.001** | **<0.001** | **<0.001** | **<0.001** | **<0.001** | **<0.001** |
| Mean error (mmHg) | -8.0 | -5.2 | 3.1 | -7.5 | -3.9 | 4.2 | -9.3 | -6.5 | 3.6 | -7.4 | -5.5 | 1.5 |
| Mean error, CI 95% U.L. (mmHg) | -7.4 | -4.5 | 4.1 | -6.7 | -3.0 | 5.5 | -8.2 | -5.1 | 5.4 | -6.3 | -4.1 | 3.1 |
| Mean error, CI 95% L.L. (mmHg) | -8.6 | -6.0 | 2.2 | -8.3 | -4.9 | 2.8 | -10.5 | -7.9 | 1.8 | -8.4 | -7.0 | -0.1 |
| Mean error, p value | **<0.001** | **<0.001** | **<0.001** | **<0.001** | **<0.001** | **<0.001** | **<0.001** | **<0.001** | **<0.001** | **<0.001** | **<0.001** | 0.07 |
| Mean error, SD (mmHg) | 8.8 | 9.2 | 11.2 | 7.1 | 7.4 | 9.8 | 9.80 | 9.89 | 12.6 | 9.9 | 10.3 | 11.0 |
| CI 95%, Upper limit (mmHg) | 9.32 | 12.84 | 25.12 | 6.38 | 10.62 | 23.28 | 9.87 | 12.87 | 28.33 | 11.11 | 14.59 | 23.16 |
| CI 95%, Lower limit (mmHg) | -25.28 | -23.31 | -18.84 | -21.34 | -18.44 | -14.96 | -28.56 | -25.88 | -21.21 | -26.82 | -25.65 | -20.14 |
| Regression equation | y=12.9 - 0.2x | y=9.1 - 0.1x | y= -5.4 + 0.08x | y=10.0 - 0.2x | y=7.3 - 0.1x | y=-11.5+ 0.2x | y= 30.6 - 0.4x | y= 15.4 - 0.2x | y= -21.6 + 0.2x | y= 26.8 - 0.3x | y= 9.2 - 0.1x | y= -17.7 + 0.2x |
| p (Slope) | **<0.001** | **<0.001** | **0.03** | **<0.001** | **0.038** | **0.037** | **<0.001** | **0.003** | **0.004** | **<0.001** | 0.10 | **0.045** |
| **cPP** | RT- CT | RT -BOSC | CT - BOSC | RT- CT | RT -BOSC | CT - BOSC | RT- CT | RT - BOSC | CT - BOSC | RT- CT | RT - BOSC | CT - BOSC |
| r | 0.66 | 0.64 | 0.50 | 0.57 | 0.63 | 0.46 | 0.67 | 0.47 | 0.49 | 0.65 | 0.62 | 0.52 |
| p | **<0.001** | **<0.001** | **<0.001** | **<0.001** | **<0.001** | **<0.001** | **<0.001** | **<0.001** | **<0.001** | **<0.001** | **<0.001** | **<0.001** |
| Mean error (mmHg) | -9.2 | -2.4 | 7.3 | -8.9 | -2.9 | 6.9 | -10.7 | -2.6 | 8.6 | -8.3 | -1.6 | 6.5 |
| Mean error, CI 95% U.L. (mmHg) | -8.6 | -1.8 | 8.2 | -8.1 | -2.1 | 8.1 | -9.5 | -1.4 | 10.3 | -7.3 | -0.3 | 8.1 |
| Mean error, CI 95% L.L. (mmHg) | -9.8 | -3.1 | 6.4 | -9.7 | -3.7 | 5.7 | -11.9 | -3.9 | 6.8 | -9.4 | -2.9 | 4.8 |
| p | **<0.001** | **<0.001** | **<0.001** | **<0.001** | **<0.001** | **<0.001** | **<0.001** | **<0.001** | **<0.001** | **<0.001** | **0.015** | **<0.001** |
| Mean error, SD (mmHg) | 9.2 | 8.1 | 10.8 | 7.5 | 6.2 | 8.7 | 10.0 | 9.0 | 12.5 | 9.8 | 9.1 | 11.0 |
| CI 95%, Upper limit (mmHg) | 8.73 | 13.54 | 28.53 | 5.79 | 9.31 | 23.97 | 8.88 | 15.06 | 33.16 | 10.85 | 16.30 | 27.94 |
| CI 95%, Lower limit (mmHg) | -27.17 | -18.37 | -13.93 | -23.62 | -15.10 | -10.24 | -30.23 | -20.34 | -16.02 | -27.49 | -19.51 | -15.04 |
| Regression equation | y= 2.6 - 0.3x | y= 0.9 - 0.09x | y= -0.5 + 0.2x | y=0.7 - 0.3x | y= -2.1 - 0.03x | y= -2.0+ 0.3x | y= 9.6 - 0.5x | y= 6.5 -0.2x | y= -7.7 + 0.4x | y= 6.4 - 0.4x | y= 5.0 - 0.2x | y= 5.2 + 0.03x |
| p (Slope) | **<0.001** | **0.003** | **<0.001** | **<0.001** | 0.644 | **0.002** | **<0.001** | **0.001** | **<0.001** | **<0.001** | **0.012** | 0.724 |
| **Pf** | RT- CT | RT -BOSC | CT - BOSC | RT- CT | RT -BOSC | CT - BOSC | RT- CT | RT - BOSC | CT - BOSC | RT- CT | RT - BOSC | CT - BOSC |
| r | 0.57 | 0.57 | 0.35 | 0.54 | 0.62 | 0.44 | 0.57 | 0.59 | 0.44 | 0.60 | 0.58 | 0.47 |
| p | **<0.001** | **<0.001** | **<0.001** | **<0.001** | **<0.001** | **<0.001** | **<0.001** | **<0.001** | **<0.001** | **<0.001** | **<0.001** | **<0.001** |
| Mean error (mmHg) | -10.9 | 7.1 | 18.3 | -11.0 | 4.4 | 15.6 | -11.8 | 8.3 | 20.2 | -10.3 | 9.1 | 19.0 |
| Mean error, CI 95% U.L. (mmHg) | -10.2 | 7.7 | 19.3 | -9.8 | 5.2 | 17.1 | -10.4 | 9.4 | 22.1 | -9.0 | 10.2 | 20.7 |
| Mean error, CI 95% L.L. (mmHg) | -11.7 | 6.5 | 17.3 | -12.1 | 3.6 | 14.2 | -13.3 | 7.3 | 18.2 | -11.5 | 7.9 | 17.4 |
| p | **<0.001** | **<0.001** | **<0.001** | **<0.001** | **<0.001** | **<0.001** | **<0.001** | **<0.001** | **<0.001** | **<0.001** | **<0.001** | **<0.001** |
| Mean error, SD (mmHg) | 10.2 | 7.5 | 10.9 | 8.4 | 6.0 | 8.9 | 11.0 | 7.7 | 12.2 | 10.7 | 7.9 | 10.8 |
| CI 95%, Upper limit (mmHg) | 9.02 | 21.75 | 39.63 | 5.50 | 16.17 | 33.06 | 9.82 | 23.42 | 44.07 | 10.80 | 24.62 | 40.15 |
| CI 95%, Lower limit (mmHg) | -30.89 | -7.59 | -2.97 | -27.41 | -7.41 | -1.78 | -33.49 | -6.74 | -3.73 | -31.31 | -6.49 | -2.07 |
| Regression equation | y= 1.9 - 0.4x | y= -3.5 + 0.4x | y= -6.2 + 0.7x | y= -0.3 - 0.4x | y= -7.9 + 0.6x | y= -9.2+ 0.9x | y= 10.4 - 0.6x | y=2.2 + 0.2x | y= -9.8 + 0.8x | y= 4.9 - 0.4x | y=1.4 + 0.3x | y= -5.1 + 0.7x |
| p (Slope) | **<0.001** | **<0.001** | **<0.001** | **<0.001** | **<0.001** | **<0.001** | **<0.001** | **0.007** | **<0.001** | **<0.001** | **<0.001** | **<0.001** |
| **Pb** | RT- CT | RT -BOSC | CT - BOSC | RT- CT | RT -BOSC | CT - BOSC | RT- CT | RT - BOSC | CT - BOSC | RT- CT | RT - BOSC | CT - BOSC |
| r | 0.69 | 0.58 | 0.44 | 0.39 | 0.57 | 0.40 | 0.56 | 0.56 | 0.40 | 0.57 | 0.54 | 0.41 |
| p | **<0.001** | **<0.001** | **<0.001** | **<0.001** | **<0.001** | **<0.001** | **<0.001** | **<0.001** | **<0.001** | **<0.001** | **<0.001** | **<0.001** |
| Mean error (mmHg) | -1.9 | -0.7 | 1.0 | -0.9 | 0.3 | 1.3 | -2.2 | -1.6 | 0.6 | -2.4 | -0.9 | 1.2 |
| Mean error, CI 95% U.L. (mmHg) | -1.6 | -0.4 | 1.4 | -0.4 | 0.7 | 1.9 | -1.7 | -1.1 | 1.3 | -2.0 | -0.3 | 1.9 |
| Mean error, CI 95% L.L. (mmHg) | -2.2 | -1.0 | 0.6 | -1.3 | -0.1 | 0.8 | -2.7 | -2.2 | -0.2 | -2.9 | -1.5 | 0.4 |
| p | **<0.001** | **<0.001** | **<0.001** | **<0.001** | 0.093 | **<0.001** | **<0.001** | **<0.001** | 0.15 | **<0.001** | **0.002** | **0.002** |
| Mean error, SD (mmHg) | 3.8 | 3.8 | 4.5 | 3.5 | 2.8 | 3.6 | 3.8 | 4.1 | 4.9 | 3.8 | 4.3 | 4.9 |
| CI 95%, Upper limit (mmHg) | 5.52 | 6.75 | 9.88 | 6.03 | 5.83 | 8.44 | 5.25 | 6.32 | 10.15 | 5.03 | 7.39 | 10.72 |
| CI 95%, Lower limit (mmHg) | -9.31 | -8.13 | -7.82 | -7.79 | -5.21 | -5.76 | -9.72 | -9.58 | -9.01 | -9.89 | -9.29 | -8.34 |
| Regression equation | y= 3.8 - 0.4x | y= 4.9 - 0.4xx | y= 3.2 - 0.1x | y= 2.8 - 0.3x | y=1.7 - 0.1x | y= 1.5 -0.02x | y= 4.3 - 0.4x | y= 5.1 - 0.5x | y= 2.4 - 0.1x | y= 3.5- 0.4x | y= 7.3 - 0.6x | y= 5.5 - 0.3x |
| p (Slope) | **<0.001** | **<0.001** | **0.006** | **0.001** | 0.078 | 0.880 | **<0.001** | **<0.001** | 0.283 | **<0.001** | **<0.001** | **0.008** |
| RT: radial tonometry (SphygmoCor). CT: carotid tonometry (ShygmoCor). BOSC: brachial oscillometry/plethysmography (Mobil-O-Graph). cSBP, cPP: central systolic and pulse pressure, respectively. Pf and Pb: forward and backward wave amplitude, respectively. r: correlation (Pearson) coefficient. β: slope of regression equation. Significance: p<0.05 (red text). 'Bland-Altman: "x" was considered the mean of both methods compared (e.g. (RT+CT)/2); "y" the difference among first and second method (eg. RT minus CT). U.L and L.L: upper and lower limit, respectively. | | | | | | | | | | | | |
|  |  |  |  |  |  |  |  |  |  |  |  |  |
