# Supplementary material for: Aortic pressure and forward and backward wave components in children, adolescents and young-adults: Agreement between brachial oscillometry, radial and carotid tonometry data and analysis of factors associated with their differences
Source: PLoS One. 2019 Dec 19;14(12):e0226709. doi: 10.1371/journal.pone.0226709 (PMC6922407; doi:10.1371/journal.pone.0226709)
Supplement: S4 Table — (DOCX) [file pone.0226709.s022.docx]

| **S4 Table. cSBP: correlation and agreement among values obtained with three different recording methods** | | | | | | | | | | | | | |
| --- | --- | --- | --- | --- | --- | --- | --- | --- | --- | --- | --- | --- | --- |
|  |  |  |  |  |  |  |  |  |  |  |  |  |  |
| **cSBP** | | **Entire group [3-35 years]** | | | **Children [3-12 years]** | | | **Adolescents [12-18 years]** | | | **Young adults [18-35 years]** | | |
|  |  | **RT (SCOR)** | **CT (SCOR)** | **BOSC (MOG)** | **RT (SCOR)** | **CT (SCOR)** | **BOSC (MOG)** | **RT (SCOR)** | **CT (SCOR)** | **BOSC (MOG)** | **RT (SCOR)** | **CT (SCOR)** | **BOSC (MOG)** |
| **Radial tonometry (SCOR)** | r | ˗ | 0.82 | 0.79 | ˗ | 0.73 | 0.64 | ˗ | 0.70 | 0.60 | ˗ | 0.68 | 0.47 |
|  | p | ˗ | **<0.001** | **<0.001** | ˗ | **<0.001** | **<0.001** | ˗ | **<0.001** | **<0.001** | ˗ | **<0.001** | **<0.001** |
|  | Mean error (mmHg) | ˗ | -7.98 | -5.23 | ˗ | -7.48 | -3.91 | ˗ | -9.35 | -6.51 | ˗ | -7.35 | -5.53 |
|  | Mean error, CI 95% Upper Limit (mmHg) |  | -7.41 | -4.52 |  | -6.70 | -2.96 |  | -8.17 | -5.14 |  | -6.33 | -4.10 |
|  | Mean error, CI 95% Lower Limit (mmHg) | ˗ | -8.55 | -5.95 | ˗ | -8.26 | -4.85 | ˗ | -10.53 | -7.87 | ˗ | -8.38 | -6.96 |
|  | p | ˗ | **<0.001** | **<0.001** | ˗ | **<0.001** | **<0.001** | ˗ | **<0.001** | **<0.001** | ˗ | **<0.001** | **<0.001** |
|  | Mean error, SD (mmHg) | ˗ | 8.83 | 9.22 | ˗ | 7.07 | 7.41 | ˗ | 9.80 | 9.89 | ˗ | 9.93 | 10.26 |
|  | Upper limit (mmHg) | ˗ | 9.32 | 12.84 | ˗ | 6.38 | 10.62 | ˗ | 9.87 | 12.87 | ˗ | 11.11 | 14.59 |
|  | Lower limit (mmHg) | ˗ | -25.28 | -23.31 | ˗ | -21.34 | -18.44 | ˗ | -28.56 | -25.88 | ˗ | -26.82 | -25.65 |
|  | Regression equation | ˗ | y=12.9 - 0.2x | y=9.1 - 0.1x | ˗ | y=10.0 - 0.2x | y=7.3 - 0.1x | ˗ | y= 30.6 - 0.4x | y= 15.4 - 0.2x | ˗ | y= 26.8 - 0.3x | y= 9.2 - 0.1x |
|  | p(ϐ) | ˗ | **<0.001** | **<0.001** | ˗ | **<0.001** | **0.04** | ˗ | **<0.001** | **0.00** | ˗ | **<0.001** | 0.10 |
| **Carotid tonometry (SCOR)** | r | 0.82 | ˗ | 0.72 | 0.73 | ˗ | 0.50 | 0.70 | ˗ | 0.53 | 0.68 | ˗ | 0.49 |
|  | p | **<0.001** | ˗ | **<0.001** | **<0.001** | ˗ | **<0.001** | **<0.001** | ˗ | **<0.001** | **<0.001** | ˗ | **<0.001** |
|  | Mean error (mmHg) | 7.98 | ˗ | 3.14 | 7.48 | ˗ | 4.16 | 9.35 | ˗ | 3.56 | 7.35 | ˗ | 1.51 |
|  | Mean error, CI 95% Upper Limit (mmHg) | 8.55 |  | 4.05 | 8.26 |  | 5.50 | 10.53 |  | 5.35 | 8.38 |  | 3.13 |
|  | Mean error, CI 95% Lower Limit (mmHg) | 7.41 | ˗ | 2.23 | 6.70 | ˗ | 2.83 | 8.17 | ˗ | 1.77 | 6.33 | ˗ | -0.11 |
|  | p | **<0.001** | ˗ | **<0.001** | **<0.001** | ˗ | **<0.001** | **<0.001** | ˗ | **<0.001** | **<0.001** | ˗ | 0.07 |
|  | Mean error, SD (mmHg) | 8.83 | ˗ | 11.22 | 7.07 | ˗ | 9.76 | 9.80 | ˗ | 12.64 | 9.42 | ˗ | 11.04 |
|  | Upper limit (mmHg) | 25.28 | ˗ | 25.12 | 21.34 | ˗ | 23.28 | 28.56 | ˗ | 28.33 | 25.82 | ˗ | 23.16 |
|  | Lower limit (mmHg) | -9.32 | ˗ | -18.84 | -6.38 | ˗ | -14.96 | -9.87 | ˗ | -21.21 | -11.11 | ˗ | -20.14 |
|  | Regression equation | y=-12.9 + 0.2x | ˗ | y= -5.4 + 0.08x | y=-10.0 + 0.2x | ˗ | y=-11.5+ 0.2x | y= -30.6 + 0.4x | ˗ | y= -21.6 + 0.2x | y= -26.8 + 0.3x | ˗ | y= -17.7 + 0.2x |
|  | p(ϐ) | **<0.001** | ˗ | **0.03** | **<0.001** | ˗ | **0.04** | **<0.001** | ˗ | **0.00** | **<0.001** | ˗ | **0.05** |
| **Brachial oscillometry (MOG)** | r | 0.79 | 0.72 | ˗ | 0.64 | 0.50 | ˗ | 0.60 | 0.53 | ˗ | 0.47 | 0.49 | ˗ |
|  | p | **<0.001** | **<0.001** | ˗ | **<0.001** | **<0.001** | ˗ | **<0.001** | **<0.001** | ˗ | **<0.001** | **<0.001** | ˗ |
|  | Mean error (mmHg) | 5.23 | -3.14 | ˗ | 3.91 | -4.16 | ˗ | 6.51 | -3.56 | ˗ | 5.53 | -1.51 | ˗ |
|  | Mean error, CI 95% Upper Limit (mmHg) | 5.95 | -2.23 |  | 4.85 | -2.83 |  | 7.87 | -1.77 |  | 6.96 | 0.11 |  |
|  | Mean error, CI 95% Lower Limit (mmHg) | 4.52 | -4.05 | ˗ | 2.96 | -5.50 | ˗ | 5.14 | -5.35 | ˗ | 4.10 | -3.13 | ˗ |
|  | p | **<0.001** | **<0.001** | ˗ | **<0.001** | **<0.001** | ˗ | **<0.001** | **<0.001** | ˗ | **<0.001** | 0.07 | ˗ |
|  | Mean error, SD (mmHg) | 9.22 | 11.22 | ˗ | 7.41 | 9.76 | ˗ | 9.89 | 12.64 | ˗ | 10.26 | 11.04 | ˗ |
|  | Upper limit (mmHg) | 23.31 | 18.84 | ˗ | 18.44 | 14.96 | ˗ | 25.88 | 21.21 | ˗ | 25.65 | 20.14 | ˗ |
|  | Lower limit (mmHg) | -12.84 | -25.12 | ˗ | -10.62 | -23.28 | ˗ | -12.87 | -28.33 | ˗ | -14.59 | -23.16 | ˗ |
|  | Regression equation | y=9.1 - 0.1x | y= 5.4 - 0.08x | ˗ | y= -7.3+ 0.1x | y=11.5 - 0.2x | ˗ | y= -15.4 + 0.2x | y= 21.6 - 0.2x | ˗ | y= -9.2 + 0.1x | y= 17.7 - 0.2x | ˗ |
|  | p(ϐ) | **<0.001** | **0.03** | ˗ | **0.04** | **0.04** | ˗ | **0.00** | **0.00** | ˗ | 0.10 | **0.05** | ˗ |
| RT: radial applanation tonometry record, obtained with SphygmoCor device (SCOR). CT: carotid applanation tonometry record, obtained with SCOR. BOSC: brachial oscillometry/plethysmography record, obtained with Mobil-O-Graph device (MOG). cSBP: central (aortic) systolic blood pressure. r: correlation (Pearson) coefficient. β: slope of regression equation. CI: confidence interval. Significance level: p value <0.05 (red text). 'Bland-Altman analysis: variable "x" was considered the mean of both methods compared (eg. (RT+CT)/2) and variable "y" the difference among first and second method (eg. RT minus CT); first method in rows and second method in columns. | | | | | | | | | | | | | |
|  |  |  |  |  |  |  |  |  |  |  |  |  |  |
|  |  |  |  |  |  |  |  |  |  |  |  |  |  |
