# Supplementary material for: Aortic pressure and forward and backward wave components in children, adolescents and young-adults: Agreement between brachial oscillometry, radial and carotid tonometry data and analysis of factors associated with their differences
Source: PLoS One. 2019 Dec 19;14(12):e0226709. doi: 10.1371/journal.pone.0226709 (PMC6922407; doi:10.1371/journal.pone.0226709)
Supplement: S5 Table — (DOCX) [file pone.0226709.s023.docx]

| **S5 Table. cPP: correlation and agreement among values obtained with three different recording methods** | | | | | | | | | | | | | |
| --- | --- | --- | --- | --- | --- | --- | --- | --- | --- | --- | --- | --- | --- |
|  |  |  |  |  |  |  |  |  |  |  |  |  |  |
| **cPP** | | **Entire group [3-35 years]** | | | **Children [3-12 years]** | | | **Adolescents [12-18 years]** | | | **Young adults [18-35 years]** | | |
|  |  | **RT (SCOR)** | **CT (SCOR)** | **BOSC (MOG)** | **RT (SCOR)** | **CT (SCOR)** | **BOSC (MOG)** | **RT (SCOR)** | **CT (SCOR)** | **BOSC (MOG)** | **RT (SCOR)** | **CT (SCOR)** | **BOSC (MOG)** |
| **Radial tonometry (SCOR)** | r | ˗ | 0.66 | 0.64 | ˗ | 0.57 | 0.63 | ˗ | 0.67 | 0.47 | ˗ | 0.65 | 0.62 |
|  | p | ˗ | **<0.001** | **<0.001** | ˗ | **<0.001** | **<0.001** | ˗ | **<0.001** | **<0.001** | ˗ | **<0.001** | **<0.001** |
|  | Mean error (mmHg) | ˗ | -9.22 | -2.42 | ˗ | -8.91 | -2.90 | ˗ | -10.67 | -2.64 | ˗ | -8.32 | -1.61 |
|  | Mean error, CI 95% Upper Limit (mmHg) |  | -8.62 | -1.78 |  | -8.09 | -2.10 |  | -9.48 | -1.39 |  | -7.26 | -0.32 |
|  | Mean error, CI 95% Lower Limit (mmHg) | ˗ | -9.81 | -3.05 | ˗ | -9.74 | -3.69 | ˗ | -11.87 | -3.90 | ˗ | -9.38 | -2.89 |
|  | p | ˗ | **<0.001** | **<0.001** | ˗ | **<0.001** | **<0.001** | ˗ | **<0.001** | **<0.001** | ˗ | **<0.001** | **0.01** |
|  | Mean error, SD (mmHg) | ˗ | 9.16 | 8.14 | ˗ | 7.50 | 6.23 | ˗ | 9.98 | 9.03 | ˗ | 9.78 | 9.14 |
|  | Upper limit (mmHg) | ˗ | 8.73 | 13.54 | ˗ | 5.79 | 9.31 | ˗ | 8.88 | 15.06 | ˗ | 10.85 | 16.30 |
|  | Lower limit (mmHg) | ˗ | -27.17 | -18.37 | ˗ | -23.62 | -15.10 | ˗ | -30.23 | -20.34 | ˗ | -27.49 | -19.51 |
|  | Regression equation | ˗ | y= 2.6 - 0.3x | y= 0.9 - 0.09x | ˗ | y=0.7 - 0.3x | y= -2.1 - 0.03x | ˗ | y= 9.6 - 0.5x | y= 6.5 -0.2x | ˗ | y= 6.4 - 0.4x | y= 5.0 - 0.2x |
|  | p(ϐ) | ˗ | **<0.001** | **0.00** | ˗ | **<0.001** | 0.64 | ˗ | **<0.001** | **0.00** | ˗ | **<0.001** | **0.01** |
| **Carotid tonometry (SCOR)** | r | 0.66 | ˗ | 0.50 | 0.57 | ˗ | 0.46 | 0.67 | ˗ | 0.49 | 0.65 | ˗ | 0.52 |
|  | p | **<0.001** | ˗ | **<0.001** | **<0.001** | ˗ | **<0.001** | **<0.001** | ˗ | **<0.001** | **<0.001** | ˗ | **<0.001** |
|  | Mean error (mmHg) | 9.22 | ˗ | 7.30 | 8.91 | ˗ | 6.86 | 10.67 | ˗ | 8.57 | 8.32 | ˗ | 6.45 |
|  | Mean error, CI 95% Upper Limit (mmHg) | 9.81 | ˗ | 8.18 | 9.74 |  | 8.06 | 11.87 |  | 10.35 | 9.38 |  | 8.05 |
|  | Mean error, CI 95% Lower Limit (mmHg) | 8.62 |  | 6.42 | 8.09 | ˗ | 5.67 | 9.48 | ˗ | 6.80 | 7.26 | ˗ | 4.85 |
|  | p | **<0.001** | ˗ | **<0.001** | **<0.001** | ˗ | **<0.001** | **<0.001** | ˗ | **<0.001** | **<0.001** | ˗ | **<0.001** |
|  | Mean error, SD (mmHg) | 9.16 | ˗ | 10.83 | 7.50 | ˗ | 8.73 | 9.98 | ˗ | 12.55 | 9.78 | ˗ | 10.96 |
|  | Upper limit (mmHg) | 27.17 | ˗ | 28.53 | 23.62 | ˗ | 23.97 | 30.23 | ˗ | 33.16 | 27.49 | ˗ | 27.94 |
|  | Lower limit (mmHg) | -8.73 | ˗ | -13.93 | -5.79 | ˗ | -10.24 | -8.88 | ˗ | -16.02 | -10.85 | ˗ | -15.04 |
|  | Regression equation | y= -2.6 + 0.3x | ˗ | y= -0.5 + 0.2x | y= -0.7 + 0.3x | ˗ | y= -2.0+ 0.3x | y= -9.6 + 0.5x | ˗ | y= -7.7 + 0.4x | y= -6.4+ 0.4x | ˗ | y= 5.2 + 0.03x |
|  | p(ϐ) | **<0.001** | ˗ | **<0.001** | **<0.001** | ˗ | **0.00** | **<0.001** | ˗ | **<0.001** | **<0.001** | ˗ | 0.72 |
| **Brachial oscillometry (MOG)** | r | 0.64 | 0.50 | ˗ | 0.63 | 0.46 | ˗ | 0.47 | 0.49 | ˗ | 0.62 | 0.52 | ˗ |
|  | p | **<0.001** | **<0.001** | ˗ | **<0.001** | **<0.001** | ˗ | **<0.001** | **<0.001** | ˗ | **<0.001** | **<0.001** | ˗ |
|  | Mean error (mmHg) | 2.42 | 7.30 | ˗ | 2.90 | -6.86 | ˗ | 2.64 | -8.57 | ˗ | 1.61 | -6.45 | ˗ |
|  | Mean error, CI 95% Upper Limit (mmHg) | 3.05 | -6.42 |  | 3.69 | -5.67 |  | 3.90 | -6.80 |  | 2.89 | -4.85 |  |
|  | Mean error, CI 95% Lower Limit (mmHg) | 1.78 | -8.18 | ˗ | 2.10 | -8.06 | ˗ | 1.39 | -10.35 | ˗ | 0.32 | -8.05 | ˗ |
|  | p | **<0.001** | **<0.001** | ˗ | **<0.001** | **<0.001** | ˗ | **<0.001** | **<0.001** | ˗ | **0.01** | **<0.001** | ˗ |
|  | Mean error, SD (mmHg) | 8.14 | 10.83 | ˗ | 6.23 | 8.73 | ˗ | 9.03 | 12.55 | ˗ | 9.14 | 10.96 | ˗ |
|  | Upper limit (mmHg) | 18.37 | 13.93 | ˗ | 15.10 | 10.24 | ˗ | 20.34 | 16.02 | ˗ | 19.51 | 15.04 | ˗ |
|  | Lower limit (mmHg) | -13.54 | -28.53 | ˗ | -9.31 | -23.97 | ˗ | -15.06 | -33.16 | ˗ | -16.30 | -27.94 | ˗ |
|  | Regression equation | y= -0.9 + 0.09x | y= 0.5 - 0.2x | ˗ | y= 2.1 + 0.03x | y= 2.0 - 0.3x | ˗ | y= -6.5+ 0.2x | y= 7.7 - 0.4x | ˗ | y= -5.0 + 0.2x | y= -5.2 - 0.03x | ˗ |
|  | p(ϐ) | **0.00** | **<0.001** | ˗ | 0.64 | **0.00** | ˗ | **0.00** | **<0.001** | ˗ | **0.01** | 0.72 | ˗ |
| RT: radial applanation tonometry record, obtained with SphygmoCor device (SCOR). CT: carotid applanation tonometry record, obtained with SCOR. BOSC: brachial oscillometry/plethysmography record, obtained with Mobil-O-Graph device (MOG). cPP: central (aortic) pulse blood pressure. r: correlation (Pearson) coefficient. β: slope of regression equation. CI: confidence interval. Significance level: p value <0.05 (red text). 'Bland-Altman analysis: variable "x" was considered the mean of both methods compared (eg. (RT+CT)/2) and variable "y" the difference among first and second method (eg. RT minus CT); first method in rows and second method in columns. | | | | | | | | | | | | | |
|  |  |  |  |  |  |  |  |  |  |  |  |  |  |
|  |  |  |  |  |  |  |  |  |  |  |  |  |  |
|  |  |  |  |  |  |  |  |  |  |  |  |  |  |
