# Supplementary material for: Aortic pressure and forward and backward wave components in children, adolescents and young-adults: Agreement between brachial oscillometry, radial and carotid tonometry data and analysis of factors associated with their differences
Source: PLoS One. 2019 Dec 19;14(12):e0226709. doi: 10.1371/journal.pone.0226709 (PMC6922407; doi:10.1371/journal.pone.0226709)
Supplement: S6 Table — (DOCX) [file pone.0226709.s024.docx]

| **S6 Table. Pf: correlation and agreement among values obtained with three different recording methods** | | | | | | | | | | | | | |
| --- | --- | --- | --- | --- | --- | --- | --- | --- | --- | --- | --- | --- | --- |
|  |  |  |  |  |  |  |  |  |  |  |  |  |  |
| **Pf** | | **Entire group [3-35 years]** | | | **Children [3-12 years]** | | | **Adolescents [12-18 years]** | | | **Young adults [18-35 years]** | | |
|  |  | **RT (SCOR)** | **CT (SCOR)** | **BOSC (MOG)** | **RT (SCOR)** | **CT (SCOR)** | **BOSC (MOG)** | **RT (SCOR)** | **CT (SCOR)** | **BOSC (MOG)** | **RT (SCOR)** | **CT (SCOR)** | **BOSC (MOG)** |
| **Radial tonometry (SCOR)** | r | ˗ | 0.57 | 0.57 | ˗ | 0.54 | 0.62 | ˗ | 0.57 | 0.59 | ˗ | 0.60 | 0.58 |
|  | p | ˗ | **<0.001** | **<0.001** | ˗ | **<0.001** | **<0.001** | ˗ | **<0.001** | **<0.001** | ˗ | **<0.001** | **<0.001** |
|  | Mean error (mmHg) | ˗ | -10.93 | 7.08 | ˗ | -10.95 | 4.38 | ˗ | -11.84 | 8.34 | ˗ | -10.25 | 9.06 |
|  | Mean error, CI 95% Upper Limit (mmHg) |  | -10.20 | 7.67 |  | -9.85 | 5.16 |  | -10.35 | 9.42 |  | -9.01 | 10.19 |
|  | Mean error, CI 95% Lower Limit (mmHg) | ˗ | -11.67 | 6.49 | ˗ | -12.05 | 3.61 | ˗ | -13.32 | 7.26 | ˗ | -11.49 | 7.93 |
|  | p | ˗ | **<0.001** | **<0.001** | ˗ | **<0.001** | **<0.001** | ˗ | **<0.001** | **<0.001** | ˗ | **<0.001** | **<0.001** |
|  | Mean error, SD (mmHg) | ˗ | 10.18 | 7.48 | ˗ | 8.39 | 6.02 | ˗ | 11.05 | 7.70 | ˗ | 10.74 | 7.94 |
|  | Upper limit (mmHg) | ˗ | 9.02 | 21.75 | ˗ | 5.50 | 16.17 | ˗ | 9.82 | 23.42 | ˗ | 10.80 | 24.62 |
|  | Lower limit (mmHg) | ˗ | -30.89 | -7.59 | ˗ | -27.41 | -7.41 | ˗ | -33.49 | -6.74 | ˗ | -31.31 | -6.49 |
|  | Regression equation | ˗ | y= 1.9 - 0.4x | y= -3.5 + 0.4x | ˗ | y= -0.3 - 0.4x | y= -7.9 + 0.6x | ˗ | y= 10.4 - 0.6x | y=2.2 + 0.2x | ˗ | y= 4.9 - 0.4x | y=1.4 + 0.3x |
|  | p(ϐ) | ˗ | **<0.001** | **<0.001** | ˗ | **<0.001** | **<0.001** | ˗ | **<0.001** | **0.01** | ˗ | **<0.001** | **<0.001** |
| **Carotid tonometry (SCOR)** | r | 0.57 | ˗ | 0.35 | 0.54 | ˗ | 0.44 | 0.57 | ˗ | 0.44 | 0.60 | ˗ | 0.47 |
|  | p | **<0.001** | ˗ | **<0.001** | **<0.001** | ˗ | **<0.001** | **<0.001** | ˗ | **<0.001** | **<0.001** | ˗ | **<0.001** |
|  | Mean error (mmHg) | 10.93 | ˗ | 18.33 | 10.95 | ˗ | 15.64 | 11.84 | ˗ | 20.17 | 10.25 | ˗ | 19.04 |
|  | Mean error, CI 95% Upper Limit (mmHg) | 10.20 | ˗ | 19.31 | 12.05 |  | 17.07 | 13.32 |  | 22.11 | 11.49 |  | 20.70 |
|  | Mean error, CI 95% Lower Limit (mmHg) | 11.67 |  | 17.34 | 9.85 | ˗ | 14.21 | 10.35 | ˗ | 18.24 | 9.01 | ˗ | 17.39 |
|  | p | **<0.001** | ˗ | **<0.001** | **<0.001** | ˗ | **<0.001** | **<0.001** | ˗ | **<0.001** | **<0.001** | ˗ | **<0.001** |
|  | Mean error, SD (mmHg) | 10.18 | ˗ | 10.87 | 8.39 | ˗ | 8.89 | 11.05 | ˗ | 12.19 | 10.74 | ˗ | 10.77 |
|  | Upper limit (mmHg) | -9.02 | ˗ | 39.63 | 27.41 | ˗ | 33.06 | 33.49 | ˗ | 44.07 | 31.31 | ˗ | 40.15 |
|  | Lower limit (mmHg) | 30.89 | ˗ | -2.97 | -5.50 | ˗ | -1.78 | -9.82 | ˗ | -3.73 | -10.80 | ˗ | -2.07 |
|  | Regression equation | y= -1.9 + 0.4x | ˗ | y= -6.2 + 0.7x | y= 0.3 + 0.4x | ˗ | y= -9.2+ 0.9x | y= -10.4 + 0.6x | ˗ | y= -9.8 + 0.8x | y=-4.9 + 0.4x | ˗ | y= -5.1 + 0.7x |
|  | p(ϐ) | **<0.001** | ˗ | **<0.001** | **<0.001** | ˗ | **<0.001** | **<0.001** | ˗ | **<0.001** | **<0.001** | ˗ | **<0.001** |
| **Brachial oscillometry (MOG)** | r | 0.57 | 0.35 | ˗ | 0.62 | 0.44 | ˗ | 0.59 | 0.44 | ˗ | 0.58 | 0.47 | ˗ |
|  | p | **<0.001** | **<0.001** | ˗ | **<0.001** | **<0.001** | ˗ | **<0.001** | **<0.001** | ˗ | **<0.001** | **<0.001** | ˗ |
|  | Mean error (mmHg) | -7.08 | -18.33 | ˗ | -4.38 | -15.64 | ˗ | -8.34 | 20.17 | ˗ | -9.06 | -19.04 | ˗ |
|  | Mean error, CI 95% Upper Limit (mmHg) | -7.67 | -19.31 |  | -3.61 | -14.21 |  | -7.26 | -18.24 |  | -7.93 | -17.39 |  |
|  | Mean error, CI 95% Lower Limit (mmHg) | -6.49 | -17.34 | ˗ | -5.16 | -17.07 | ˗ | -9.42 | 22.11 | ˗ | -10.19 | -20.70 | ˗ |
|  | p | **<0.001** | **<0.001** | ˗ | **<0.001** | **<0.001** | ˗ | **<0.001** | **<0.001** | ˗ | **<0.001** | **<0.001** | ˗ |
|  | Mean error, SD (mmHg) | 7.48 | 10.87 | ˗ | 6.02 | 8.89 | ˗ | 7.70 | 32.78 | ˗ | 7.94 | 10.77 | ˗ |
|  | Upper limit (mmHg) | -21.75 | -39.63 | ˗ | 7.41 | 1.78 | ˗ | 6.74 | 3.73 | ˗ | 6.49 | 2.07 | ˗ |
|  | Lower limit (mmHg) | 7.59 | 2.97 | ˗ | -16.17 | -33.06 | ˗ | -23.42 | -44.07 | ˗ | -24.62 | -40.15 | ˗ |
|  | Regression equation | y= 3.5 - 0.4x | y= 6.2 - 0.7x | ˗ | y= 7.9 - 0.6x | y= 9.2 - 0.9x | ˗ | y= -2.2 - 0.2 | y= 9.8 - 0.8x | ˗ | y=-1.4 - 0.3x | y= 5.1 - 0.7x | ˗ |
|  | p(ϐ) | **<0.001** | **<0.001** | ˗ | **<0.001** | **<0.001** | ˗ | **0.01** | **<0.001** | ˗ | **<0.001** | **<0.001** | ˗ |
| RT: radial applanation tonometry record, obtained with SphygmoCor device (SCOR). CT: carotid applanation tonometry record, obtained with SCOR. BOSC: brachial oscillometry/plethysmography record, obtained with Mobil-O-Graph device (MOG). Pf: forward wave height (amplitude) at the aortic level. r: correlation (Pearson) coefficient. β: slope of regression equation. CI: confidence interval. Significance level: p value <0.05 (red text). 'Bland-Altman analysis: variable "x" was considered the mean of both methods compared (eg. (RT+CT)/2) and variable "y" the difference among first and second method (eg. RT minus CT); first method in rows and second method in columns. | | | | | | | | | | | | | |
|  |  |  |  |  |  |  |  |  |  |  |  |  |  |
|  |  |  |  |  |  |  |  |  |  |  |  |  |  |
|  |  |  |  |  |  |  |  |  |  |  |  |  |  |
