# Supplementary material for: Aortic pressure and forward and backward wave components in children, adolescents and young-adults: Agreement between brachial oscillometry, radial and carotid tonometry data and analysis of factors associated with their differences
Source: PLoS One. 2019 Dec 19;14(12):e0226709. doi: 10.1371/journal.pone.0226709 (PMC6922407; doi:10.1371/journal.pone.0226709)
Supplement: S7 Table — (DOCX) [file pone.0226709.s025.docx]

| **S7 Table. Pb: correlation and agreement among values obtained with three different recording methods** | | | | | | | | | | | | | |
| --- | --- | --- | --- | --- | --- | --- | --- | --- | --- | --- | --- | --- | --- |
|  |  |  |  |  |  |  |  |  |  |  |  |  |  |
| **Pb** | | **Entire group [3-35 years]** | | | **Children [3-12 years]** | | | **Adolescents [12-18 years]** | | | **Young adults [18-35 years]** | | |
|  |  | **RT (SCOR)** | **CT (SCOR)** | **BOSC (MOG)** | **RT (SCOR)** | **CT (SCOR)** | **BOSC (MOG)** | **RT (SCOR)** | **CT (SCOR)** | **BOSC (MOG)** | **RT (SCOR)** | **CT (SCOR)** | **BOSC (MOG)** |
| **Radial tonometry (SCOR)** | r | ˗ | 0.69 | 0.58 | ˗ | 0.39 | 0.57 | ˗ | 0.56 | 0.56 | ˗ | 0.57 | 0.54 |
|  | p | ˗ | **<0.001** | **<0.001** | ˗ | **<0.001** | **<0.001** | ˗ | **<0.001** | **<0.001** | ˗ | **<0.001** | **<0.001** |
|  | Mean error (mmHg) | ˗ | -1.90 | -0.69 | ˗ | -0.88 | 0.31 | ˗ | -2.24 | -1.63 | ˗ | -2.43 | -0.95 |
|  | Mean error, CI 95% Upper Limit (mmHg) | ˗ | -1.62 | -0.39 |  | -1.34 | -0.05 | ˗ | -2.75 | -2.20 | ˗ | -2.87 | -1.55 |
|  | Mean error, CI 95% Lower Limit (mmHg) |  | -2.17 | -0.99 | ˗ | -0.42 | 0.67 |  | -1.72 | -1.06 |  | -1.99 | -0.35 |
|  | p | ˗ | **<0.001** | **<0.001** | ˗ | **<0.001** | 0.09 | ˗ | **<0.001** | **<0.001** | ˗ | **<0.001** | **0.00** |
|  | Mean error, SD (mmHg) | ˗ | 3.78 | 3.80 | ˗ | 3.52 | 2.81 | ˗ | 3.82 | 4.06 | ˗ | 3.81 | 4.26 |
|  | Upper limit (mmHg) | ˗ | 5.52 | 6.75 | ˗ | 6.03 | 5.83 | ˗ | 5.25 | 6.32 | ˗ | 5.03 | 7.39 |
|  | Lower limit (mmHg) | ˗ | -9.31 | -8.13 | ˗ | -7.79 | -5.21 | ˗ | -9.72 | -9.58 | ˗ | -9.89 | -9.29 |
|  | Regression equation | ˗ | y= 3.8 - 0.4x | y= 4.9 - 0.4x | ˗ | y= 2.8 - 0.3x | y=1.7 - 0.1x | ˗ | y= 4.3 - 0.4x | y= 5.1 - 0.5x | ˗ | y= 3.5- 0.4x | y= 7.3 - 0.6x |
|  | p(ϐ) | ˗ | **<0.001** | **<0.001** | ˗ | **0.00** | 0.08 | ˗ | **<0.001** | **<0.001** | ˗ | **<0.001** | **<0.001** |
| **Carotid tonometry (SCOR)** | r | 0.69 | ˗ | 0.44 | 0.39 | ˗ | 0.40 | 0.56 | ˗ | 0.40 | 0.57 | ˗ | 0.41 |
|  | p | **<0.001** | ˗ | **<0.001** | **<0.001** | ˗ | **<0.001** | **<0.001** | ˗ | **<0.001** | **<0.001** | ˗ | **<0.001** |
|  | Mean error (mmHg) | 1.90 | ˗ | 1.03 | 0.88 | ˗ | 1.34 | 2.24 | ˗ | 0.57 | 2.43 | ˗ | 1.19 |
|  | Mean error, CI 95% Upper Limit (mmHg) | 2.17 | ˗ | 1.44 | 0.42 | ˗ | 0.75 | 1.72 | ˗ | -0.20 | 1.99 | ˗ | 0.44 |
|  | Mean error, CI 95% Lower Limit (mmHg) | 1.62 |  | 0.62 | 1.34 |  | 1.92 | 2.75 |  | 1.34 | 2.87 |  | 1.93 |
|  | p | **<0.001** | ˗ | **<0.001** | **<0.001** | ˗ | **<0.001** | **<0.001** | ˗ | 0.15 | **<0.001** | ˗ | **0.00** |
|  | Mean error, SD (mmHg) | 3.78 | ˗ | 4.52 | 3.52 | ˗ | 3.62 | 3.82 | ˗ | 4.89 | 3.81 | ˗ | 4.86 |
|  | Upper limit (mmHg) | 9.31 | ˗ | 9.88 | 7.79 | ˗ | 8.44 | 9.72 | ˗ | 10.15 | 9.89 | ˗ | 10.72 |
|  | Lower limit (mmHg) | -5.52 | ˗ | -7.82 | -6.03 | ˗ | -5.76 | -5.25 | ˗ | -9.01 | -5.03 | ˗ | -8.34 |
|  | Regression equation | y= -3.8 + 0.4x | ˗ | y= 3.2 - 0.1x | y= -2.8 + 0.3x | ˗ | y= 1.5 -0.02x | y= -4.3 + 0.4x | ˗ | y= 2.4 - 0.1x | y= -3.5 + 0.4x | ˗ | y= 5.5 - 0.3x |
|  | p(ϐ) | **<0.001** | ˗ | **0.01** | **0.00** | ˗ | 0.88 | **<0.001** | ˗ | 0.28 | **<0.001** | ˗ | **0.01** |
| **Brachial oscillometry (MOG)** | r | 0.58 | 0.44 | ˗ | 0.57 | 0.40 | ˗ | 0.56 | 0.40 | ˗ | 0.54 | 0.41 | ˗ |
|  | p | **<0.001** | **<0.001** | ˗ | **<0.001** | **<0.001** | ˗ | **<0.001** | **<0.001** | ˗ | **<0.001** | **<0.001** | ˗ |
|  | Mean error (mmHg) | 0.69 | -1.03 | ˗ | -0.31 | -1.34 | ˗ | 1.63 | -0.57 | ˗ | 0.95 | -1.19 | ˗ |
|  | Mean error, CI 95% Upper Limit (mmHg) | 0.99 | -0.62 |  | -0.67 | -1.92 | ˗ | 1.06 | -1.34 | ˗ | 0.35 | -1.93 |  |
|  | Mean error, CI 95% Lower Limit (mmHg) | 0.39 | -1.44 | ˗ | 0.05 | -0.75 |  | 2.20 | 0.20 |  | 1.55 | -0.44 | ˗ |
|  | p | **<0.001** | **<0.001** |  | 0.09 | **<0.001** | ˗ | **<0.001** | 0.15 | ˗ | **0.00** | **0.00** | ˗ |
|  | Mean error, SD (mmHg) | 3.80 | 4.52 | ˗ | 2.81 | 3.62 | ˗ | 4.06 | 4.89 | ˗ | 4.26 | 4.86 | ˗ |
|  | Upper limit (mmHg) | 8.13 | 7.82 | ˗ | 5.21 | 5.76 | ˗ | 9.58 | 9.01 | ˗ | 9.29 | 8.34 | ˗ |
|  | Lower limit (mmHg) | -6.75 | -9.88 | ˗ | -5.83 | -8.44 | ˗ | -6.32 | -10.15 | ˗ | -7.39 | -10.72 | ˗ |
|  | Regression equation | y= -4.9 + 0.4x | y= -3.2 + 0.1x | ˗ | y=-1.7 + 0.1x | y= -1.5 + 0.02x | ˗ | y= -5.1+ 0.5x | y= -2.4 + 0.1x | ˗ | y= -7.3 + 0.6x | y= -5.5 + 0.3x | ˗ |
|  | p(ϐ) | **<0.001** | **0.01** | ˗ | 0.08 | 0.88 | ˗ | **<0.001** | 0.28 | ˗ | **<0.001** | **0.01** | ˗ |
| RT: radial applanation tonometry record, obtained with SphygmoCor device (SCOR). CT: carotid applanation tonometry record, obtained with SCOR. BOSC: brachial oscillometry/plethysmography record, obtained with Mobil-O-Graph device (MOG). Pb: backward wave height (amplitude) at the aortic level. r: correlation (Pearson) coefficient. β: slope of regression equation. CI: confidence interval. Significance level: p value <0.05 (red text). 'Bland-Altman analysis: variable "x" was considered the mean of both methods compared (eg. (RT+CT)/2) and variable "y" the difference among first and second method (eg. RT minus CT); first method in rows and second method in columns. | | | | | | | | | | | | | |
|  |  |  |  |  |  |  |  |  |  |  |  |  |  |
|  |  |  |  |  |  |  |  |  |  |  |  |  |  |
|  |  |  |  |  |  |  |  |  |  |  |  |  |  |
