# Supplementary material for: Aortic pressure and forward and backward wave components in children, adolescents and young-adults: Agreement between brachial oscillometry, radial and carotid tonometry data and analysis of factors associated with their differences
Source: PLoS One. 2019 Dec 19;14(12):e0226709. doi: 10.1371/journal.pone.0226709 (PMC6922407; doi:10.1371/journal.pone.0226709)
Supplement: S8 Table — (DOCX) [file pone.0226709.s026.docx]

| **S8 Table. Clinical features and cardiovascular risk factors for the entire and age-related subgroups: subsample** | | | | | | | | | | | | | | | | | | | | | | | | |
| --- | --- | --- | --- | --- | --- | --- | --- | --- | --- | --- | --- | --- | --- | --- | --- | --- | --- | --- | --- | --- | --- | --- | --- | --- |
|  |  |  |  |  |  |  |  |  |  |  |  |  |  |  |  |  |  |  |  |  |  |  |  |  |
|  | **Entire group [n=266]** | | | | | | **Children [3-12 years; n=83]** | | | | | | **Adolescents [12-18 years; n=97]** | | | | | | **Young adults [18-35 years; n=86]** | | | | | |
|  | **MV** | **SD** | **Min** | **p25th** | **p75th** | **Max** | **MV** | **SD** | **Min** | **p25th** | **p75th** | **Max** | **MV** | **SD** | **Min** | **p25th** | **p75th** | **Max** | **MV** | **SD** | **Min** | **p25th** | **p75th** | **Max** |
|  |  |  |  |  |  |  |  |  |  |  |  |  |  |  |  |  |  |  |  |  |  |  |  |  |
| Age (years) | 14.9 | 5.1 | 4.2 | 11.5 | 18.2 | 34.4 | 9.2 | 2.0 | 4.2 | 7.9 | 11.0 | 12.0 | 15.2 | 2.2 | 12.0 | 13.2 | 17.7 | 18.0 | 20.0 | 3.1 | 18.0 | 18.2 | 20.3 | 34.4 |
| Sex female, n (%) |  | 116 |  | 43.6 |  |  |  | 34 |  | 41.0 |  |  |  | 40 |  | 41.2 |  |  |  | 42 |  | 48.8 |  |  |
| Body heigth (m) | 1.55 | 0.18 | 1.09 | 1.43 | 1.69 | 1.94 | 1.35 | 0.12 | 1.09 | 1.27 | 1.42 | 1.79 | 1.62 | 0.10 | 1.35 | 1.54 | 1.69 | 1.82 | 1.68 | 0.11 | 1.14 | 1.61 | 1.76 | 1.94 |
| Body weight (kg) | 57.0 | 20.7 | 18.9 | 41.8 | 68.0 | 130.0 | 38.7 | 14.2 | 18.9 | 28.4 | 46.3 | 91.1 | 62.2 | 18.6 | 23.8 | 51.1 | 70.6 | 130.0 | 68.9 | 16.1 | 45.7 | 55.5 | 77.3 | 115.0 |
| BMI (m/kg^2^) | 22.9 | 5.3 | 11.3 | 19.1 | 25.2 | 45.5 | 20.8 | 4.9 | 11.3 | 16.9 | 24.3 | 38.9 | 23.7 | 5.8 | 15.3 | 20.1 | 25.3 | 45.5 | 23.9 | 4.6 | 17.2 | 20.7 | 26.0 | 40.7 |
| zBMI* (kg/m^2^) | 1.3 | 1.9 | -3.8 | 0.1 | 2.2 | 9.6 | 1.4 | 1.9 | -3.8 | 0.1 | 2.4 | 9.6 | 1.1 | 1.9 | -2.1 | -0.1 | 1.9 | 8.1 | ־ | ־ |  | ־ | ־ |  |
| Hypertension and/or HBP, n [%] |  | 30 |  | [11.3] |  |  |  | 9 |  | [10.8] |  |  |  | 13 |  | [13.4] |  |  |  | 8 |  | [9.3] |  |  |
| Diabetes, n [%] |  | 0 |  | [0] |  |  |  | 0 |  | [0] |  |  |  | 0 |  | [0] |  |  |  | 0 |  | [0] |  |  |
| Dyslipidemia, n [%] |  | 8 |  | [3.0] |  |  |  | 3 |  | [3.6] |  |  |  | 1 |  | [1.0] |  |  |  | 4 |  | [4.7] |  |  |
| Obesity, n [%] |  | 54 |  | [20.3] |  |  |  | 24 |  | [28.9] |  |  |  | 21 |  | [21.6] |  |  |  | 9 |  | [10.5] |  |  |
| Smoking, n [%] |  | 16 |  | [6.0] |  |  |  | 0 |  | [0] |  |  |  | 1 |  | [1.0] |  |  |  | 15 |  | [17.4] |  |  |
| Family history of CV disease [%] |  | 0 |  | [0] |  |  |  | 0 |  | [0] |  |  |  | 0 |  | [0] |  |  |  | 0 |  | [0] |  |  |
| Sedentary lifestyle, n [%] |  | 104 |  | [39.1] |  |  |  | 20 |  | [24.1] |  |  |  | 41 |  | [42.3] |  |  |  | 43 |  | [50.0] |  |  |
| MV: mean value. SD: standard deviation. Min: minimum value. max. Maximum value. p25th and p75th: percentile 25 and 75, respectively. BMI: body mass index. zBMI: z score of BMI *calculated only for under 18 years old. HBP: high blood pressure state during cardiovascular evaluation. CV: cardiovascular. | | | | | | | | | | | | | | | | | | | | | | | | |
|  |  |  |  |  |  |  |  |  |  |  |  |  |  |  |  |  |  |  |  |  |  |  |  |  |
