# Supplementary material for: Aortic pressure and forward and backward wave components in children, adolescents and young-adults: Agreement between brachial oscillometry, radial and carotid tonometry data and analysis of factors associated with their differences
Source: PLoS One. 2019 Dec 19;14(12):e0226709. doi: 10.1371/journal.pone.0226709 (PMC6922407; doi:10.1371/journal.pone.0226709)
Supplement: S9 Table — (DOCX) [file pone.0226709.s027.docx]

| **S9 Table. Haemodynamic and aortic wave-derived parameters measured with three different methods in the entire and age-related groups, calibrated with identical peripheral blood pressure levels obtained by oscillometry, using two different calibration schemes: pDBP/MBPc and pDBP/MBPosc** | | | | | | | | | | | | | | | |
| --- | --- | --- | --- | --- | --- | --- | --- | --- | --- | --- | --- | --- | --- | --- | --- |
|  |  |  |  |  |  |  |  |  |  |  |  |  |  |  |  |
|  |  |  |  |  |  |  |  |  |  |  |  |  |  |  |  |
|  |  |  |  |  |  |  |  |  |  |  |  |  |  |  |  |
| **Calibration: pDBP/MBPc** | | | | | | | | | | | | | | | |
| **Entire group [3 - 35 years]** | | | | | | | | | | | | | | | |
|  | **RT (Scor)** | | | | **CT (Scor)** | | | | **BOSC (MOG)** | | | | **P value** | | |
|  | **MV** | **SD** | **Min.** | **Max.** | **MV** | **SD** | **Min.** | **Max.** | **MV** | **SD** | **Min.** | **Max.** | **RT vs CT** | **RT vs OSC** | **CT vs OSC** |
| pSBP (mmHg) |  |  |  |  |  |  |  |  | 118 | 11 | 90 | 158 |  |  |  |
| pDBP (mmHg) |  |  |  |  |  |  |  |  | 65 | 9 | 44 | 104 |  |  |  |
| MBPc (Form factr: 33%) (mmHg) |  |  |  |  |  |  |  |  | 82 | 8 | 61 | 119 |  |  |  |
| HR (beats/minute) | 74 | 14 | 43 | 124 | 75 | 14 | 43 | 126 | 74 | 14 | 43 | 126 | 1.000 | 1.000 | 1.000 |
| cSBP (mmHg) | 101 | 11 | 73 | 135 | 108 | 13 | 82 | 155 | 106 | 15 | 77 | 173 | **<0.001** | **<0.001** | 0.110 |
| cPP (mmHg) | 34 | 9 | 15 | 63 | 43 | 11 | 17 | 90 | 39 | 14 | 16 | 103 | **<0.001** | **<0.001** | **0.001** |
| Pf (mmHg) | 33 | 9 | 14 | 63 | 41 | 10 | 17 | 73 | 26 | 9 | 12 | 85 | **<0.001** | **<0.001** | **<0.001** |
| Pb (mmHg) | 13 | 3 | 5 | 23 | 15 | 3 | 6 | 28 | 15 | 6 | 4 | 42 | **<0.001** | **<0.001** | 1.000 |
| **Children [3 - 12 years]** | | | | | | | | | | | | | | | |
| pSBP (mmHg) |  |  |  |  |  |  |  |  | 110 | 10 | 90 | 158 |  |  |  |
| pDBP (mmHg) |  |  |  |  |  |  |  |  | 61 | 6 | 44 | 82 |  |  |  |
| MBPc (Form factr: 33%) (mmHg) |  |  |  |  |  |  |  |  | 77 | 6 | 61 | 98 |  |  |  |
| HR (beats/minute) | 80 | 14 | 52 | 124 | 80 | 14 | 53 | 126 | 79 | 13 | 50 | 126 | 1.000 | 1.000 | 1.000 |
| cSBP (mmHg) | 92 | 8 | 73 | 119 | 101 | 10 | 82 | 140 | 94 | 9 | 77 | 123 | **<0.001** | 0.307 | **<0.001** |
| cPP (mmHg) | 30 | 7 | 15 | 53 | 40 | 9 | 21 | 80 | 32 | 8 | 16 | 60 | **<0.001** | 0.078 | **<0.001** |
| Pf (mmHg) | 28 | 7 | 14 | 53 | 38 | 7 | 23 | 56 | 22 | 6 | 13 | 44 | **<0.001** | **<0.001** | **<0.001** |
| Pb (mmHg) | 12 | 3 | 6 | 21 | 14 | 3 | 8 | 21 | 12 | 4 | 5 | 22 | 0.079 | 1.000 | **0.039** |
| **Adolescents [12-18 years]** | | | | | | | | | | | | | | | |
| pSBP (mmHg) |  |  |  |  |  |  |  |  | 120 | 11 | 96 | 147 |  |  |  |
| pDBP (mmHg) |  |  |  |  |  |  |  |  | 65 | 8 | 50 | 89 |  |  |  |
| MBPc (Form factr: 33%) (mmHg) |  |  |  |  |  |  |  |  | 83 | 7 | 68 | 106 |  |  |  |
| HR (beats/minute) | 73 | 13 | 47 | 107 | 74 | 13 | 46 | 108 | 73 | 13 | 48 | 120 | 1.000 | 1.000 | 1.000 |
| cSBP (mmHg) | 103 | 10 | 84 | 129 | 110 | 13 | 89 | 153 | 108 | 14 | 84 | 173 | **<0.001** | **0.013** | 0.733 |
| cPP (mmHg) | 36 | 9 | 17 | 63 | 45 | 12 | 18 | 90 | 41 | 14 | 17 | 103 | **<0.001** | **0.009** | 0.141 |
| Pf (mmHg) | 35 | 10 | 16 | 63 | 42 | 11 | 18 | 73 | 28 | 10 | 12 | 85 | **<0.001** | **<0.001** | **<0.001** |
| Pb (mmHg) | 13 | 3 | 5 | 23 | 15 | 3 | 6 | 25 | 16 | 7 | 4 | 42 | 0.118 | **0.001** | 0.533 |
| **Young adults [18-35 years]** | | | | | | | | | | | | | | | |
| pSBP (mmHg) |  |  |  |  |  |  |  |  | 124 | 9 | 105 | 154 |  |  |  |
| pDBP (mmHg) |  |  |  |  |  |  |  |  | 70 | 10 | 44 | 104 |  |  |  |
| MBPc (Form factr: 33%) (mmHg) |  |  |  |  |  |  |  |  | 87 | 9 | 64 | 119 |  |  |  |
| HR (beats/minute) | 69 | 12 | 43 | 105 | 70 | 12 | 43 | 104 | 69 | 13 | 43 | 112 | 1.000 | 1.000 | 1.000 |
| cSBP (mmHg) | 107 | 9 | 89 | 135 | 114 | 11 | 92 | 155 | 115 | 13 | 94 | 162 | **0.001** | **<0.001** | 1.000 |
| cPP (mmHg) | 36 | 8 | 16 | 55 | 44 | 11 | 17 | 72 | 44 | 15 | 19 | 90 | **<0.001** | **<0.001** | 1.000 |
| Pf (mmHg) | 35 | 9 | 15 | 55 | 42 | 11 | 17 | 71 | 29 | 10 | 13 | 69 | **<0.001** | **0.001** | **<0.001** |
| Pb (mmHg) | 14 | 3 | 8 | 21 | 16 | 4 | 8 | 28 | 18 | 7 | 6 | 41 | **0.012** | **<0.001** | 0.442 |
| **Calibration: pDBP/MBPosc** | | | | | | | | | | | | | | | |
| **Entire group [3 - 35 years]** | | | | | | | | | | | | | | | |
|  | **Radial Tonometry (Scor)** | | | | **Carotid Tonometry (Scor)** | | | | **Brachial Oscillometry (MOG)** | | | | **P value** | | |
|  | **MV** | **SD** | **Min** | **Max** | **MV** | **SD** | **Min** | **Max** | **MV** | **SD** | **Min** | **Max** | **RT vs CT** | **RT vs OSC** | **CT vs OSC** |
| pSBP (mmHg) |  |  |  |  |  |  |  |  | 118 | 11 | 90 | 158 |  |  |  |
| pDBP (mmHg) |  |  |  |  |  |  |  |  | 65 | 9 | 44 | 104 |  |  |  |
| MBPosc (mmHg) |  |  |  |  |  |  |  |  | 89 | 9 | 68 | 125 |  |  |  |
| HR (beats/minute) | 74 | 14 | 43 | 124 | 74 | 14 | 43 | 126 | 74 | 14 | 43 | 126 | 1.000 | 1.000 | 1.000 |
| cSBP (mmHg) | 115 | 13 | 81 | 156 | 125 | 16 | 93 | 187 | 122 | 19 | 87 | 213 | **<0.001** | **<0.001** | 0.072 |
| cPP (mmHg) | 48 | 12 | 22 | 86 | 60 | 16 | 24 | 123 | 55 | 18 | 22 | 135 | **<0.001** | **<0.001** | **0.002** |
| Pf (mmHg) | 45 | 12 | 20 | 86 | 56 | 14 | 24 | 102 | 37 | 12 | 15 | 112 | **<0.001** | **<0.001** | **<0.001** |
| Pb (mmHg) | 18 | 4 | 7 | 32 | 21 | 5 | 10 | 37 | 22 | 8 | 7 | 56 | **<0.001** | **<0.001** | 1.000 |
| **Children [3 - 12 years]** | | | | | | | | | | | | | | | |
| pSBP (mmHg) |  |  |  |  |  |  |  |  | 110 | 10 | 90 | 158 |  |  |  |
| pDBP (mmHg) |  |  |  |  |  |  |  |  | 61 | 6 | 44 | 82 |  |  |  |
| MBPc (Form factr: 33%) (mmHg) |  |  |  |  |  |  |  |  | 84 | 6 | 68 | 105 |  |  |  |
| HR (beats/minute) | 80 | 14 | 52 | 124 | 81 | 14 | 53 | 126 | 79 | 13 | 50 | 126 | 1.000 | 1.000 | 1.000 |
| cSBP (mmHg) | 104 | 10 | 81 | 143 | 117 | 13 | 93 | 172 | 108 | 12 | 87 | 149 | **<0.001** | 0.137 | **<0.001** |
| cPP (mmHg) | 41 | 9 | 22 | 75 | 56 | 13 | 29 | 112 | 46 | 11 | 22 | 86 | **<0.001** | **0.024** | **<0.001** |
| Pf (mmHg) | 39 | 9 | 20 | 75 | 54 | 10 | 34 | 78 | 31 | 8 | 19 | 62 | **<0.001** | **<0.001** | **<0.001** |
| Pb (mmHg) | 17 | 4 | 8 | 29 | 18 | 3 | 11 | 25 | 17 | 5 | 7 | 32 | 0.208 | 1.000 | **0.432** |
| **Adolescents [12-18 years]** | | | | | | | | | | | | | | | |
| pSBP (mmHg) |  |  |  |  |  |  |  |  | 120 | 11 | 96 | 147 |  |  |  |
| pDBP (mmHg) |  |  |  |  |  |  |  |  | 65 | 8 | 50 | 89 |  |  |  |
| MBPc (Form factr: 33%) (mmHg) |  |  |  |  |  |  |  |  | 90 | 8 | 74 | 113 |  |  |  |
| HR (beats/minute) | 73 | 13 | 47 | 107 | 73 | 13 | 46 | 108 | 73 | 13 | 48 | 120 | 1.000 | 1.000 | 1.000 |
| cSBP (mmHg) | 117 | 13 | 96 | 146 | 127 | 17 | 95 | 179 | 125 | 18 | 95 | 213 | **<0.001** | **0.004** | 0.972 |
| cPP (mmHg) | 50 | 13 | 23 | 86 | 62 | 58 | 24 | 123 | 58 | 18 | 25 | 135 | **<0.001** | **0.003** | 0.219 |
| Pf (mmHg) | 48 | 13 | 21 | 86 | 57 | 15 | 24 | 100 | 39 | 13 | 15 | 112 | **<0.001** | **<0.001** | **<0.001** |
| Pb (mmHg) | 19 | 4 | 7 | 32 | 21 | 5 | 10 | 35 | 23 | 9 | 7 | 56 | 0.089 | **<0.001** | 0.458 |
| **Young adults [18-35 years]** | | | | | | | | | | | | | | | |
| pSBP (mmHg) |  |  |  |  |  |  |  |  | 124 | 9 | 105 | 154 |  |  |  |
| pDBP (mmHg) |  |  |  |  |  |  |  |  | 70 | 10 | 44 | 104 |  |  |  |
| MBPc (Form factr: 33%) (mmHg) |  |  |  |  |  |  |  |  | 94 | 9 | 72 | 125 |  |  |  |
| HR (beats/minute) | 69 | 12 | 43 | 105 | 70 | 12 | 43 | 104 | 69 | 13 | 43 | 112 | 1.000 | 1.000 | 1.000 |
| cSBP (mmHg) | 122 | 10 | 102 | 156 | 131 | 14 | 102 | 187 | 133 | 17 | 106 | 198 | **<0.001** | **<0.001** | 1.000 |
| cPP (mmHg) | 51 | 11 | 23 | 78 | 61 | 15 | 24 | 104 | 62 | 19 | 28 | 120 | **<0.001** | **<0.001** | 1.000 |
| Pf (mmHg) | 48 | 12 | 22 | 78 | 57 | 14 | 24 | 102 | 40 | 13 | 19 | 91 | **<0.001** | **0.001** | **<0.001** |
| Pb (mmHg) | 19 | 4 | 11 | 29 | 23 | 5 | 12 | 37 | 25 | 9 | 9 | 55 | **0.004** | **<0.001** | 0.395 |
| MV: mean value. SD: standard deviation. RT: radial applanation tonometry record, obtained with SphygmoCor device. CT: carotid applanation tonometry record, obtained with SphygmoCor device. BOSC: brachial oscillometry/plethysmography record, obtained with Mobil-O-Graph device. Min. and Max.: minimum and maximal value, respectively. pSBP, pDBP, MBPc: peripheral (brachial) systolic, diastolic and mean (calculated) blood pressure respectively. HR: heart rate. cSBP, cPP: central systolic and pulse blood pressure, respectively. Pf: forward wave height (amplitude). Pb: backward wave height (amplitude). p value was obtained from ANOVA plus Bonferroni post-hoc test. Significance level: p<0.05 (red text). | | | | | | | | | | | | | | | |
|  |  |  |  |  |  |  |  |  |  |  |  |  |  |  |  |
|  |  |  |  |  |  |  |  |  |  |  |  |  |  |  |  |
|  |  |  |  |  |  |  |  |  |  |  |  |  |  |  |  |
|  |  |  |  |  |  |  |  |  |  |  |  |  |  |  |  |
