# Supplementary material for: Aortic pressure and forward and backward wave components in children, adolescents and young-adults: Agreement between brachial oscillometry, radial and carotid tonometry data and analysis of factors associated with their differences
Source: PLoS One. 2019 Dec 19;14(12):e0226709. doi: 10.1371/journal.pone.0226709 (PMC6922407; doi:10.1371/journal.pone.0226709)
Supplement: S10 Table — (DOCX) [file pone.0226709.s028.docx]

| **S10 Table. cSBP and cPP: agreement among parameters measured with three different methods in the entire and age-related groups, calibrated with identical peripheral blood pressure levels obtained by oscillometry, using two different calibration schemes: pDBP/MBPc and pDBP/MBPosc [Summary table]** | | | | | | | | | | | | |
| --- | --- | --- | --- | --- | --- | --- | --- | --- | --- | --- | --- | --- |
|  |  |  |  |  |  |  |  |  |  |  |  |  |
|  |  |  |  |  |  |  |  |  |  |  |  |  |
| **cSBP** | **Entire group [3-35 years]** | | | **Children [3-12 years]** | | | **Adolescents [12-18 years]** | | | **Young adults [18-35 years]** | | |
|  | RT- CT | RT -BOSC | CT - BOSC | RT- CT | RT -BOSC | CT - BOSC | RT- CT | RT - BOSC | CT - BOSC | RT- CT | RT - BOSC | CT - BOSC |
| **Calibration: pDBP/MBPc** |  |  |  |  |  |  |  |  |  |  |  |  |
| r | 0.89 | 0.78 | 0.71 | 0.83 | 0.81 | 0.71 | 0.88 | 0.65 | 0.61 | 0.87 | 0.61 | 0.59 |
| p | **<0.001** | **<0.001** | **<0.001** | **<0.001** | **<0.001** | **<0.001** | **<0.001** | **<0.001** | **<0.001** | **<0.001** | **<0.001** | **<0.001** |
| Mean error (mmHg) | -7.2 | -4.5 | 2.8 | -7.9 | -2.1 | 6.0 | -7.5 | -4.5 | 3.1 | -6.2 | -7.0 | -0.9 |
| Mean error, CI 95% Upper Limit (mmHg) | -6.5 | -3.5 | 4.0 | -6.8 | -1.0 | 7.6 | -6.2 | -2.5 | 5.3 | -5.0 | -4.9 | 1.4 |
| Mean error, CI 95% Lower Limit (mmHg) | -7.9 | -5.5 | 1.6 | -9.1 | -3.3 | 4.5 | -8.8 | -6.4 | 0.9 | -7.4 | -9.0 | -3.2 |
| Mean error, p value | **<0.001** | **<0.001** | **<0.001** | **<0.001** | **<0.001** | **<0.001** | **<0.001** | **<0.001** | **0.006** | **<0.001** | **<0.001** | 0.425 |
| Mean error, SD (mmHg) | 5.7 | 8.5 | 9.9 | 5.1 | 5.1 | 7.0 | 6.3 | 9.5 | 10.8 | 5.5 | 16.3 | 10.2 |
| CI 95%, Upper limit (mmHg) | 4.0 | 12.1 | 22.1 | 2.2 | 7.9 | 19.7 | 4.9 | 14.2 | 24.3 | 4.5 | 11.1 | 19.0 |
| CI 95%, Lower limit (mmHg) | -18.4 | -21.1 | -16.6 | -18.0 | -12.2 | -7.7 | -19.9 | -23.1 | -18.0 | -16.9 | -25.1 | -20.9 |
| Regression equation | y= 9.0 - 0.2x | y= 21.2 - 0.2x | y= 11.1 - 0.08x | y=12.8 - 0.2x | y= 11.5 - 0.1x | y=-4.1 + 0.1x | y= 19.9 - 0.3x | y= 17.8 - 0.2x | y= -12.9 + 0.1x | y= 26.6 - 0.3x | y= 37.2 - 0.4x | y= 7.5 - 0.07x |
| p (Slope) | **<0.001** | **<0.001** | 0.133 | **0.002** | **0.048** | 0.261 | **<0.001** | **0.027** | 0.151 | **<0.001** | **<0.001** | 0.528 |
| **Calibration: pDBP/MBPosc** | RT- CT | RT -BOSC | CT - BOSC | RT- CT | RT -BOSC | CT - BOSC | RT- CT | RT - BOSC | CT - BOSC | RT- CT | RT - BOSC | CT - BOSC |
| r | 0.83 | 0.77 | 0.66 | 0.75 | 0.78 | 0.67 | 0.83 | 0.65 | 0.57 | 0.83 | 0.60 | 0.56 |
| p | **<0.001** | **<0.001** | **<0.001** | **<0.001** | **<0.001** | **<0.001** | **<0.001** | **<0.001** | **<0.001** | **<0.001** | **<0.001** | **<0.001** |
| Mean error (mmHg) | -10.5 | -6.6 | 3.7 | -12.1 | -3.6 | 8.5 | -10.4 | -6.6 | 3.5 | -9.0 | -9.8 | -0.9 |
| Mean error, CI 95% Upper Limit (mmHg) | -9.4 | -5.3 | 5.4 | -10.2 | -2.0 | 10.7 | -8.4 | -4.1 | 6.6 | -7.2 | -7.2 | 2.2 |
| Mean error, CI 95% Lower Limit (mmHg) | -11.5 | -7.9 | 2.0 | -13.9 | -5.1 | 6.4 | -12.3 | -9.1 | 0.4 | -10.9 | -12.5 | -4.0 |
| Mean error, p value | **<0.001** | **<0.001** | **<0.001** | **<0.001** | **<0.001** | **<0.001** | **<0.001** | **<0.001** | **0.029** | **<0.001** | **<0.001** | 0.555 |
| Mean error, SD (mmHg) | 8.9 | 11.1 | 13.8 | 8.3 | 7.0 | 9.8 | 9.6 | 12.3 | 15.3 | 8.4 | 12.1 | 13.9 |
| CI 95%, Upper limit (mmHg) | 6.9 | 15.1 | 30.7 | 4.3 | 10.4 | 27.7 | 8.4 | 17.6 | 33.6 | 7.4 | 13.8 | 26.3 |
| CI 95%, Lower limit (mmHg) | -27.9 | -28.3 | -23.3 | -28.5 | -17.3 | -10.7 | -29.1 | -30.7 | -26.6 | -25.5 | -33.4 | -28.1 |
| Regression equation | y= 15.8 - 0.2x | y= 28.7 - 0.3x | y= 15.5 - 0.1x | y= 20.5 + 0.3x | y= 14.8 - 0.2x | y=-6.4 + 0.1x | y= 29.2 - 0.3x | y= 25.3 - 0.3x | y= -4.5 + 0.06x | y= 44.8 - 0.4x | y= 56.2 - 0.5x | y= 6.5 - 0.06x |
| p (Slope) | **<0.001** | **<0.001** | 0.090 | **0.001** | **0.030** | 0.186 | **<0.001** | **0.006** | 0.562 | **<0.001** | **<0.001** | 0.635 |
| **cPP** | **Entire group [3 - 35 years]** | | | **Children [3 - 12 years]** | | | **Adolescents [12 - 18 years]** | | | **Young adults [18 - 35 years]** | | |
|  | RT- CT | RT -BOSC | CT - BOSC | RT- CT | RT -BOSC | CT - BOSC | RT- CT | RT - BOSC | CT - BOSC | RT- CT | RT - BOSC | CT - BOSC |
| **Calibration: pDBP/MBPc** |  |  |  |  |  |  |  |  |  |  |  |  |
| r | 0.83 | 0.77 | 0.64 | 0.73 | 0.80 | 0.59 | 0.84 | 0.68 | 0.59 | 0.85 | 0.79 | 0.69 |
| p | **<0.001** | **<0.001** | **<0.001** | **<0.001** | **<0.001** | **<0.001** | **<0.001** | **<0.001** | **<0.001** | **<0.001** | **<0.001** | **<0.001** |
| Mean error (mmHg) | -8.5 | -4.7 | 3.8 | -9.3 | -2.5 | 7.0 | -8.8 | -4.4 | 4.3 | -7.3 | -7.1 | 0.1 |
| Mean error, CI 95% Upper Limit (mmHg) | -7.7 | -3.7 | 5.1 | -8.1 | -1.4 | 8.6 | -7.5 | -2.7 | 6.5 | -6.0 | -5.1 | 2.4 |
| Mean error, CI 95% Lower Limit (mmHg) | -9.2 | -5.6 | 2.6 | -10.5 | -3.6 | 5.5 | -10.2 | -6.2 | 2.1 | -8.6 | -9.1 | -2.2 |
| Mean error, p value | **<0.001** | **<0.001** | **<0.001** | **<0.001** | **<0.001** | **<0.001** | **<0.001** | **<0.001** | **<0.001** | **<0.001** | **<0.001** | 0.957 |
| Mean error, SD (mmHg) | 6.0 | 7.9 | 10.0 | 5.2 | 4.9 | 6.9 | 6.6 | 8.4 | 10.8 | 5.8 | 9.1 | 10.4 |
| CI 95%, Upper limit (mmHg) | 3.3 | 10.9 | 23.2 | 0.9 | 7.1 | 20.6 | 4.2 | 12.1 | 25.5 | 4.1 | 10.8 | 20.4 |
| CI 95%, Lower limit (mmHg) | -20.2 | -20.2 | -15.7 | -19.5 | -12.2 | -6.5 | -21.8 | -21.0 | -16.9 | -18.7 | -25.0 | -20.3 |
| Regression equation | y= 0.9 - 0.2x | y= 10.0 - 0.4x | y= 10.1 - 0.2x | y= -2.0 - 0.2x | y= 4.4 - 0.2x | y= 6.2 + 0.02x | y= 3.7 - 0.3x | y= 6.0- 0.3x | y= -0.06 + 0.1x | y= 5.0 - 0.3x | y= 15.9 - 0.6x | y= 13.6 - 0.3x |
| p (Slope) | **<0.001** | **<0.001** | **0.009** | **0.018** | **0.004** | 0.843 | **<0.001** | **0.003** | 0.333 | **<0.001** | **<0.001** | **0.002** |
| **Calibration: pDBP/MBPosc** | RT- CT | RT -BOSC | CT - BOSC | RT- CT | RT -BOSC | CT - BOSC | RT- CT | RT - BOSC | CT - BOSC | RT- CT | RT - BOSC | CT - BOSC |
| r | 0.80 | 0.80 | 0.66 | 0.72 | 0.80 | 0.69 | 0.81 | 0.73 | 0.61 | 0.85 | 0.80 | 0.70 |
| p | **<0.001** | **<0.001** | **<0.001** | **<0.001** | **<0.001** | **<0.001** | **<0.001** | **<0.001** | **<0.001** | **<0.001** | **<0.001** | **<0.001** |
| Mean error (mmHg) | -12.2 | -7.1 | 5.0 | -14.3 | -4.4 | 9.8 | -12.3 | -7.1 | 5.1 | -10.2 | -10.0 | 0.1 |
| Mean error, CI 95% Upper Limit (mmHg) | -11.1 | -5.9 | 6.7 | -12.3 | -2.9 | 11.9 | -10.3 | -4.8 | 8.2 | -8.4 | -7.5 | 3.1 |
| Mean error, CI 95% Lower Limit (mmHg) | -13.4 | -8.4 | 3.3 | -16.2 | -5.9 | 7.7 | -14.4 | -9.3 | 2.1 | -12.0 | -12.5 | -2.9 |
| Mean error, p value | **<0.001** | **<0.001** | **<0.001** | **<0.001** | **<0.001** | **<0.001** | **<0.001** | **<0.001** | **0.001** | **<0.001** | **<0.001** | 0.947 |
| Mean error, SD (mmHg) | 9.3 | 10.3 | 13.4 | 8.9 | 6.8 | 9.6 | 9.6 | 9.7 | 14.9 | 9.3 | 7.6 | 11.5 |
| CI 95%, Upper limit (mmHg) | 5.9 | 13.0 | 31.3 | 3.2 | 8.9 | 28.6 | 7.4 | 14.7 | 34.3 | 5.9 | 12.4 | 26.4 |
| CI 95%, Lower limit (mmHg) | -30.4 | -27.2 | -21.4 | -31.7 | -17.8 | -9.0 | -32.1 | -28.9 | -24.1 | -26.3 | -32.4 | -26.2 |
| Regression equation | y= 2.9 - 0.3x | y= 12.2 - 0.4x | y= 11.4 - 0.1x | y= 2.4 - 0.3x | y= 4.4 - 0.2x | y= 2.2 + 0.1x | y= 7.2 - 0.3x | y= 9.7 - 0.3x | y= 2.6 + 0.04x | y= 7.6 - 0.3x | y= 19.6 - 0.5x | y= 14.6 - 0.2x |
| p (Slope) | **<0.001** | **<0.001** | 0.051 | **<0.001** | **0.008** | 0.126 | **<0.001** | **<0.001** | 0.689 | **<0.001** | **<0.001** | **0.001** |
| RT: radial applanation tonometry record, obtained with SphygmoCor device (SCOR). CT: carotid applanation tonometry record, obtained with SCOR. BOSC: brachial oscillometry/plethysmography record, obtained with Mobil-O-Graph device (MOG). cSBP, cPP: central systolic and pulse blood pressure, respectively. r: correlation (Pearson) coefficient. β: slope of regression equation. Significance level: p value <0.05 (red text). Bland-Altman analysis: variable "x" was considered the mean of both methods compared (eg. (RT+CT)/2) and variable "y" the difference among first and second method (eg. RT minus CT). MBPc: mean blood pressure calculated as pDBP+((pSBP-pDBP)/3). MBPosc: mean blood pressure measured by oscillometry. CI: confidence interval. | | | | | | | | | | | | |
|  |  |  |  |  |  |  |  |  |  |  |  |  |
|  |  |  |  |  |  |  |  |  |  |  |  |  |
|  |  |  |  |  |  |  |  |  |  |  |  |  |
