# Supplementary material for: Aortic pressure and forward and backward wave components in children, adolescents and young-adults: Agreement between brachial oscillometry, radial and carotid tonometry data and analysis of factors associated with their differences
Source: PLoS One. 2019 Dec 19;14(12):e0226709. doi: 10.1371/journal.pone.0226709 (PMC6922407; doi:10.1371/journal.pone.0226709)
Supplement: S11 Table — (DOCX) [file pone.0226709.s029.docx]

| **S11 Table. Pf and Pb: agreement among parameters measured with three different methods in the entire and age-related groups, calibrated with identical peripheral blood pressure levels obtained by oscillometry, using two different calibration schemes: pDBP/MBPc and pDBP/MBPosc [Summary table]** | | | | | | | | | | | | |
| --- | --- | --- | --- | --- | --- | --- | --- | --- | --- | --- | --- | --- |
|  |  |  |  |  |  |  |  |  |  |  |  |  |
|  |  |  |  |  |  |  |  |  |  |  |  |  |
| **Pf** | **Entire group [3-35 years]** | | | **Children [3-12 years]** | | | **Adolescents [12-18 years]** | | | **Young adults [18-35 years]** | | |
|  | RT- CT | RT -BOSC | CT - BOSC | RT- CT | RT -BOSC | CT - BOSC | RT- CT | RT - BOSC | CT - BOSC | RT- CT | RT - BOSC | CT - BOSC |
| **Calibration: pDBP/MBPc** |  |  |  |  |  |  |  |  |  |  |  |  |
| r | 0.79 | 0.75 | 0.66 | 0.70 | 0.75 | 0.54 | 0.79 | 0.68 | 0.70 | 0.82 | 0.77 | 0.64 |
| p | **<0.001** | **<0.001** | **<0.001** | **<0.001** | **<0.001** | **<0.001** | **<0.001** | **<0.001** | **<0.001** | **<0.001** | **<0.001** | **<0.001** |
| Mean error (mmHg) | -8.1 | 6.6 | 14.2 | -9.6 | 6.4 | 16.0 | -7.7 | 7.1 | 13.6 | -7.4 | 6.3 | 13.7 |
| Mean error, CI 95% Upper Limit (mmHg) | -7.2 | 7.4 | 15.3 | -8.0 | 7.4 | 17.9 | -6.1 | 8.7 | 15.4 | -5.9 | 7.6 | 15.8 |
| Mean error, CI 95% Lower Limit (mmHg) | -9.0 | 5.8 | 13.1 | -11.2 | 5.4 | 14.2 | -9.2 | 5.6 | 11.9 | -9.0 | 4.9 | 11.6 |
| Mean error, p value | **<0.001** | **<0.001** | **<0.001** | **<0.001** | **<0.001** | **<0.001** | **<0.001** | **<0.001** | **<0.001** | **<0.001** | **<0.001** | **<0.001** |
| Mean error, SD (mmHg) | 6.0 | 6.0 | 7.5 | 5.4 | 4.5 | 6.3 | 6.4 | 7.3 | 4.9 | 5.9 | 5.9 | 8.4 |
| CI 95%, Upper limit (mmHg) | 3.7 | 18.4 | 28.9 | 1.0 | 15.2 | 28.4 | 4.8 | 21.4 | 27.9 | 4.2 | 17.8 | 30.1 |
| CI 95%, Lower limit (mmHg) | -19.9 | -5.2 | -0.5 | -20.3 | -2.4 | 3.6 | -20.2 | -7.1 | -0.6 | -19.0 | -5.3 | -2.7 |
| Regression equation | y= -1.9 - 0.2x | y= 3.0 + 0.1x | y= 7.1 + 0.2x | y= -1.4 - 0.2x | y= 0.9 + 0.2x | y= -4.1 + 0.7x | y= 0.8 - 0.2x | y= 0.2 + 0.2x | y= 5.2 + 0.2x | y= -0.9 - 0.2x | y= 7.3 - 0.03x | y= 8.0 + 0.2x |
| p (Slope) | **0.049** | **0.014** | **0.002** | 0.051 | **0.011** | **<0.001** | **0.008** | **0.017** | **0.017** | **0.049** | 0.708 | 0.172 |
| **Calibration: pDBP/MBPosc** | RT- CT | RT -BOSC | CT - BOSC | RT- CT | RT -BOSC | CT - BOSC | RT- CT | RT - BOSC | CT - BOSC | RT- CT | RT - BOSC | CT - BOSC |
| r | 0.75 | 0.76 | 0.62 | 0.66 | 0.75 | 0.46 | 0.79 | 0.69 | 0.68 | 0.73 | 0.77 | 0.59 |
| p | **<0.001** | **<0.001** | **<0.001** | **<0.001** | **<0.001** | **<0.001** | **<0.001** | **<0.001** | **<0.001** | **<0.001** | **<0.001** | **<0.001** |
| Mean error (mmHg) | -11.4 | 8.9 | 19.7 | -13.6 | 8.5 | 22.0 | -11.2 | 9.4 | 19.7 | -9.9 | 8.8 | 18.2 |
| Mean error, CI 95% Upper Limit (mmHg) | -9.9 | 9.9 | 21.3 | -11.2 | 9.9 | 24.7 | -8.8 | 11.4 | 22.4 | -7.3 | 10.6 | 21.2 |
| Mean error, CI 95% Lower Limit (mmHg) | -12.8 | 7.9 | 18.1 | -15.9 | 7.1 | 19.4 | -13.6 | 7.4 | 17.0 | -12.4 | 7.0 | 15.3 |
| Mean error, p value | **<0.001** | **<0.001** | **<0.001** | **<0.001** | **<0.001** | **<0.001** | **<0.001** | **<0.001** | **<0.001** | **<0.001** | **<0.001** | **<0.001** |
| Mean error, SD (mmHg) | 9.1 | 8.0 | 10.8 | 7.7 | 6.3 | 8.7 | 9.6 | 9.7 | 11.2 | 9.3 | 7.6 | 11.5 |
| CI 95%, Upper limit (mmHg) | 6.5 | 24.6 | 40.8 | 1.6 | 20.8 | 39.1 | 7.7 | 28.3 | 41.6 | 8.4 | 23.6 | 40.7 |
| CI 95%, Lower limit (mmHg) | -29.2 | -6.8 | -1.4 | -28.7 | -3.9 | 4.9 | -30.1 | -9.6 | -2.3 | -28.1 | -6.1 | -4.3 |
| Regression equation | y= -0.5 -0.2x | y= 2.3 + 0.2x | y= 3.3 + 0.4x | y= -1.7 - 0.3x | y= 0.3 + 0.2x | y= -6.1 + 0.7x | y= 4.9 - 0.3x | y= -0.5 + 0.2x | y= -2.5 + 0.5x | y= -1.3 - 0.2x | y= 5.8 + 0.07x | y= 6.3 + 0.2x |
| p (Slope) | **<0.001** | **<0.001** | **<0.001** | 0.068 | **0.007** | **<0.001** | **0.001** | **0.013** | **<0.001** | 0.131 | 0.074 | 0.063 |
| **Pb** | **Entire group [3 - 35 years]** | | | **Children [3 - 12 years]** | | | **Adolescents [12 - 18 years]** | | | **Young adults [18 - 35 years]** | | |
|  | RT- CT | RT -BOSC | CT - BOSC | RT- CT | RT -BOSC | CT - BOSC | RT- CT | RT - BOSC | CT - BOSC | RT- CT | RT - BOSC | CT - BOSC |
| **Calibration: pDBP/MBPc** |  |  |  |  |  |  |  |  |  |  |  |  |
| r | 0.83 | 0.61 | 0.60 | 0.78 | 0.71 | 0.54 | 0.84 | 0.56 | 0.62 | 0.86 | 0.58 | 0.53 |
| p | **<0.001** | **<0.001** | **<0.001** | **<0.001** | **<0.001** | **<0.001** | **<0.001** | **<0.001** | **<0.001** | **<0.001** | **<0.001** | **<0.001** |
| Mean error (mmHg) | -1.8 | -1.9 | -0.7 | -0.9 | 0.0 | 0.6 | -1.8 | -2.6 | -1.1 | -2.6 | -3.3 | -1.2 |
| Mean error, CI 95% Upper Limit (mmHg) | -1.5 | -1.4 | 0.0 | -0.3 | 0.5 | 1.5 | -1.4 | -1.5 | 0.0 | -2.1 | -2.2 | 0.1 |
| Mean error, CI 95% Lower Limit (mmHg) | -2.1 | -2.5 | -1.4 | -1.4 | -0.6 | -0.2 | -2.3 | -3.6 | -2.3 | -3.1 | -4.5 | -2.6 |
| Mean error, p value | **<0.001** | **<0.001** | 0.052 | **0.002** | 0.940 | 0.150 | **<0.001** | **<0.001** | 0.060 | **<0.001** | **<0.001** | 0.066 |
| Mean error, SD (mmHg) | 2.0 | 4.5 | 4.7 | 1.9 | 2.5 | 3.0 | 1.8 | 5.0 | 4.9 | 2.0 | 5.0 | 5.4 |
| CI 95%, Upper limit (mmHg) | 2.0 | 6.9 | 8.6 | 2.8 | 4.9 | 6.5 | 1.7 | 7.2 | 8.5 | 1.3 | 6.5 | 9.3 |
| CI 95%, Lower limit (mmHg) | -5.7 | -10.8 | -9.9 | -4.5 | -5.0 | -5.2 | -5.3 | -12.3 | -10.7 | -6.5 | -13.1 | -11.8 |
| Regression equation | y= 0.7 - 0.2x | y= 8.6 - 0.7x | y= 9.7 - 0.7x | y= -0.8 - 0.002x | y= 2.7 - 0.2x | y= 3.4 -0.2x | y= -0.9 - 0.07x | y= 9.0 - 0.8x | y= 11.0 - 0.8x | y= 1.5- 0.3x | y= 10.6 - 0.9x | y= 11.0 - 0.7x |
| p (Slope) | **<0.001** | **<0.001** | **<0.001** | 0.987 | **0.017** | 0.203 | 0.337 | **<0.0001** | **<0.0001** | **0.001** | **<0.0001** | **<0.0001** |
| **Calibration: pDBP/MBPosc** | RT- CT | RT -BOSC | CT - BOSC | RT- CT | RT -BOSC | CT - BOSC | RT- CT | RT - BOSC | CT - BOSC | RT- CT | RT - BOSC | CT - BOSC |
| r | 0.83 | 0.61 | 0.60 | 0.63 | 0.74 | 0.51 | 0.80 | 0.62 | 0.64 | 0.86 | 0.55 | 0.57 |
| p | **<0.001** | **<0.001** | **<0.001** | **<0.001** | **<0.001** | **<0.001** | **<0.001** | **<0.001** | **<0.001** | **<0.001** | **<0.001** | **<0.001** |
| Mean error (mmHg) | -2.5 | -3.0 | -0.9 | -0.8 | -0.5 | 0.1 | -2.8 | -3.7 | -1.4 | -3.6 | -5.0 | -1.3 |
| Mean error, CI 95% Upper Limit (mmHg) | -2.0 | -2.2 | -0.1 | 0.1 | 0.2 | 1.3 | -2.0 | -2.4 | 0.1 | -2.8 | -3.3 | 0.3 |
| Mean error, CI 95% Lower Limit (mmHg) | -3.0 | -3.7 | -1.8 | -1.7 | -1.3 | -1.1 | -3.5 | -5.1 | -2.9 | -4.3 | -6.6 | -3.0 |
| Mean error, p value | **<0.001** | **<0.001** | **0.033** | 0.066 | 0.151 | 0.886 | **<0.001** | **<0.001** | 0.065 | **<0.001** | **<0.001** | 0.117 |
| Mean error, SD (mmHg) | 3.0 | 6.0 | 5.8 | 3.0 | 3.3 | 3.9 | 2.8 | 6.4 | 6.2 | 2.8 | 6.9 | 6.5 |
| CI 95%, Upper limit (mmHg) | 3.5 | 8.8 | 10.4 | 5.0 | 6.0 | 7.8 | 2.8 | 8.9 | 10.7 | 1.9 | 8.5 | 11.4 |
| CI 95%, Lower limit (mmHg) | -8.5 | -14.7 | -12.3 | -6.7 | -7.1 | -7.6 | -8.3 | -16.3 | -13.5 | -9.0 | -18.4 | -14.0 |
| Regression equation | y= 1.8 - 0.2x | y= 10.9 - 0.7x | y= 10.9 - 0.5x | y= -0.4 - 0.02x | y= 2.6 - 0.2x | y= 5.1 -0.3x | y= -0.8 - 0.1x | y= 11.4 - 0.7x | y= 12.8 - 0.7x | y= 2.3- 0.3x | y= 14.5 - 0.9x | y= 12.3 - 0.6x |
| p (Slope) | **<0.001** | **<0.001** | **<0.001** | 0.869 | **0.037** | 0.118 | 0.240 | **<0.001** | **<0.001** | **0.001** | **<0.001** | **<0.001** |
| RT: radial applanation tonometry record, obtained with SphygmoCor device (SCOR). CT: carotid applanation tonometry record, obtained with SCOR. BOSC: brachial oscillometry/plethysmography record, obtained with Mobil-O-Graph device (MOG). Pf: forward wave height (amplitude). Pb: backward wave height (amplitude). r: correlation (Pearson) coefficient. β: slope of regression equation. Significance level: p value <0.05 (red text). Bland-Altman analysis: variable "x" was considered the mean of both methods compared (eg. (RT+CT)/2) and variable "y" the difference among first and second method (eg. RT minus CT). MBPc: mean blood pressure calculated as pDBP+((pSBP-pDBP)/3). MBPosc: mean blood pressure measured by oscillometry. CI: confidence interval. | | | | | | | | | | | | |
|  |  |  |  |  |  |  |  |  |  |  |  |  |
|  |  |  |  |  |  |  |  |  |  |  |  |  |
|  |  |  |  |  |  |  |  |  |  |  |  |  |
