# Supplementary material for: Aortic pressure and forward and backward wave components in children, adolescents and young-adults: Agreement between brachial oscillometry, radial and carotid tonometry data and analysis of factors associated with their differences
Source: PLoS One. 2019 Dec 19;14(12):e0226709. doi: 10.1371/journal.pone.0226709 (PMC6922407; doi:10.1371/journal.pone.0226709)
Supplement: S12 Table — (DOCX) [file pone.0226709.s030.docx]

| **S12 Table. cSBP: agreement among parameters measured with three different methods in the entire and age-related groups, calibrated with identical peripheral blood pressure levels obtained by oscillometry (Calibration scheme: pDBP/MBPc) [Extended table]** | | | | | | | | | | | | | |
| --- | --- | --- | --- | --- | --- | --- | --- | --- | --- | --- | --- | --- | --- |
|  |  |  |  |  |  |  |  |  |  |  |  |  |  |
|  |  |  |  |  |  |  |  |  |  |  |  |  |  |
| **cSBP** | | **Entire group [3-35 years]** | | | **Children [3-12 years]** | | | **Adolescents [12-18 years]** | | | **Young adults [18-35 years]** | | |
|  |  | **RT (SCOR)** | **CT (SCOR)** | **BOSC (MOG)** | **RT (SCOR)** | **CT (SCOR)** | **BOSC (MOG)** | **RT (SCOR)** | **CT (SCOR)** | **BOSC (MOG)** | **RT (SCOR)** | **CT (SCOR)** | **BOSC (MOG)** |
| **Radial tonometry (SCOR)** | r | ˗ | 0.89 | 0.78 | ˗ | 0.83 | 0.81 | ˗ | 0.88 | 0.65 | ˗ | 0.87 | 0.61 |
|  | p | ˗ | **<0.001** | **<0.001** | ˗ | **<0.001** | **<0.001** | ˗ | **<0.001** | **<0.001** | ˗ | **<0.001** | **<0.001** |
|  | Mean error (mmHg) | ˗ | -7.21 | -4.52 | ˗ | -7.94 | -2.14 | ˗ | -7.50 | -4.45 | ˗ | -6.21 | 6.95 |
|  | Mean error, CI 95% Upper Limit (mmHg) |  | -6.50 | -3.48 |  | -6.79 | -1.00 |  | -6.21 | -2.52 |  | -5.00 | -4.94 |
|  | Mean error, CI 95% Lower Limit (mmHg) | ˗ | -7.91 | -5.55 | ˗ | -9.08 | -3.27 | ˗ | -8.79 | -6.39 | ˗ | -7.41 | -8.97 |
|  | p | ˗ | **<0.001** | **<0.001** | ˗ | **<0.001** | **<0.001** | ˗ | **<0.001** | **<0.001** | ˗ | **<0.001** | **<0.001** |
|  | Mean error, SD (mmHg) | ˗ | 5.72 | 8.46 | ˗ | 5.15 | 5.12 | ˗ | 6.31 | 9.50 | ˗ | 5.45 | 16.33 |
|  | Upper limit (mmHg) | ˗ | 4.00 | 12.07 | ˗ | 2.15 | 7.91 | ˗ | 4.87 | 14.17 | ˗ | 4.48 | 11.15 |
|  | Lower limit (mmHg) | ˗ | -18.41 | -21.10 | ˗ | -18.03 | -12.18 | ˗ | -19.87 | -23.07 | ˗ | -16.90 | -25.05 |
|  | Regression equation | ˗ | y= 9.0 - 0.2x | y= 21.2 - 0.2x | ˗ | y=12.8 - 0.2x | y= 11.5 - 0.1x | ˗ | y= 19.9 - 0.3x | y= 17.8 - 0.2x | ˗ | y= 26.6 - 0.3x | y= 37.2 - 0.4x |
|  | p(ϐ) | ˗ | **<0.001** | **<0.001** | ˗ | **0.00** | **0.05** | ˗ | **<0.001** | **0.03** | ˗ | **<0.001** | **<0.001** |
| **Carotid tonometry (SCOR)** | r | 0.89 | ˗ | 0.71 | 0.83 | ˗ | 0.71 | 0.88 | ˗ | 0.61 | 0.87 | ˗ | 0.59 |
|  | p | **<0.001** | ˗ | **<0.001** | **<0.001** | ˗ | **<0.001** | **<0.001** | ˗ | **<0.001** | **<0.001** | ˗ | **<0.001** |
|  | Mean error (mmHg) | 7.21 | ˗ | 2.79 | 7.94 | ˗ | 6.02 | 7.50 | ˗ | 3.14 | 6.21 | ˗ | -0.91 |
|  | Mean error, CI 95% Upper Limit (mmHg) | 7.91 | ˗ | 4.00 | 9.08 |  | 7.56 | 8.79 |  | 5.35 | 7.42 |  | 1.35 |
|  | Mean error, CI 95% Lower Limit (mmHg) | 6.50 |  | 1.58 | 6.79 | ˗ | 4.49 | 6.21 | ˗ | 0.93 | 5.00 | ˗ | -3.18 |
|  | p | **<0.001** | ˗ | **<0.001** | **<0.001** | ˗ | **<0.001** | **<0.001** | ˗ | **0.01** | **<0.001** | ˗ | 0.43 |
|  | Mean error, SD (mmHg) | 5.72 | ˗ | 9.87 | 5.15 | ˗ | 6.98 | 6.31 | ˗ | 10.79 | 5.45 | ˗ | 10.18 |
|  | Upper limit (mmHg) | 18.41 | ˗ | 22.14 | 18.03 | ˗ | 19.71 | 19.87 | ˗ | 24.29 | 16.90 | ˗ | 19.05 |
|  | Lower limit (mmHg) | -4.00 | ˗ | -16.56 | -2.15 | ˗ | -7.66 | -4.87 | ˗ | -18.02 | -4.48 | ˗ | -20.87 |
|  | Regression equation | y= -9.0 + 0.2x | ˗ | y= 11.1 - 0.08x | y=-12.8 + 0.2x | ˗ | y=-4.1 + 0.1x | y= -19.9 + 0.3x | ˗ | y= -12.9 + 0.1x | y= -26.6 + 0.3x | ˗ | y= 7.5 - 0.07x |
|  | p(ϐ) | **<0.001** | ˗ | 0.13 | **0.00** | ˗ | 0.26 | **<0.001** | ˗ | 0.15 | **<0.001** | ˗ | 0.53 |
| **Brachial oscillometry (MOG)** | r | 0.78 | 0.71 | ˗ | 0.81 | 0.71 | ˗ | 0.65 | 0.61 | ˗ | 0.61 | 0.59 | ˗ |
|  | p | **<0.001** | **<0.001** | ˗ | **<0.001** | **<0.001** | ˗ | **<0.001** | **<0.001** | ˗ | **<0.001** | **<0.001** | ˗ |
|  | Mean error (mmHg) | 4.52 | -2.79 | ˗ | 2.14 | -6.02 | ˗ | 4.45 | -3.14 | ˗ | 6.95 | 0.91 | ˗ |
|  | Mean error, CI 95% Upper Limit (mmHg) | 5.55 | -1.58 | ˗ | 3.27 | -4.49 |  | 6.39 | -0.93 |  | 8.97 | 3.18 |  |
|  | Mean error, CI 95% Lower Limit (mmHg) | 3.48 | -4.00 |  | 1.00 | -7.56 | ˗ | 2.52 | -5.35 | ˗ | 4.94 | -1.35 | ˗ |
|  | p | **<0.001** | **<0.001** | ˗ | **<0.001** | **<0.001** | ˗ | **<0.001** | **0.01** | ˗ | **<0.001** | 0.43 | ˗ |
|  | Mean error, SD (mmHg) | 8.46 | 9.87 | ˗ | 5.12 | 6.98 | ˗ | 9.50 | 10.79 | ˗ | 9.23 | 10.18 | ˗ |
|  | Upper limit (mmHg) | 21.10 | 16.56 | ˗ | 12.18 | 7.66 | ˗ | 23.07 | 18.02 | ˗ | 25.05 | 20.87 | ˗ |
|  | Lower limit (mmHg) | -12.07 | -22.14 | ˗ | -7.91 | -19.71 | ˗ | -14.17 | -24.29 | ˗ | -11.15 | -19.05 | ˗ |
|  | Regression equation | y= -21.2 + 0.2x | y= -11.1 + 0.08x | ˗ | y= -11.5 + 0.1x | y= 4.1 - 0.1x | ˗ | y= -17.8 + 0.2x | y= 12.9 - 0.1x | ˗ | y= -37.2 + 0.4x | y= -7.5 + 0.07x | ˗ |
|  | p(ϐ) | **<0.001** | 0.13 | ˗ | **0.05** | 0.26 | ˗ | **0.03** | 0.15 | ˗ | **<0.001** | 0.53 | ˗ |
| RT: radial applanation tonometry record, obtained with SphygmoCor device (SCOR). CT: carotid applanation tonometry record, obtained with SCOR. BOSC: brachial oscillometry/plethysmography record, obtained with Mobil-O-Graph device (MOG). cSBP: central systolic blood pressure. r: correlation (Pearson) coefficient. β: slope of regression equation. Significance level: p value <0.05 (red text). Bland-Altman analysis: variable "x" was considered the mean of both methods compared (eg. (RT+CT)/2) and variable "y" the difference among first and second method (eg. RT minus CT). MBPc: mean blood pressure calculated as pDBP+((pSBP-pDBP)/3). CI: confidence interval. | | | | | | | | | | | | | |
|  |  |  |  |  |  |  |  |  |  |  |  |  |  |
|  |  |  |  |  |  |  |  |  |  |  |  |  |  |
|  |  |  |  |  |  |  |  |  |  |  |  |  |  |
