# Supplementary material for: Aortic pressure and forward and backward wave components in children, adolescents and young-adults: Agreement between brachial oscillometry, radial and carotid tonometry data and analysis of factors associated with their differences
Source: PLoS One. 2019 Dec 19;14(12):e0226709. doi: 10.1371/journal.pone.0226709 (PMC6922407; doi:10.1371/journal.pone.0226709)
Supplement: S13 Table — (DOCX) [file pone.0226709.s031.docx]

| **S13 Table. cPP: agreement among parameters measured with three different methods in the entire and age-related groups, calibrated with identical peripheral blood pressure levels obtained by oscillometry (Calibration scheme: pDBP/MBPc) [Extended table]** | | | | | | | | | | | | | |
| --- | --- | --- | --- | --- | --- | --- | --- | --- | --- | --- | --- | --- | --- |
|  |  |  |  |  |  |  |  |  |  |  |  |  |  |
|  |  |  |  |  |  |  |  |  |  |  |  |  |  |
| **cPP** | | **Entire group [3-35 years]** | | | **Children [3-12 years]** | | | **Adolescents [12-18 years]** | | | **Young adults [18-35 years]** | | |
|  |  | **RT (SCOR)** | **CT (SCOR)** | **BOSC (MOG)** | **RT (SCOR)** | **CT (SCOR)** | **BOSC (MOG)** | **RT (SCOR)** | **CT (SCOR)** | **BOSC (MOG)** | **RT (SCOR)** | **CT (SCOR)** | **BOSC (MOG)** |
| **Radial tonometry (SCOR)** | r | ˗ | 0.83 | 0.77 | ˗ | 0.73 | 0.80 | ˗ | 0.84 | 0.68 | ˗ | 0.85 | 0.79 |
|  | p | ˗ | **<0.001** | **<0.001** | ˗ | **<0.001** | **<0.001** | ˗ | **<0.001** | **<0.001** | ˗ | **<0.001** | **<0.001** |
|  | Mean error (mmHg) | ˗ | -8.47 | -4.67 | ˗ | -9.30 | -2.52 | ˗ | -8.82 | -4.44 | ˗ | -7.30 | -7.07 |
|  | Mean error, CI 95% Upper Limit (mmHg) |  | -7.73 | -3.70 |  | -8.14 | -1.43 |  | -7.47 | -2.71 |  | -6.01 | -5.08 |
|  | Mean error, CI 95% Lower Limit (mmHg) | ˗ | -9.20 | -5.64 | ˗ | -10.46 | -3.61 | ˗ | -10.17 | -6.16 | ˗ | -8.58 | -9.07 |
|  | p | ˗ | **<0.001** | **<0.001** | ˗ | **<0.001** | **<0.001** | ˗ | **<0.001** | **<0.001** | ˗ | **<0.001** | **<0.001** |
|  | Mean error, SD (mmHg) | ˗ | 6.00 | 7.94 | ˗ | 5.20 | 4.91 | ˗ | 6.64 | 8.44 | ˗ | 5.82 | 9.13 |
|  | Upper limit (mmHg) | ˗ | 3.28 | 10.89 | ˗ | 0.89 | 7.11 | ˗ | 4.19 | 12.10 | ˗ | 4.12 | 10.83 |
|  | Lower limit (mmHg) | ˗ | -20.22 | -20.23 | ˗ | -19.49 | -12.15 | ˗ | -21.83 | -20.97 | ˗ | -18.71 | -24.97 |
|  | Regression equation | ˗ | y= 0.9 - 0.2x | y= 10.0 - 0.4x | ˗ | y= -2.0 - 0.2x | y= 4.4 - 0.2x | ˗ | y= 3.7 - 0.3x | y= 6.0- 0.3x | ˗ | y= 5.0 - 0.3x | y= 15.9 - 0.6x |
|  | p(ϐ) | ˗ | **<0.001** | **<0.001** | ˗ | **0.02** | **0.00** | ˗ | **<0.001** | **0.00** | ˗ | **<0.001** | **<0.001** |
| **Carotid tonometry (SCOR)** | r | 0.83 | ˗ | 0.64 | 0.73 | ˗ | 0.59 | 0.84 | ˗ | 0.59 | 0.85 | ˗ | 0.69 |
|  | p | **<0.001** | ˗ | **<0.001** | **<0.001** | ˗ | **<0.001** | **<0.001** | ˗ | **<0.001** | **<0.001** | ˗ | **<0.001** |
|  | Mean error (mmHg) | 8.47 | ˗ | 3.83 | 9.30 | ˗ | 7.04 | 8.82 | ˗ | 4.33 | 7.30 | ˗ | 0.06 |
|  | Mean error, CI 95% Upper Limit (mmHg) | 9.20 |  | 5.06 | 10.46 |  | 8.58 | 10.17 |  | 6.54 | 8.58 |  | 2.37 |
|  | Mean error, CI 95% Lower Limit (mmHg) | 7.73 | ˗ | 2.60 | 8.14 | ˗ | 5.50 | 7.47 | ˗ | 2.12 | 6.01 | ˗ | -2.25 |
|  | p | **<0.001** | ˗ | **<0.001** | **<0.001** | ˗ | **<0.001** | **<0.001** | ˗ | **<0.001** | **<0.001** | ˗ | 0.96 |
|  | Mean error, SD (mmHg) | 6.00 | ˗ | 9.95 | 5.20 | ˗ | 6.92 | 6.64 | ˗ | 10.81 | 5.82 | ˗ | 10.38 |
|  | Upper limit (mmHg) | 20.22 | ˗ | 23.34 | 19.49 | ˗ | 20.60 | 21.83 | ˗ | 25.52 | 18.71 | ˗ | 20.41 |
|  | Lower limit (mmHg) | -3.28 | ˗ | -15.67 | -0.89 | ˗ | -6.52 | -4.19 | ˗ | -16.86 | -4.12 | ˗ | -20.28 |
|  | Regression equation | y= -0.9 + 0.2x | ˗ | y= 10.1 - 0.2x | y= 2.0 + 0.2x | ˗ | y= 6.2 + 0.02x | y= -3.7 + 0.3x | ˗ | y= -0.06 + 0.1x | y= -5.0 + 0.3x | ˗ | y= 13.6 - 0.3x |
|  | p(ϐ) | **<0.001** | ˗ | 0.01 | **0.02** | ˗ | 0.84 | **<0.001** | ˗ | 0.33 | **<0.001** | ˗ | **0.00** |
| **Brachial oscillometry (MOG)** | r | 0.77 | 0.64 | ˗ | 0.80 | 0.59 | ˗ | 0.68 | 0.59 | ˗ | 0.79 | 0.69 | ˗ |
|  | p | **<0.001** | **<0.001** | ˗ | **<0.001** | **<0.001** | ˗ | **<0.001** | **<0.001** | ˗ | **<0.001** | **<0.001** | ˗ |
|  | Mean error (mmHg) | -4.67 | 3.83 | ˗ | 2.52 | -7.04 | ˗ | 4.44 | 4.33 | ˗ | 7.07 | -0.06 | ˗ |
|  | Mean error, CI 95% Upper Limit (mmHg) | -3.70 | 5.06 |  | 3.61 | -5.50 |  | 6.16 | -2.12 |  | 9.07 | 2.25 |  |
|  | Mean error, CI 95% Lower Limit (mmHg) | -5.64 | 2.60 | ˗ | 1.43 | -8.58 | ˗ | 2.71 | -6.54 | ˗ | 5.08 | -2.37 | ˗ |
|  | p | **<0.001** | **<0.001** | ˗ | **<0.001** | **<0.001** | ˗ | **<0.001** | **<0.001** | ˗ | **<0.001** | 0.96 | ˗ |
|  | Mean error, SD (mmHg) | 7.94 | 9.95 | ˗ | 4.91 | 6.92 | ˗ | 8.44 | 15.23 | ˗ | 9.13 | 10.38 | ˗ |
|  | Upper limit (mmHg) | 10.89 | 23.34 | ˗ | 12.15 | 6.52 | ˗ | 20.97 | 16.86 | ˗ | 24.97 | 20.28 | ˗ |
|  | Lower limit (mmHg) | -20.23 | -15.67 | ˗ | -7.11 | -20.60 | ˗ | -12.10 | -25.52 | ˗ | -10.83 | -20.41 | ˗ |
|  | Regression equation | y= 10.0 - 0.4x | y= 10.1 - 0.2x | ˗ | y= -4.4 + 0.2x | y= -6.2 - 0.02x | ˗ | y= -6.0 + 0.3x | y= 0.06 - 0.1x | ˗ | y= -15.9 + 0.6x | y= -13.6 + 0.3x | ˗ |
|  | p(ϐ) | **<0.001** | 0.01 | ˗ | **0.00** | 0.84 | ˗ | **0.00** | 0.33 | ˗ | **<0.001** | **0.00** | ˗ |
| RT: radial applanation tonometry record, obtained with SphygmoCor device (SCOR). CT: carotid applanation tonometry record, obtained with SCOR. BOSC: brachial oscillometry/plethysmography record, obtained with Mobil-O-Graph device (MOG). cPP: central pulse pressure. r: correlation (Pearson) coefficient. β: slope of regression equation. Significance level: p value <0.05 (red text). Bland-Altman analysis: variable "x" was considered the mean of both methods compared (eg. (RT+CT)/2) and variable "y" the difference among first and second method (eg. RT minus CT). MBPc: mean blood pressure calculated as pDBP+((pSBP-pDBP)/3). CI: confidence interval. | | | | | | | | | | | | | |
|  |  |  |  |  |  |  |  |  |  |  |  |  |  |
|  |  |  |  |  |  |  |  |  |  |  |  |  |  |
