# Supplementary material for: Aortic pressure and forward and backward wave components in children, adolescents and young-adults: Agreement between brachial oscillometry, radial and carotid tonometry data and analysis of factors associated with their differences
Source: PLoS One. 2019 Dec 19;14(12):e0226709. doi: 10.1371/journal.pone.0226709 (PMC6922407; doi:10.1371/journal.pone.0226709)
Supplement: S14 Table — (DOCX) [file pone.0226709.s032.docx]

| **S14 Table. Pf: agreement among parameters measured with three different methods in the entire and age-related groups, calibrated with identical peripheral blood pressure levels obtained by oscillometry (Calibration scheme: pDBP/MBPc) [Extended table]** | | | | | | | | | | | | | |
| --- | --- | --- | --- | --- | --- | --- | --- | --- | --- | --- | --- | --- | --- |
|  |  |  |  |  |  |  |  |  |  |  |  |  |  |
|  |  |  |  |  |  |  |  |  |  |  |  |  |  |
| **Pf** | | **Entire group [3-35 years]** | | | **Children [3-12 years]** | | | **Adolescents [12-18 years]** | | | **Young adults [18-35 years]** | | |
|  |  | **RT (SCOR)** | **CT (SCOR)** | **BOSC (MOG)** | **RT (SCOR)** | **CT (SCOR)** | **BOSC (MOG)** | **RT (SCOR)** | **CT (SCOR)** | **BOSC (MOG)** | **RT (SCOR)** | **CT (SCOR)** | **BOSC (MOG)** |
| **Radial tonometry (SCOR)** | r | ˗ | 0.79 | 0.75 | ˗ | 0.70 | 0.75 | ˗ | 0.79 | 0.68 | ˗ | 0.82 | 0.77 |
|  | p | ˗ | **<0.001** | **<0.001** | ˗ | **<0.001** | **<0.001** | ˗ | **<0.001** | **<0.001** | ˗ | **<0.001** | **<0.001** |
|  | Mean error (mmHg) | ˗ | -8.10 | 6.61 | ˗ | -9.64 | 6.38 | ˗ | -7.68 | 7.13 | ˗ | -7.43 | 6.27 |
|  | Mean error, CI 95% Upper Limit (mmHg) |  | -7.20 | 7.37 |  | -8.05 | 7.38 |  | -6.13 | 8.67 |  | -5.88 | 7.65 |
|  | Mean error, CI 95% Lower Limit (mmHg) | ˗ | -9.00 | 5.85 | ˗ | -11.23 | 5.38 | ˗ | -9.22 | 5.60 | ˗ | -8.99 | 4.90 |
|  | p | ˗ | **<0.001** | **<0.001** | ˗ | **<0.001** | **<0.001** | ˗ | **<0.001** | **<0.001** | ˗ | **<0.001** | **<0.001** |
|  | Mean error, SD (mmHg) | ˗ | 6.01 | 6.03 | ˗ | 5.42 | 4.47 | ˗ | 6.37 | 7.27 | ˗ | 5.91 | 5.89 |
|  | Upper limit (mmHg) | ˗ | 3.68 | 18.42 | ˗ | 0.99 | 15.15 | ˗ | 4.82 | 21.38 | ˗ | 4.16 | 17.83 |
|  | Lower limit (mmHg) | ˗ | -19.87 | -5.20 | ˗ | -20.27 | -2.38 | ˗ | -20.17 | -7.11 | ˗ | -19.02 | -5.28 |
|  | Regression equation | ˗ | y= -1.9 - 0.2x | y= 3.0 + 0.1x | ˗ | y= -1.4 - 0.2x | y= 0.9 + 0.2x | ˗ | y= 0.8 - 0.2x | y= 0.2 + 0.2x | ˗ | y= -0.9 - 0.2x | y= 7.3 - 0.03x |
|  | p(ϐ) | ˗ | **0.00** | **0.01** | ˗ | 0.05 | **0.01** | ˗ | **0.01** | **0.02** | ˗ | **0.05** | 0.71 |
| **Carotid tonometry (SCOR)** | r | 0.79 | ˗ | 0.66 | 0.70 | ˗ | 0.54 | 0.79 | ˗ | 0.70 | 0.82 | ˗ | 0.64 |
|  | p | **<0.001** | ˗ | **<0.001** | **<0.001** | ˗ | **<0.001** | **<0.001** | ˗ | **<0.001** | **<0.001** | ˗ | **<0.001** |
|  | Mean error (mmHg) | 8.10 | ˗ | 14.22 | 9.64 | ˗ | 16.02 | 7.68 | ˗ | 13.64 | 7.43 | ˗ | 13.68 |
|  | Mean error, CI 95% Upper Limit (mmHg) | 9.00 |  | 15.31 | 11.23 |  | 17.87 | 9.22 |  | 15.37 | 8.99 |  | 15.76 |
|  | Mean error, CI 95% Lower Limit (mmHg) | 7.20 | ˗ | 13.13 | 8.05 | ˗ | 14.17 | 6.13 | ˗ | 11.91 | 5.88 | ˗ | 11.61 |
|  | p | **<0.001** | ˗ | **<0.001** | **<0.001** | ˗ | **<0.001** | **<0.001** | ˗ | **<0.001** | **<0.001** | ˗ | **<0.001** |
|  | Mean error, SD (mmHg) | 6.01 | ˗ | 7.50 | 5.42 | ˗ | 6.31 | 6.37 | ˗ | 7.25 | 5.91 | ˗ | 8.37 |
|  | Upper limit (mmHg) | 19.87 | ˗ | 28.91 | 20.27 | ˗ | 28.39 | 20.17 | ˗ | 27.86 | 19.02 | ˗ | 30.09 |
|  | Lower limit (mmHg) | -3.68 | ˗ | -0.47 | -0.99 | ˗ | 3.65 | -4.82 | ˗ | -0.58 | -4.16 | ˗ | -2.73 |
|  | Regression equation | y= 1.9 + 0.2x | ˗ | y= 7.1 + 0.2x | y= 1.4 + 0.2x | ˗ | y= -4.1 + 0.7x | y= -0.8 + 0.2x | ˗ | y= 5.2 + 0.2x | y= 0.9 + 0.2x | ˗ | y= 8.0 + 0.2x |
|  | p(ϐ) | **0.00** | ˗ | **0.00** | 0.05 | ˗ | **<0.001** | **0.01** | ˗ | **0.02** | **0.05** | ˗ | 0.17 |
| **Brachial oscillometry (MOG)** | r | 0.75 | 0.66 | ˗ | 0.75 | 0.54 | ˗ | 0.68 | 0.70 | ˗ | 0.77 | 0.64 | ˗ |
|  | p | **<0.001** | **<0.001** | ˗ | **<0.001** | **<0.001** | ˗ | **<0.001** | **<0.001** | ˗ | **<0.001** | **<0.001** | ˗ |
|  | Mean error (mmHg) | -6.61 | -14.22 | ˗ | -6.38 | -16.02 | ˗ | -7.13 | -13.64 | ˗ | -6.27 | -13.68 | ˗ |
|  | Mean error, CI 95% Upper Limit (mmHg) | -5.85 | -13.13 |  | -5.38 | -14.17 |  | -5.60 | -11.91 |  | -4.90 | -11.61 |  |
|  | Mean error, CI 95% Lower Limit (mmHg) | -7.37 | -15.31 | ˗ | -7.38 | -17.87 | ˗ | -8.67 | -15.37 | ˗ | -7.65 | -15.76 | ˗ |
|  | p | **<0.001** | **<0.001** | ˗ | **<0.001** | **<0.001** | ˗ | **<0.001** | **<0.001** | ˗ | **<0.001** | **<0.001** | ˗ |
|  | Mean error, SD (mmHg) | 6.03 | 7.50 | ˗ | 4.48 | 6.31 | ˗ | 7.27 | 7.25 | ˗ | 5.89 | 8.37 | ˗ |
|  | Upper limit (mmHg) | 5.20 | 0.47 | ˗ | 2.39 | -3.65 | ˗ | 7.11 | 0.58 | ˗ | 5.28 | 2.73 | ˗ |
|  | Lower limit (mmHg) | -18.42 | -28.91 | ˗ | -15.15 | -28.39 | ˗ | -21.38 | -27.86 | ˗ | -17.83 | -30.09 | ˗ |
|  | Regression equation | y= -3.0 - 0.1x | y= -7.1 - 0.2x | ˗ | y= -0.9 - 0.2x | y= -4.1 + 0.7x | ˗ | y= -0.2 - 0.2x | y= -5.2 - 0.2x | ˗ | y= -7.3 + 0.03x | y= -8.0 - 0.2x | ˗ |
|  | p(ϐ) | **0.01** | **0.00** | ˗ | **0.01** | **<0.001** | ˗ | **0.02** | **0.02** | ˗ | 0.71 | 0.17 | ˗ |
| RT: radial applanation tonometry record, obtained with SphygmoCor device (SCOR). CT: carotid applanation tonometry record, obtained with SCOR. BOSC: brachial oscillometry/plethysmography record, obtained with Mobil-O-Graph device (MOG). Pf: forward wave height (amplitude). r: correlation (Pearson) coefficient. β: slope of regression equation. Significance level: p value <0.05 (red text). Bland-Altman analysis: variable "x" was considered the mean of both methods compared (eg. (RT+CT)/2) and variable "y" the difference among first and second method (eg. RT minus CT). MBPc: mean blood pressure calculated as pDBP+((pSBP-pDBP)/3). CI: confidence interval. | | | | | | | | | | | | | |
|  |  |  |  |  |  |  |  |  |  |  |  |  |  |
|  |  |  |  |  |  |  |  |  |  |  |  |  |  |
|  |  |  |  |  |  |  |  |  |  |  |  |  |  |
