# Supplementary material for: Aortic pressure and forward and backward wave components in children, adolescents and young-adults: Agreement between brachial oscillometry, radial and carotid tonometry data and analysis of factors associated with their differences
Source: PLoS One. 2019 Dec 19;14(12):e0226709. doi: 10.1371/journal.pone.0226709 (PMC6922407; doi:10.1371/journal.pone.0226709)
Supplement: S15 Table — (DOCX) [file pone.0226709.s033.docx]

| **S15 Table. Pb: agreement among parameters measured with three different methods in the entire and age-related groups, calibrated with identical peripheral blood pressure levels obtained by oscillometry (Calibration scheme: pDBP/MBPc) [Extended table]** | | | | | | | | | | | | | |
| --- | --- | --- | --- | --- | --- | --- | --- | --- | --- | --- | --- | --- | --- |
|  |  |  |  |  |  |  |  |  |  |  |  |  |  |
|  |  |  |  |  |  |  |  |  |  |  |  |  |  |
| **Pb** | | **Entire group [3-35 years]** | | | **Children [3-12 years]** | | | **Adolescents [12-18 years]** | | | **Young adults [18-35 years]** | | |
|  |  | **RT (SCOR)** | **CT (SCOR)** | **BOSC (MOG)** | **RT (SCOR)** | **CT (SCOR)** | **BOSC (MOG)** | **RT (SCOR)** | **CT (SCOR)** | **BOSC (MOG)** | **RT (SCOR)** | **CT (SCOR)** | **BOSC (MOG)** |
| **Radial tonometry (SCOR)** | r | ˗ | 0.83 | 0.61 | ˗ | 0.78 | 0.71 | ˗ | 0.84 | 0.56 | ˗ | 0.86 | 0.58 |
|  | p | ˗ | **<0.001** | **<0.001** | ˗ | **<0.001** | **<0.001** | ˗ | **<0.001** | **<0.001** | ˗ | **<0.001** | **<0.001** |
|  | Mean error (mmHg) | ˗ | -1.84 | -1.93 | ˗ | -0.87 | -0.02 | ˗ | -1.84 | -2.55 | ˗ | -2.60 | -3.32 |
|  | Mean error, CI 95% Upper Limit (mmHg) |  | -1.55 | -1.36 |  | -0.33 | 0.54 |  | -1.41 | -1.51 |  | -2.08 | -2.15 |
|  | Mean error, CI 95% Lower Limit (mmHg) |  | -2.13 | -2.50 |  | -1.42 | -0.58 | ˗ | -2.27 | -3.59 | ˗ | -3.12 | -4.49 |
|  | p | ˗ | **<0.001** | **<0.001** | ˗ | **0.00** | 0.94 | ˗ | **<0.001** | **<0.001** | ˗ | **<0.001** | **<0.001** |
|  | Mean error, SD (mmHg) | ˗ | 1.97 | 4.52 | ˗ | 1.86 | 2.51 | ˗ | 1.79 | 4.95 | ˗ | 1.97 | 5.01 |
|  | Upper limit (mmHg) | ˗ | 2.03 | 6.94 | ˗ | 2.77 | 4.91 | ˗ | 1.66 | 7.16 | ˗ | 1.26 | 6.50 |
|  | Lower limit (mmHg) | ˗ | -5.71 | -10.80 | ˗ | -4.52 | -4.95 | ˗ | -5.34 | -12.26 | ˗ | -6.47 | -13.14 |
|  | Regression equation | ˗ | y= 0.7 - 0.2x | y= 8.6 - 0.7x | ˗ | y= -0.8 - 0.002x | y= 2.7 - 0.2x | ˗ | y= -0.9 - 0.07x | y= 9.0 - 0.8x | ˗ | y= 1.5- 0.3x | y= 10.6 - 0.9x |
|  | p(ϐ) | ˗ | **<0.001** | **<0.001** | ˗ | 0.99 | **0.02** | ˗ | 0.34 | **<0.001** | ˗ | **0.00** | **<0.001** |
| **Carotid tonometry (SCOR)** | r | 0.83 | ˗ | 0.60 | 0.78 | ˗ | 0.54 | 0.84 | ˗ | 0.62 | 0.86 | ˗ | 0.53 |
|  | p | **<0.001** | ˗ | **<0.001** | **<0.001** | ˗ | **<0.001** | **<0.001** | ˗ | **<0.001** | **<0.001** | ˗ | **<0.001** |
|  | Mean error (mmHg) | 1.84 | ˗ | -0.68 | 0.87 | ˗ | 0.64 | 1.84 | ˗ | -1.10 | 2.60 | ˗ | -1.25 |
|  | Mean error, CI 95% Upper Limit (mmHg) | 2.13 | ˗ | 0.01 | 1.42 |  | 1.51 | 2.27 |  | 0.05 | 3.12 |  | 0.08 |
|  | Mean error, CI 95% Lower Limit (mmHg) | 1.55 |  | -1.37 | 0.33 | ˗ | -0.24 | 1.41 | ˗ | -2.25 | 2.08 | ˗ | -2.58 |
|  | p | **<0.001** | ˗ | 0.05 | **0.00** | ˗ | 0.15 | **<0.001** | ˗ | 0.06 | **<0.001** | ˗ | 0.07 |
|  | Mean error, SD (mmHg) | 1.97 | ˗ | 4.73 | 1.86 | ˗ | 2.98 | 1.79 | ˗ | 4.90 | 1.97 | ˗ | 5.38 |
|  | Upper limit (mmHg) | 5.71 | ˗ | 8.59 | 4.52 | ˗ | 6.47 | 5.34 | ˗ | 8.49 | 6.47 | ˗ | 9.30 |
|  | Lower limit (mmHg) | -2.03 | ˗ | -9.95 | -2.77 | ˗ | -5.20 | -1.66 | ˗ | -10.70 | -1.26 | ˗ | -11.80 |
|  | Regression equation | y= -0.7 + 0.2x | ˗ | y= 9.7 - 0.7x | y= 0.8 + 0.002x | ˗ | y= 3.4 -0.2x | y= 0.9 + 0.07x | ˗ | y= 11.0 - 0.8x | y= -1.5 + 0.3x | ˗ | y= 11.0 - 0.7x |
|  | p(ϐ) | **<0.001** | ˗ | **<0.001** | 0.99 | ˗ | 0.20 | 0.34 | ˗ | **<0.001** | **0.00** | ˗ | **<0.001** |
| **Brachial oscillometry (MOG)** | r | 0.61 | 0.60 | ˗ | 0.71 | 0.54 | ˗ | 0.56 | 0.62 | ˗ | 0.58 | 0.53 | ˗ |
|  | p | **<0.001** | **<0.001** | ˗ | **<0.001** | **<0.001** | ˗ | **<0.001** | **<0.001** | ˗ | **<0.001** | **<0.001** | ˗ |
|  | Mean error (mmHg) | 1.93 | 0.68 | ˗ | 0.02 | -0.64 | ˗ | 2.55 | 1.10 | ˗ | 3.32 | 1.25 | ˗ |
|  | Mean error, CI 95% Upper Limit (mmHg) | 2.50 | 1.37 |  | 0.58 | 0.24 |  | 3.59 | 2.25 |  | 4.49 | 2.58 |  |
|  | Mean error, CI 95% Lower Limit (mmHg) | 1.36 | -0.01 |  | -0.54 | -1.51 | ˗ | 1.51 | -0.05 | ˗ | 2.15 | -0.08 |  |
|  | p | **<0.001** | 0.05 | ˗ | 0.94 | 0.15 | ˗ | **<0.001** | 0.06 | ˗ | **<0.001** | 0.07 | ˗ |
|  | Mean error, SD (mmHg) | 4.52 | 4.73 | ˗ | 2.51 | 2.98 | ˗ | 4.95 | 4.90 | ˗ | 5.01 | 5.38 | ˗ |
|  | Upper limit (mmHg) | 10.80 | 9.95 | ˗ | 4.95 | 5.20 | ˗ | 12.26 | 10.70 | ˗ | 13.14 | 11.80 | ˗ |
|  | Lower limit (mmHg) | -6.94 | -8.59 | ˗ | -4.91 | -6.47 | ˗ | -7.16 | -8.49 | ˗ | -6.50 | -9.30 | ˗ |
|  | Regression equation | y= -8.6 + 0.7x | y= -9.7 + 0.7x | ˗ | y= -2.7 + 0.2x | y= -3.4 + 0.2x | ˗ | y= -9.0 + 0.8x | y= -11.0 + 0.8x | ˗ | y= -10.6 + 0.9x | y= -11.0 + 0.7x | ˗ |
|  | p(ϐ) | **<0.001** | **<0.001** | ˗ | **0.02** | 0.20 | ˗ | **<0.001** | **<0.001** | ˗ | **<0.001** | **<0.001** | ˗ |
| RT: radial applanation tonometry record, obtained with SphygmoCor device (SCOR). CT: carotid applanation tonometry record, obtained with SCOR. BOSC: brachial oscillometry/plethysmography record, obtained with Mobil-O-Graph device (MOG). Pb: backward wave height (amplitude). r: correlation (Pearson) coefficient. β: slope of regression equation. Significance level: p value <0.05 (red text). Bland-Altman analysis: variable "x" was considered the mean of both methods compared (eg. (RT+CT)/2) and variable "y" the difference among first and second method (eg. RT minus CT). MBPc: mean blood pressure calculated as pDBP+((pSBP-pDBP)/3). CI: confidence interval. | | | | | | | | | | | | | |
|  |  |  |  |  |  |  |  |  |  |  |  |  |  |
|  |  |  |  |  |  |  |  |  |  |  |  |  |  |
|  |  |  |  |  |  |  |  |  |  |  |  |  |  |
