# Supplementary material for: Aortic pressure and forward and backward wave components in children, adolescents and young-adults: Agreement between brachial oscillometry, radial and carotid tonometry data and analysis of factors associated with their differences
Source: PLoS One. 2019 Dec 19;14(12):e0226709. doi: 10.1371/journal.pone.0226709 (PMC6922407; doi:10.1371/journal.pone.0226709)
Supplement: S16 Table — (DOCX) [file pone.0226709.s034.docx]

| **S16 Table. cSBP: agreement among parameters measured with three different methods in the entire and age-related groups, calibrated with identical peripheral blood pressure levels obtained by oscillometry (Calibration scheme: pDBP/MBPosc) [Extended table]** | | | | | | | | | | | | | |
| --- | --- | --- | --- | --- | --- | --- | --- | --- | --- | --- | --- | --- | --- |
|  |  |  |  |  |  |  |  |  |  |  |  |  |  |
|  |  |  |  |  |  |  |  |  |  |  |  |  |  |
| **cSBP** | | **Entire group [3-35 years]** | | | **Children [3-12 years]** | | | **Adolescents [12-18 years]** | | | **Young adults [18-35 years]** | | |
|  |  | **RT (SCOR)** | **CT (SCOR)** | **BOSC (MOG)** | **RT (SCOR)** | **CT (SCOR)** | **BOSC (MOG)** | **RT (SCOR)** | **CT (SCOR)** | **BOSC (MOG)** | **RT (SCOR)** | **CT (SCOR)** | **BOSC (MOG)** |
| **Radial tonometry (SCOR)** | r | ˗ | 0.83 | 0.77 | ˗ | 0.75 | 0.78 | ˗ | 0.83 | 0.65 | ˗ | 0.83 | 0.60 |
|  | p | ˗ | **<0.001** | **<0.001** | ˗ | **<0.001** | **<0.001** | ˗ | **<0.001** | **<0.001** | ˗ | **<0.001** | **<0.001** |
|  | Mean error (mmHg) | ˗ | -10.46 | -6.60 | ˗ | -12.09 | -3.57 | ˗ | -10.37 | -6.55 | ˗ | -9.05 | -9.83 |
|  | Mean error, CI 95% Upper Limit (mmHg) |  | -9.37 | -5.25 |  | -10.24 | -2.01 |  | -8.42 | -4.05 |  | -7.20 | -7.18 |
|  | Mean error, CI 95% Lower Limit (mmHg) |  | -11.55 | -7.94 |  | -13.93 | -5.12 | ˗ | -12.32 | -9.05 | ˗ | -10.90 | -12.48 |
|  | p | ˗ | **<0.001** | **<0.001** | ˗ | **<0.001** | **<0.001** | ˗ | **<0.001** | **<0.001** | ˗ | **<0.001** | **<0.001** |
|  | Mean error, SD (mmHg) | ˗ | 8.87 | 11.06 | ˗ | 8.35 | 6.98 | ˗ | 9.56 | 12.34 | ˗ | 8.40 | 12.05 |
|  | Upper limit (mmHg) | ˗ | 6.93 | 15.08 | ˗ | 4.28 | 10.39 | ˗ | 8.36 | 17.64 | ˗ | 7.42 | 13.79 |
|  | Lower limit (mmHg) | ˗ | -27.85 | -28.27 | ˗ | -28.45 | -17.25 | ˗ | -29.10 | -30.74 | ˗ | -25.52 | -33.45 |
|  | Regression equation | ˗ | y= 15.8 - 0.2x | y= 28.7 - 0.3x | ˗ | y= 20.5 + 0.3x | y= 14.8 - 0.2x | ˗ | y= 29.2 - 0.3x | y= 25.3 - 0.3x | ˗ | y= 44.8 - 0.4x | y= 56.2 - 0.5x |
|  | p(ϐ) | ˗ | **<0.001** | **<0.001** | ˗ | **0.00** | **0.03** | ˗ | **<0.001** | **0.01** | ˗ | **<0.001** | **<0.001** |
| **Carotid tonometry (SCOR)** | r | 0.83 | ˗ | 0.66 | 0.75 | ˗ | 0.67 | 0.83 | ˗ | 0.57 | 0.83 | ˗ | 0.56 |
|  | p | **<0.001** | ˗ | **<0.001** | **<0.001** | ˗ | **<0.001** | **<0.001** | ˗ | **<0.001** | **<0.001** | ˗ | **<0.001** |
|  | Mean error (mmHg) | 10.46 | ˗ | 3.69 | 12.09 | ˗ | 8.54 | 10.37 | ˗ | 3.50 | 9.05 | ˗ | -0.91 |
|  | Mean error, CI 95% Upper Limit (mmHg) | 11.55 |  | 5.38 | 13.93 |  | 10.71 | 12.32 |  | 6.64 | 10.90 |  | 2.15 |
|  | Mean error, CI 95% Lower Limit (mmHg) | 9.37 |  | 2.00 | 10.24 | ˗ | 6.38 | 8.42 | ˗ | 0.36 | 7.20 | ˗ | -3.98 |
|  | p | **<0.001** | ˗ | **<0.001** | **<0.001** | ˗ | **<0.001** | **<0.001** | ˗ | **0.03** | **<0.001** | ˗ | 0.55 |
|  | Mean error, SD (mmHg) | 8.87 | ˗ | 13.77 | 8.35 | ˗ | 9.79 | 9.56 | ˗ | 15.34 | 8.40 | ˗ | 13.86 |
|  | Upper limit (mmHg) | -6.93 | ˗ | 30.68 | 28.45 | ˗ | 27.74 | 29.10 | ˗ | 33.56 | 25.52 | ˗ | 26.26 |
|  | Lower limit (mmHg) | 27.85 | ˗ | -23.31 | -4.28 | ˗ | -10.65 | -8.36 | ˗ | -26.56 | -7.42 | ˗ | -28.09 |
|  | Regression equation | y= -15.8 + 0.2x | ˗ | y= 15.5 - 0.1x | y= -20.5 - 0.3x | ˗ | y=-6.4 + 0.1x | y= -29.2 + 0.3x | ˗ | y= -4.5 + 0.06x | y= -44.8 + 0.4x | ˗ | y= 6.5 - 0.06x |
|  | p(ϐ) | **<0.001** | ˗ | 0.09 | **0.00** | ˗ | 0.19 | **<0.001** | ˗ | 0.56 | **<0.001** | ˗ | 0.63 |
| **Brachial oscillometry (MOG)** | r | 0.77 | 0.66 | ˗ | 0.78 | 0.67 | ˗ | 0.65 | 0.57 | ˗ | 0.60 | 0.56 | ˗ |
|  | p | **<0.001** | **<0.001** | ˗ | **<0.001** | **<0.001** | ˗ | **<0.001** | **<0.001** | ˗ | **<0.001** | **<0.001** | ˗ |
|  | Mean error (mmHg) | 6.60 | -3.69 | ˗ | 3.57 | -8.54 | ˗ | 6.55 | -3.50 | ˗ | 9.83 | 0.91 | ˗ |
|  | Mean error, CI 95% Upper Limit (mmHg) | 7.94 | -2.00 |  | 5.12 | -6.38 |  | 9.05 | -0.36 |  | 12.48 | 3.98 |  |
|  | Mean error, CI 95% Lower Limit (mmHg) | 5.25 | -5.38 | ˗ | 2.01 | -10.71 | ˗ | 4.05 | -6.64 | ˗ | 7.18 | -2.15 |  |
|  | p | **<0.001** | **<0.001** | ˗ | **<0.001** | **<0.001** | ˗ | **<0.001** | **0.03** | ˗ | **<0.001** | 0.55 | ˗ |
|  | Mean error, SD (mmHg) | 11.06 | 13.77 | ˗ | 7.12 | 9.79 | ˗ | 12.34 | 15.34 | ˗ | 12.05 | 13.86 | ˗ |
|  | Upper limit (mmHg) | -15.08 | -30.68 | ˗ | 17.52 | 10.65 | ˗ | 30.74 | 26.56 | ˗ | 33.45 | 28.09 | ˗ |
|  | Lower limit (mmHg) | 28.27 | 23.31 | ˗ | -10.39 | -27.74 | ˗ | -17.64 | -33.56 | ˗ | -13.79 | -26.26 | ˗ |
|  | Regression equation | y= -28.7 + 0.3x | y= -15.5 + 0.1x | ˗ | y= -14.8 + 0.2x | y= 6.4 - 0.1x | ˗ | y= -25.3 + 0.3x | y= 4.5 - 0.06x | ˗ | y= -56.2 + 0.5x | y= -6.5 + 0.06x | ˗ |
|  | p(ϐ) | **<0.001** | 0.09 | ˗ | **0.03** | 0.19 | ˗ | **0.01** | 0.56 | ˗ | **<0.001** | 0.63 | ˗ |
| RT: radial applanation tonometry record, obtained with SphygmoCor device (SCOR). CT: carotid applanation tonometry record, obtained with SCOR. BOSC: brachial oscillometry/plethysmography record, obtained with Mobil-O-Graph device (MOG). cSBP: central systolic blood pressure. r: correlation (Pearson) coefficient. β: slope of regression equation. Significance level: p value <0.05 (red text). Bland-Altman analysis: variable "x" was considered the mean of both methods compared (eg. (RT+CT)/2) and variable "y" the difference among first and second method (eg. RT minus CT). MBPosc: mean blood pressure measured by oscillometry. CI: confidence interval. | | | | | | | | | | | | | |
|  |  |  |  |  |  |  |  |  |  |  |  |  |  |
|  |  |  |  |  |  |  |  |  |  |  |  |  |  |
