# Supplementary material for: Aortic pressure and forward and backward wave components in children, adolescents and young-adults: Agreement between brachial oscillometry, radial and carotid tonometry data and analysis of factors associated with their differences
Source: PLoS One. 2019 Dec 19;14(12):e0226709. doi: 10.1371/journal.pone.0226709 (PMC6922407; doi:10.1371/journal.pone.0226709)
Supplement: S17 Table — (DOCX) [file pone.0226709.s035.docx]

| **S17 Table. cSBP: agreement among parameters measured with three different methods in the entire and age-related groups, calibrated with identical peripheral blood pressure levels obtained by oscillometry (Calibration scheme: pDBP/MBPosc) [Extended table]** | | | | | | | | | | | | | |
| --- | --- | --- | --- | --- | --- | --- | --- | --- | --- | --- | --- | --- | --- |
|  |  |  |  |  |  |  |  |  |  |  |  |  |  |
|  |  |  |  |  |  |  |  |  |  |  |  |  |  |
| **cPP** | | **Entire group [3-35 years]** | | | **Children [3-12 years]** | | | **Adolescents [12-18 years]** | | | **Young adults [18-35 years]** | | |
|  |  | **RT (SCOR)** | **CT (SCOR)** | **BOSC (MOG)** | **RT (SCOR)** | **CT (SCOR)** | **BOSC (MOG)** | **RT (SCOR)** | **CT (SCOR)** | **BOSC (MOG)** | **RT (SCOR)** | **CT (SCOR)** | **BOSC (MOG)** |
| **Radial tonometry (SCOR)** | r | ˗ | 0.80 | 0.80 | ˗ | 0.72 | 0.80 | ˗ | 0.81 | 0.73 | ˗ | 0.85 | 0.80 |
|  | p | ˗ | **<0.001** | **<0.001** | ˗ | **<0.001** | **<0.001** | ˗ | **<0.001** | **<0.001** | ˗ | **<0.001** | **<0.001** |
|  | Mean error (mmHg) | ˗ | -12.24 | -7.14 | ˗ | -14.26 | -4.44 | ˗ | -12.33 | -7.07 | ˗ | -10.21 | -10.01 |
|  | Mean error, CI 95% Upper Limit (mmHg) |  | -11.11 | -5.89 |  | -12.29 | -2.94 |  | -10.27 | -4.82 |  | -8.40 | -7.50 |
|  | Mean error, CI 95% Lower Limit (mmHg) |  | -13.37 | -8.38 |  | -16.23 | -5.94 |  | -14.38 | -9.33 |  | -12.02 | -12.52 |
|  | p | ˗ | **<0.001** | **<0.001** | ˗ | **<0.001** | **<0.001** | ˗ | **<0.001** | **<0.001** | ˗ | **<0.001** | **<0.001** |
|  | Mean error, SD (mmHg) | ˗ | 9.25 | 10.25 | ˗ | 8.92 | 6.83 | ˗ | 10.07 | 11.13 | ˗ | 8.20 | 11.42 |
|  | Upper limit (mmHg) | ˗ | 5.89 | 12.95 | ˗ | 3.22 | 8.95 | ˗ | 7.41 | 14.74 | ˗ | 5.87 | 12.38 |
|  | Lower limit (mmHg) | ˗ | -30.37 | -27.23 | ˗ | -31.74 | -17.83 | ˗ | -32.06 | -28.89 | ˗ | -26.29 | -32.40 |
|  | Regression equation | ˗ | y= 2.9 - 0.3x | y= 12.2 - 0.4x | ˗ | y= 2.4 - 0.3x | y= 4.4 - 0.2x | ˗ | y= 7.2 - 0.3x | y= 9.7 - 0.3x | ˗ | y= 7.6 - 0.3x | y= 19.6 - 0.5x |
|  | p(ϐ) | ˗ | **<0.001** | **<0.001** | ˗ | **<0.001** | **0.01** | ˗ | **<0.001** | **<0.001** | ˗ | **<0.001** | **<0.001** |
| **Carotid tonometry (SCOR)** | r | 0.80 | ˗ | 0.66 | 0.72 | ˗ | 0.69 | 0.81 | ˗ | 0.61 | 0.85 | ˗ | 0.70 |
|  | p | **<0.001** | ˗ | **<0.001** | **<0.001** | ˗ | **<0.001** | **<0.001** | ˗ | **<0.001** | **<0.001** | ˗ | **<0.001** |
|  | Mean error (mmHg) | 12.24 | ˗ | 5.00 | 14.26 | ˗ | 9.80 | 12.33 | ˗ | 5.11 | 10.21 | ˗ | 0.10 |
|  | Mean error, CI 95% Upper Limit (mmHg) | 13.37 |  | 6.65 | 16.23 |  | 11.94 | 14.38 |  | 2.06 | 12.02 |  | 3.09 |
|  | Mean error, CI 95% Lower Limit (mmHg) | 11.11 |  | 3.34 | 12.29 |  | 7.66 | 10.27 |  | 8.16 | 8.40 |  | -2.89 |
|  | p | **<0.001** | ˗ | **<0.001** | **<0.001** | ˗ | **<0.001** | **<0.001** | ˗ | **0.00** | **<0.001** | ˗ | 0.95 |
|  | Mean error, SD (mmHg) | 9.25 | ˗ | 13.44 | 8.92 | ˗ | 9.60 | 10.07 | ˗ | 14.89 | 8.20 | ˗ | 13.43 |
|  | Upper limit (mmHg) | 30.37 | ˗ | 31.35 | 31.74 | ˗ | 28.62 | 32.06 | ˗ | 34.28 | 26.29 | ˗ | 26.42 |
|  | Lower limit (mmHg) | -5.89 | ˗ | -21.36 | -3.22 | ˗ | -9.02 | -7.41 | ˗ | -24.07 | -5.87 | ˗ | -26.22 |
|  | Regression equation | y= -2.9 + 0.3x | ˗ | y= 11.4 - 0.1x | y= -2.4 + 0.3x | ˗ | y= 2.2 + 0.1x | y= -7.2 + 0.3x | ˗ | y= 2.6 + 0.04x | y= -7.6 + 0.3x | ˗ | y= 14.6 - 0.2x |
|  | p(ϐ) | **<0.001** | ˗ | 0.05 | **<0.001** | ˗ | 0.13 | **<0.001** | ˗ | 0.69 | **<0.001** | ˗ | **0.00** |
| **Brachial oscillometry (MOG)** | r | 0.80 | 0.66 | ˗ | 0.80 | 0.69 | ˗ | 0.73 | 0.61 | ˗ | 0.80 | 0.70 | ˗ |
|  | p | **<0.001** | **<0.001** | ˗ | **<0.001** | **<0.001** | ˗ | **<0.001** | **<0.001** | ˗ | **<0.001** | **<0.001** | ˗ |
|  | Mean error (mmHg) | 7.14 | -5.00 | ˗ | 4.44 | -9.80 | ˗ | 7.07 | -5.11 | ˗ | 10.01 | -0.10 | ˗ |
|  | Mean error, CI 95% Upper Limit (mmHg) | 8.38 | -3.34 |  | 5.94 | -7.66 |  | 9.33 | -2.06 |  | 12.52 | 2.89 |  |
|  | Mean error, CI 95% Lower Limit (mmHg) | 5.89 | -6.65 | ˗ | 2.94 | -11.94 | ˗ | 4.82 | -8.16 | ˗ | 7.50 | -3.09 |  |
|  | p | **<0.001** | **<0.001** | ˗ | **<0.001** | **<0.001** | ˗ | **<0.001** | **0.00** | ˗ | **<0.001** | 0.95 | ˗ |
|  | Mean error, SD (mmHg) | 10.25 | 13.44 | ˗ | 6.83 | 9.60 | ˗ | 11.13 | 14.89 | ˗ | 11.42 | 13.43 | ˗ |
|  | Upper limit (mmHg) | 27.23 | 21.36 | ˗ | 17.83 | 9.02 | ˗ | 28.89 | 24.07 | ˗ | 32.40 | 26.22 | ˗ |
|  | Lower limit (mmHg) | -12.95 | -31.35 | ˗ | -8.95 | -28.62 | ˗ | -14.74 | -34.28 | ˗ | -12.38 | -26.42 | ˗ |
|  | Regression equation | y= -12.2 + 0.4x | y= -11.4 + 0.1x | ˗ | y= -4.4 + 0.2x | y= -2.2 - 0.1x | ˗ | y= -9.7 + 0.3x | y= -2.6 - 0.04x | ˗ | y= -19.6 + 0.5x | y= -14.6 + 0.2x | ˗ |
|  | p(ϐ) | **<0.001** | 0.05 | ˗ | **0.01** | 0.13 | ˗ | **<0.001** | 0.69 | ˗ | **<0.001** | **0.00** | ˗ |
| RT: radial applanation tonometry record, obtained with SphygmoCor device (SCOR). CT: carotid applanation tonometry record, obtained with SCOR. BOSC: brachial oscillometry/plethysmography record, obtained with Mobil-O-Graph device (MOG). cPP: central pulse pressure. r: correlation (Pearson) coefficient. β: slope of regression equation. Significance level: p value <0.05 (red text). Bland-Altman analysis: variable "x" was considered the mean of both methods compared (eg. (RT+CT)/2) and variable "y" the difference among first and second method (eg. RT minus CT). MBPosc: mean blood pressure measured by oscillometry. CI: confidence interval. | | | | | | | | | | | | | |
|  |  |  |  |  |  |  |  |  |  |  |  |  |  |
|  |  |  |  |  |  |  |  |  |  |  |  |  |  |
