# Supplementary material for: Aortic pressure and forward and backward wave components in children, adolescents and young-adults: Agreement between brachial oscillometry, radial and carotid tonometry data and analysis of factors associated with their differences
Source: PLoS One. 2019 Dec 19;14(12):e0226709. doi: 10.1371/journal.pone.0226709 (PMC6922407; doi:10.1371/journal.pone.0226709)
Supplement: S18 Table — (DOCX) [file pone.0226709.s036.docx]

| **S18 Table. Pf: agreement among parameters measured with three different methods in the entire and age-related groups, calibrated with identical peripheral blood pressure levels obtained by oscillometry (Calibration scheme: pDBP/MBPosc) [Extended table]** | | | | | | | | | | | | | |
| --- | --- | --- | --- | --- | --- | --- | --- | --- | --- | --- | --- | --- | --- |
|  |  |  |  |  |  |  |  |  |  |  |  |  |  |
|  |  |  |  |  |  |  |  |  |  |  |  |  |  |
| **Pf** | | **Entire group [3-35 years]** | | | **Children [3-12 years]** | | | **Adolescents [12-18 years]** | | | **Young adults [18-35 years]** | | |
|  |  | **RT (SCOR)** | **CT (SCOR)** | **BOSC (MOG)** | **RT (SCOR)** | **CT (SCOR)** | **BOSC (MOG)** | **RT (SCOR)** | **CT (SCOR)** | **BOSC (MOG)** | **RT (SCOR)** | **CT (SCOR)** | **BOSC (MOG)** |
| **Radial tonometry (SCOR)** | r | ˗ | 0.75 | 0.76 | ˗ | 0.66 | 0.75 | ˗ | 0.79 | 0.69 | ˗ | 0.73 | 0.77 |
|  | p | ˗ | **<0.001** | **<0.001** | ˗ | **<0.001** | **<0.001** | ˗ | **<0.001** | **<0.001** | ˗ | **<0.001** | **<0.001** |
|  | Mean error (mmHg) | ˗ | -11.35 | 8.89 | ˗ | -13.59 | 8.47 | ˗ | -11.20 | 9.38 | ˗ | -9.85 | 8.77 |
|  | Mean error, CI 95% Upper Limit (mmHg) |  | -9.95 | 9.90 |  | -11.24 | 9.87 | ˗ | -8.79 | 11.41 | ˗ | -7.33 | 10.58 |
|  | Mean error, CI 95% Lower Limit (mmHg) |  | -12.76 | 7.87 |  | -15.94 | 7.07 |  | -13.61 | 7.36 |  | -12.38 | 6.96 |
|  | p | ˗ | **<0.001** | **<0.001** | ˗ | **<0.001** | **<0.001** | ˗ | **<0.001** | **<0.001** | ˗ | **<0.001** | **<0.001** |
|  | Mean error, SD (mmHg) | ˗ | 9.11 | 8.01 | ˗ | 7.73 | 6.30 | ˗ | 9.65 | 9.67 | ˗ | 9.33 | 7.57 |
|  | Upper limit (mmHg) | ˗ | 6.50 | 24.59 | ˗ | 1.55 | 20.82 | ˗ | 7.71 | 28.33 | ˗ | 8.43 | 23.61 |
|  | Lower limit (mmHg) | ˗ | -29.20 | -6.82 | ˗ | -28.73 | -3.88 | ˗ | -30.11 | -9.56 | ˗ | -28.14 | -6.07 |
|  | Regression equation | ˗ | y= -0.5 -0.2x | y= 2.3 + 0.2x | ˗ | y= -1.7 - 0.3x | y= 0.3 + 0.2x | ˗ | y= 4.9 - 0.3x | y= -0.5 + 0.2x | ˗ | y= -1.3 - 0.2x | y= 5.8 + 0.07x |
|  | p(ϐ) | ˗ | **<0.001** | **<0.001** | ˗ | 0.07 | **0.01** | ˗ | **0.00** | **0.01** | ˗ | 0.13 | 0.07 |
| **Carotid tonometry (SCOR)** | r | 0.75 | ˗ | 0.62 | 0.66 | ˗ | 0.46 | 0.79 | ˗ | 0.68 | 0.73 | ˗ | 0.59 |
|  | p | **<0.001** | ˗ | **<0.001** | **<0.001** | ˗ | **<0.001** | **<0.001** | ˗ | **<0.001** | **<0.001** | ˗ | **<0.001** |
|  | Mean error (mmHg) | 11.35 | ˗ | 19.70 | 13.59 | ˗ | 22.04 | 11.20 | ˗ | 19.67 | 9.85 | ˗ | 18.21 |
|  | Mean error, CI 95% Upper Limit (mmHg) | 12.76 |  | 21.31 | 15.94 |  | 24.69 | 13.61 |  | 22.38 | 12.38 |  | 21.15 |
|  | Mean error, CI 95% Lower Limit (mmHg) | 9.95 |  | 18.08 | 11.24 |  | 19.39 | 8.79 |  | 16.96 | 7.33 |  | 15.27 |
|  | p | **<0.001** | ˗ | **<0.001** | **<0.001** | ˗ | **<0.001** | **<0.001** | ˗ | **<0.001** | **<0.001** | ˗ | **<0.001** |
|  | Mean error, SD (mmHg) | 9.11 | ˗ | 10.77 | 7.73 | ˗ | 8.73 | 9.65 | ˗ | 11.19 | 9.33 | ˗ | 11.48 |
|  | Upper limit (mmHg) | 29.20 | ˗ | 40.81 | 28.73 | ˗ | 39.15 | 30.11 | ˗ | 41.60 | 28.14 | ˗ | 40.71 |
|  | Lower limit (mmHg) | -6.50 | ˗ | -1.41 | -1.55 | ˗ | 4.93 | -7.71 | ˗ | -2.26 | -8.43 | ˗ | -4.29 |
|  | Regression equation | y= 0.5 + 0.2x | ˗ | y= 3.3 + 0.4x | y= 2.5 + 0.3x | ˗ | y= -6.1 + 0.7x | y= -4.9 + 0.3x | ˗ | y= -2.5 + 0.5x | y= 1.3 + 0.2x | ˗ | y= 6.3 + 0.2x |
|  | p(ϐ) | **<0.001** | ˗ | **<0.001** | 0.07 | ˗ | **<0.001** | **0.00** | ˗ | **<0.001** | 0.13 | ˗ | 0.06 |
| **Brachial oscillometry (MOG)** | r | 0.76 | 0.62 | ˗ | 0.75 | 0.46 | ˗ | 0.69 | 0.68 | ˗ | 0.77 | 0.59 | ˗ |
|  | p | **<0.001** | **<0.001** | ˗ | **<0.001** | **<0.001** | ˗ | **<0.001** | **<0.001** | ˗ | **<0.001** | **<0.001** | ˗ |
|  | Mean error (mmHg) | -8.89 | -19.70 | ˗ | -8.47 | -22.04 | ˗ | -9.38 | -19.67 | ˗ | -8.77 | -18.21 | ˗ |
|  | Mean error, CI 95% Upper Limit (mmHg) | -7.87 | -18.08 |  | -7.07 | -19.39 |  | -7.36 | -16.96 |  | -6.96 | -15.27 |  |
|  | Mean error, CI 95% Lower Limit (mmHg) | -9.90 | -21.31 |  | -9.87 | -24.69 | ˗ | -11.41 | -22.38 | ˗ | -10.58 | -21.15 |  |
|  | p | **<0.001** | **<0.001** | ˗ | **<0.001** | **<0.001** | ˗ | **<0.001** | **<0.001** | ˗ | **<0.001** | **<0.001** | ˗ |
|  | Mean error, SD (mmHg) | 8.01 | 10.77 | ˗ | 6.30 | 8.73 | ˗ | 9.67 | 11.19 | ˗ | 7.57 | 11.48 | ˗ |
|  | Upper limit (mmHg) | 6.82 | 1.41 | ˗ | 3.88 | -4.93 | ˗ | 9.56 | 2.26 | ˗ | 6.07 | 4.29 | ˗ |
|  | Lower limit (mmHg) | -24.59 | -40.81 | ˗ | -20.82 | -39.15 | ˗ | -28.33 | -41.60 | ˗ | -23.61 | -40.71 | ˗ |
|  | Regression equation | y= -2.3 - 0.2x | y= -3.3 - 0.4x | ˗ | y= -0.3 - 0.2x | y= 6.1 - 0.7x | ˗ | y= 0.5 - 0.2x | y= 2.5 - 0.5x | ˗ | y= -5.8 - 0.07x | y= -6.3 - 0.2x | ˗ |
|  | p(ϐ) | **<0.001** | **<0.001** | ˗ | **0.01** | **<0.001** | ˗ | **0.01** | **<0.001** | ˗ | 0.07 | 0.06 | ˗ |
| RT: radial applanation tonometry record, obtained with SphygmoCor device (SCOR). CT: carotid applanation tonometry record, obtained with SCOR. BOSC: brachial oscillometry/plethysmography record, obtained with Mobil-O-Graph device (MOG). Pb: backward wave height (amplitude). r: correlation (Pearson) coefficient. β: slope of regression equation. Significance level: p value <0.05 (red text). Bland-Altman analysis: variable "x" was considered the mean of both methods compared (eg. (RT+CT)/2) and variable "y" the difference among first and second method (eg. RT minus CT). MBPosc: mean blood pressure measured by oscillometry. CI: confidence interval. | | | | | | | | | | | | | |
|  |  |  |  |  |  |  |  |  |  |  |  |  |  |
|  |  |  |  |  |  |  |  |  |  |  |  |  |  |
|  |  |  |  |  |  |  |  |  |  |  |  |  |  |
