# Supplementary material for: Aortic pressure and forward and backward wave components in children, adolescents and young-adults: Agreement between brachial oscillometry, radial and carotid tonometry data and analysis of factors associated with their differences
Source: PLoS One. 2019 Dec 19;14(12):e0226709. doi: 10.1371/journal.pone.0226709 (PMC6922407; doi:10.1371/journal.pone.0226709)
Supplement: S19 Table — (DOCX) [file pone.0226709.s037.docx]

| **S19 Table. Pb: agreement among parameters measured with three different methods in the entire and age-related groups, calibrated with identical peripheral blood pressure levels obtained by oscillometry (Calibration scheme: pDBP/MBPosc) [Extended table]** | | | | | | | | | | | | | |
| --- | --- | --- | --- | --- | --- | --- | --- | --- | --- | --- | --- | --- | --- |
|  |  |  |  |  |  |  |  |  |  |  |  |  |  |
|  |  |  |  |  |  |  |  |  |  |  |  |  |  |
| **Pb** | | **Entire group [3-35 years]** | | | **Children [3-12 years]** | | | **Adolescents [12-18 years]** | | | **Young adults [18-35 years]** | | |
|  |  | **RT (SCOR)** | **CT (SCOR)** | **BOSC (MOG)** | **RT (SCOR)** | **CT (SCOR)** | **BOSC (MOG)** | **RT (SCOR)** | **CT (SCOR)** | **BOSC (MOG)** | **RT (SCOR)** | **CT (SCOR)** | **BOSC (MOG)** |
| **Radial tonometry (SCOR)** | r | ˗ | 0.83 | 0.61 | ˗ | 0.63 | 0.74 | ˗ | 0.80 | 0.62 | ˗ | 0.86 | 0.55 |
|  | p | ˗ | **<0.001** | **<0.001** | ˗ | **<0.001** | **<0.001** | ˗ | **<0.001** | **<0.001** | ˗ | **<0.001** | **<0.001** |
|  | Mean error (mmHg) | ˗ | -2.50 | -2.97 | ˗ | -0.84 | -0.54 | ˗ | -2.75 | -3.73 | ˗ | -3.58 | -4.95 |
|  | Mean error, CI 95% Upper Limit (mmHg) |  | -2.04 | -2.21 |  | 0.06 | 0.20 | ˗ | -2.04 | -2.39 | ˗ | -2.83 | -3.32 |
|  | Mean error, CI 95% Lower Limit (mmHg) |  | -2.97 | -3.73 |  | -1.75 | -1.29 |  | -3.46 | -5.08 |  | -4.33 | -6.58 |
|  | p | ˗ | **<0.001** | **<0.001** | ˗ | 0.07 | 0.15 | ˗ | **<0.001** | **<0.001** | ˗ | **<0.001** | **<0.001** |
|  | Mean error, SD (mmHg) | ˗ | 3.04 | 6.00 | ˗ | 3.01 | 3.34 | ˗ | 2.85 | 6.42 | ˗ | 2.77 | 6.85 |
|  | Upper limit (mmHg) | ˗ | 3.45 | 8.79 | ˗ | 5.05 | 6.01 | ˗ | 2.83 | 8.86 | ˗ | 1.85 | 8.48 |
|  | Lower limit (mmHg) | ˗ | -8.46 | -14.73 | ˗ | -6.74 | -7.10 | ˗ | -8.33 | -16.33 | ˗ | -9.02 | -18.38 |
|  | Regression equation | ˗ | y= 1.8 - 0.2x | y= 10.9 - 0.7x | ˗ | y= -0.4 - 0.02x | y= 2.6 - 0.2x | ˗ | y= -0.8 - 0.1x | y= 11.4 - 0.7x | ˗ | y= 2.3- 0.3x | y= 14.5 - 0.9x |
|  | p(ϐ) | ˗ | **<0.001** | **<0.001** | ˗ | 0.87 | **0.04** | ˗ | 0.24 | **<0.001** | ˗ | **0.00** | **<0.001** |
| **Carotid tonometry (SCOR)** | r | 0.83 | ˗ | 0.60 | 0.63 | ˗ | 0.51 | 0.80 | ˗ | 0.64 | 0.86 | ˗ | 0.57 |
|  | p | **<0.001** | ˗ | **<0.001** | **<0.001** | ˗ | **<0.001** | **<0.001** | ˗ | **<0.001** | **<0.001** | ˗ | **<0.001** |
|  | Mean error (mmHg) | 2.50 | ˗ | -0.95 | 0.84 | ˗ | 0.08 | 2.75 | ˗ | -1.40 | 3.58 | ˗ | -1.32 |
|  | Mean error, CI 95% Upper Limit (mmHg) | 2.97 |  | -0.08 | 1.75 |  | 1.27 | 3.46 |  | 0.09 | 4.33 |  | 0.34 |
|  | Mean error, CI 95% Lower Limit (mmHg) | 2.04 |  | -1.81 | -0.06 |  | -1.10 | 2.04 |  | -2.89 | 2.83 |  | -2.98 |
|  | p | **<0.001** | ˗ | **0.03** | 0.07 | ˗ | 0.89 | **<0.001** | ˗ | 0.07 | **<0.001** | ˗ | 0.12 |
|  | Mean error, SD (mmHg) | 3.04 | ˗ | 5.81 | 3.01 | ˗ | 3.93 | 2.85 | ˗ | 6.17 | 2.77 | ˗ | 6.48 |
|  | Upper limit (mmHg) | -3.45 | ˗ | 10.45 | 6.74 | ˗ | 7.79 | 8.33 | ˗ | 10.68 | 9.02 | ˗ | 11.38 |
|  | Lower limit (mmHg) | 8.46 | ˗ | -12.34 | -5.05 | ˗ | -7.62 | -2.83 | ˗ | -13.49 | -1.85 | ˗ | -14.03 |
|  | Regression equation | y= -1.8 + 0.2x | ˗ | y= 10.9 - 0.5x | y= 0.4 + 0.02x | ˗ | y= 5.1 -0.3x | y= 0.8 + 0.1x | ˗ | y= 12.8 - 0.7x | y= -2.3 +0.3x | ˗ | y= 12.3 - 0.6x |
|  | p(ϐ) | **<0.001** | ˗ | **<0.001** | 0.87 | ˗ | 0.12 | 0.24 | ˗ | **<0.001** | **0.00** | ˗ | **<0.001** |
| **Brachial oscillometry (MOG)** | r | 0.61 | 0.60 | ˗ | 0.74 | 0.51 | ˗ | 0.62 | 0.64 | ˗ | 0.55 | 0.57 | ˗ |
|  | p | **<0.001** | **<0.001** | ˗ | **<0.001** | **<0.001** | ˗ | **<0.001** | **<0.001** | ˗ | **<0.001** | **<0.001** | ˗ |
|  | Mean error (mmHg) | 2.97 | 0.95 | ˗ | 0.54 | -0.08 | ˗ | 3.73 | 1.40 | ˗ | 4.95 | 1.32 | ˗ |
|  | Mean error, CI 95% Upper Limit (mmHg) | 3.73 | 1.81 |  | 1.29 | 1.10 |  | 5.08 | 2.89 |  | 6.58 | 2.98 |  |
|  | Mean error, CI 95% Lower Limit (mmHg) | 2.21 | 0.08 |  | -0.20 | -1.27 |  | 2.39 | -0.09 |  | 3.32 | -0.34 |  |
|  | p | **<0.001** | **0.03** | ˗ | 0.15 | 0.89 | ˗ | **<0.001** | 0.07 | ˗ | **<0.001** | 0.12 | ˗ |
|  | Mean error, SD (mmHg) | 6.00 | 5.81 | ˗ | -3.34 | -3.93 | ˗ | -6.42 | -6.17 | ˗ | -6.85 | -6.48 | ˗ |
|  | Upper limit (mmHg) | -8.79 | -10.45 | ˗ | 7.10 | 7.62 | ˗ | 16.33 | 13.49 | ˗ | 18.38 | 14.03 | ˗ |
|  | Lower limit (mmHg) | 14.73 | 12.34 | ˗ | -6.01 | -7.79 | ˗ | -8.86 | -10.68 | ˗ | -8.48 | -11.38 | ˗ |
|  | Regression equation | y= -10.9 + 0.7x | y= -10.9 + 0.5x | ˗ | y= -2.6 + 0.2x | y= -5.1 + 0.3x | ˗ | y= -11.4 + 0.7x | y= -12.8 + 0.7x | ˗ | y= -14.5 + 0.9x | y= -12.3 + 0.6x | ˗ |
|  | p(ϐ) | **<0.001** | **<0.001** | ˗ | **0.04** | 0.12 | ˗ | **<0.001** | **<0.001** | ˗ | **<0.001** | **<0.001** | ˗ |
| RT: radial applanation tonometry record, obtained with SphygmoCor device (SCOR). CT: carotid applanation tonometry record, obtained with SCOR. BOSC: brachial oscillometry/plethysmography record, obtained with Mobil-O-Graph device (MOG). Pb: backward wave height (amplitude). r: correlation (Pearson) coefficient. β: slope of regression equation. Significance level: p value <0.05 (red text). Bland-Altman analysis: variable "x" was considered the mean of both methods compared (eg. (RT+CT)/2) and variable "y" the difference among first and second method (eg. RT minus CT). MBPosc: mean blood pressure measured by oscillometry. CI: confidence interval. | | | | | | | | | | | | | |
|  |  |  |  |  |  |  |  |  |  |  |  |  |  |
|  |  |  |  |  |  |  |  |  |  |  |  |  |  |
|  |  |  |  |  |  |  |  |  |  |  |  |  |  |
