# Supplementary material for: Aortic pressure and forward and backward wave components in children, adolescents and young-adults: Agreement between brachial oscillometry, radial and carotid tonometry data and analysis of factors associated with their differences
Source: PLoS One. 2019 Dec 19;14(12):e0226709. doi: 10.1371/journal.pone.0226709 (PMC6922407; doi:10.1371/journal.pone.0226709)
Supplement: S20 Table — (DOCX) [file pone.0226709.s038.docx]

| **S20 Table. Agreement among oscillometry-derived and calculated mean blood pressure (MBPosc and MBPc, respectively) obtained with Mobil-O-Graph device (MOG).** | | |
| --- | --- | --- |
|  |  |  |
|  | | **MBPosc - MBPc** |
| **Brachial oscillometry (MOG)** | r | 0.9887 |
|  | p | **<0.0001** |
|  | Mean error (mmHg) | 6.9211 |
|  | Mean error, CI 95% Upper Limit (mmHg) | 7.08 |
|  | Mean error, CI 95% Lower Limit (mmHg) | 6.7621 |
|  | P (Mean error) | **<0.0001** |
|  | Mean error, SD (mmHg) | 1.3166 |
|  | Upper limit (mmHg) | 9.5015 |
|  | Lower limit (mmHg) | 4.3406 |
|  | Regression equation | y= 4.03 + 0.03x |
|  | p (β) | **0.0003** |

r: correlation (Pearson) coefficient. β: slope of regression equation. Significance level: p value <0.05 (red text). Bland-Altman analysis: variable "x" was the mean of the compared methods ((MBPosc + MBPc)/2) and variable "y" the difference between the first and second method (MBPosc - MBPc). MBPc: mean blood pressure calculated as pDBP+((pSBP-pDBP)/3). CI: confidence interval.
