# Supplementary material for: Aortic pressure and forward and backward wave components in children, adolescents and young-adults: Agreement between brachial oscillometry, radial and carotid tonometry data and analysis of factors associated with their differences
Source: PLoS One. 2019 Dec 19;14(12):e0226709. doi: 10.1371/journal.pone.0226709 (PMC6922407; doi:10.1371/journal.pone.0226709)
Supplement: Attachment — Submitted filename: Response to reviewers.docx [file pone.0226709.s039.docx]

**06 -Nov-2019**

**Dear, Giacomo Pucci**

**Academic Editor**

**PLOS ONE**

I am sending the revised version of the original manuscript (PONE-D-19-22199): ***"Aortic pressure and forward and backward wave components in children, adolescents and young-adults: agreement between brachial oscillometry, radial and carotid tonometry data and analysis of factors associated with their differences"***, to be considered for publication as full length article in Plos One.

All reviewers’ suggestions were considered in the new version ('Response to Reviewers'; below). We thank the interest in our manuscript, and the reviewers’ comments that allowed us to increase the clarity of the manuscript.

Sincerely,

**Prof. Dr. Daniel Bia**

Physiology Department, School of Medicine.

Universidad de la República, Montevideo, Uruguay

Phone: 5982-9243414 (3313); e-mail: dbia@fmed.edu.uy

---------------------------------------------------------------------------------------------------------------------------------

**Academic Editor:** Please ensure that your manuscript meets PLOS ONE's style requirements, including those for file naming.

**Authors:** We believe that the manuscript meets all the requirements. Thanks for the comments and suggestions that have contributed to improve the manuscript.

**Academic Editor:** In your Methods section, please provide additional information about the participant recruitment method and the demographic details of your participants. Please ensure you have provided sufficient details to replicate the analyses such as: a) the recruitment date range (month and year), b) a description of any inclusion/exclusion criteria that were applied to participant recruitment, c) a statement as to whether your sample can be considered representative of a larger population, d) a description of how participants were recruited, and e) descriptions of where participants were recruited and where the research took place. Moreover, please specify how the levels of physical activity were assessed.

**Authors:** As requested, in the revised version we provided additional methodological information.

-------------------------------------------------------------------------------------------------------------------------------

**Reviewer #1:** This is an interesting study on the possible differences between different methods of assessing central blood pressure levels in children, adolescents and young adults. Τhe authors aimed to analyze, in children, adolescents and young-adults (1) the agreement between cSBP, cPP, Pf and Pb obtained using carotid (CT) and radial tonometry (RT) and brachial-oscillometry (BOSC); and identify (2) explanatory factors for the differences between the approaches-data. 1685 subjects (mean/range age: 14/3-35 y.o.) assigned to three age-related groups (3-12; 12-18; 18-35 y.o.) were included. cSBP, cPP, Pf and Pb were assessed with BOSC (Mobil-O-Graph), CT and RT (SphygmoCor) records. Two calibration schemes were considered: MBPc and MBPosc for calibrations to similar BP levels. Correlation, Bland-Altman tests and multiple regression models were applied. Systematic and proportional errors were observed; errors´ statistical significance and values varied depending on the parameter analyzed, methods compared and group considered. The explanatory factors for the differences between data obtained from the different approaches varied depending on the methods compared. The highest cSBP and cPP were obtained from CT; the lowest from RT. The study is well-performed. There are several issues that if addressed could improve the manuscript.

**Authors:** Thanks for your revision and comments.

**Reviewer #1:** The length of the manuscript must be significantly reduced and especially the Discussion.

**Authors:** Since we were asked (1) by the Editor to include more methodological information and (2) by Reviewer 2 to perform new analyses (which we did) it was difficult to reduce the length of the article.

However, as was requested, and without reducing the quality of the information provided, a significant part of the new analyses and the requested information (e.g. description of the wave separation analysis - WSA -) was included as Supplementary Material.

**Reviewer #1:** Please avoid having the figure legends and tables between the text because it gets really confusing trying to read through the manuscript. Please have a separate part with Tables and Figure legends. Please place all the supplemental material in a different to the main manuscript file.

**Authors:** We do understand the reviewer concern. However, that is what is indicated in the "Instructions for authors" (see below). We could have changed it, but the Editor has asked us to: ***"Please ensure that your manuscript meets PLOS ONE's style requirements****, including .... "*.

Supplementary Tables and Figures are in separate files.

The only thing we have included at the end of the manuscript file (taking into account the "Instructions for authors") was the description of the supplementary files ("Supporting information captions").

**Reviewer #1:** In Table 1, for clarity and space reasons please remove the min and max columns as well as the interquartile range unless it is necessary due to a non-normal distribution. Moreover, include in the same column the mean and SD values. If this is done please present the overall and the other 3 smaller populations in a single table. In Table 2, please include the mean and SD values in the same column. Please report the decimal values in the values in Table 2, because the p-values are different for similar differences and thus this means there is information missing. Moreover, when the value of p is ≥0.01, please report only 2 decimals. Table 3 needs to be reduced in size as well. I would keep only the r and p-values of the correlations and have the rest of the data in a supplemental table.

**Authors:** As requested, in Table 1 we eliminated the minimum, maximum as well as the 25th and 75th percentile values. In addition, data was unified in a single Table.

As suggested, Table 2 was modified (decimal values were added).

Table 3 was significantly shortened (its full version was included as a Supplementary Table). In the new (shortened) Table 3 we chose to keep the value of R and p of the correlations, and fundamental information of Bland-Altman tests, which allowed the existence of systematic (mean error, ME) and/or proportional errors to be assessed. In other words, "ME, C.I. 95% U.L. (mmHg)", "ME, C.I. 95% L.L. (mmHg)", "C.I. 95%, Upper limit (mmHg)" and "C.I. 95%, Lower limit (mmHg)" data (a total or 32 rows) were eliminated.

We kept mean and SD values in different columns due to we considered this enhances the visual clarity of the Table (mainly in the more extensive Tables - e.g. Table 2 -).

**Reviewer #1:** The authors reports errors as follows: “~2 to ~9”. The exact values should be included throughout the manuscript instead of using the ~ sign.

**Authors:** The requested change was done.

**Reviewer #1:** “Male sex showed the greatest differences between methods.” How can the authors explain this result? “Differences in cSBP between RT and BOSC were explained by the BH of the subject.” How do the authors explain this result? Could be the fact that BOSC uses an algorithm that incorporates height for the assessment of cBPs?

**Authors:** The issue was analyzed in greater detail in the revised version. Furthermore, the (additional) data analysis done in response to Reviewer #2 comments also contributed to explain the finding.

**Reviewer #1:** The authors should report also their intra- and inter-observer reproducibility, because there is a chance that these errors in assessment between techniques are due to large variability in the assessment.

**Authors:** The requested information was included summarily in the revised version. Detailed data was included in the Supplementary Materials´ section.

**Reviewer #1:** In the study population criteria please change “cardioactive” to “vasoactive”. Please correct “have shown to met” to “have shown to meet”. Please correct “("intantaneous blood pressure")” to “("instantaneous blood pressure")”. Please correct “Pf y Pb” to “Pf and Pb”. Please change “were observed when using TR.” to “were observed when using RT.”. Please change “by Sharma et al.” to “by Sharman et al.”.

**Authors:** The text was revised. Mistakes were corrected. Thanks.

**Reviewer #1:** Please improve the resolution of all images because the cannot be properly assessed in their current format.

**Authors:** As suggested, all figures were evaluated and corrected using the recommended system (*Preflight Analysis and Conversion Engine (PACE) digital diagnostic tool, https://pacev2.apexcovantage.com/.)*

**Reviewer #1:** 6. “Fig 4 (data included in S6 Table)”. Please report these data either on text or figure but not both.

**Authors:** We believe that both are useful, since Figure 4 is in the article (main text), highlighting most relevant haemodynamic information, while "Table S6" is a Supplementary Table included in Annexes (there, interested authors can find detailed numerical information). That is, Figure 4 does not show all the information included in Table S6. On the other hand, the inclusion of Table S6 in the manuscript (main text) would not make sense as the information contained is extensive and could be considered excessive in that context.

**Reviewer #1:** Please report in the supplement the exact step by step method you used to conduct the wave separation analysis based on the observed aortic waveforms in each device and which given parameters you used from each device.

**Authors:** As requested, a special document was included as Supplementary Material explaining the exact, step by step, method used to carry out wave separation analysis based on recorded carotid (SphygmoCor) and mathematically-derived aortic (SphygmoCor and Mobil-O-Graph) waveforms. We included examples of the analysis of records obtained in a real subject, using both softwares.

**Reviewer #1:** “When analyzing the age impact, the magnitude of the associations and differences varied depending on the approaches and calibration methods considered. For example, an increase in age equal to 10 years could be associated with a reduction in the differences in Pf equal to ~2 mmHg, ~4 mmHg, or ~6 mmHg, depending on whether RT-BOSC (cal. pDBP/MBPc), CT-BOSC (cal. pDBP/MBPc) or CT-BOSC (cal. pDBP/MBPosc) are compared.” “A BH increase equal to 1 meter during growth would associate an increase in Pb differences equal to: ~2 mmHg for RT-CT (calibration: pDBP/MBPosc); ~7 mmHg and ~11 mmHg for RT-BOSC (calibration: pDBP/MBPc and pDBP/MBPosc, respectively), and ~5 and ~7 mmHg for CT-BOSC (calibration: pDBP/MBPc and pDBP/MBPosc, respectively).” These parts seem more like a discussion rather than a result. If the authors want to keep it, they could move it in the Discussion section.

**Authors:** As suggested, the text was modified and included in the Discussion section.

**Reviewer #1:** The authors in the beginning of their Discussion report more than 5 different results. They must choose which ones of them are the most essential and report only them, because it get really confusing with so many results in such a short space.

**Authors:** As was requested, without reducing the quality of the information provided, the initial paragraph was modified (simplified) in order to clarify the message given.

-----------------------------------------------------------------------------------------------------------------------------------

**Reviewer #2:** This is an interesting study exploring agreement between different methods (carotid and radial tonometry and brachial oscillometry) in measuring central blood pressure and pulse wave analysis parameters in a large cohort of children, adolescents and young-adults. The study is well conducted. The results highlighting the highest central BP levels obtained from carotid tonometry and the lowest by using radial tonometry are helpful when interpreting and comparing results of different studies.

**Authors:** Thanks for the comments and suggestions that have contributed to improve the article.

**Reviewer #2:** I have two requests for the authors, which require further analysis which may improve the quality of the paper. 1) It seems that the way of calculating mean BP, necessary for calibration of all methods, may significantly change the estimation of central BP parameters. Is it possible to clarify which variables may influence the difference between MBPc and MBPosc, using a multiple regression model?

**Authors:** The requested analysis was performed in three steps. First, Bland-Altman analyses were performed to evaluate the agreement between MBPosc and MBPc (Supplementary Materials). Second, we identified demographic, anthropometric, cardiovascular risk factors and/or haemodynamic characteristics associated with the differences between MBPosc and MBPc (simple and point bi-serial correlation analysis) (new Table 5). Third, we included the selected independent variables (those with a p˂0.1) in multiple linear regression models (MLR) (new Table 6).

**Reviewer #2:** When interpreting the variables explaining absolute differences between methods (Table 4), a possibility is that actual BP levels (and particularly DBP) and heart rate may have a significant influence. My suggestion is to repeat the analysis in Table 4 by introducing these two parameters in the multiple regression models.

**Authors:** The suggestion was accepted. Thank you for your contribution. The requested analysis was performed and results were included in the *new (modified)* Table 4.
