# Supplementary material for: Aortic pressure and forward and backward wave components in children, adolescents and young-adults: Agreement between brachial oscillometry, radial and carotid tonometry data and analysis of factors associated with their differences
Source: PLoS One. 2019 Dec 19;14(12):e0226709. doi: 10.1371/journal.pone.0226709 (PMC6922407; doi:10.1371/journal.pone.0226709)
Supplement: Attachment — Submitted filename: Response to reviewers.docx [file pone.0226709.s040.docx]

**29 -Nov-2019**

**Dear, Giacomo Pucci**

**Academic Editor**

**PLOS ONE**

I am sending the second revised version of the original manuscript (PONE-D-19-22199): ***"Aortic pressure and forward and backward wave components in children, adolescents and young-adults: agreement between brachial oscillometry, radial and carotid tonometry data and analysis of factors associated with their differences"***, to be considered for publication as full length article in Plos One.

All reviewers’ suggestions were considered in the new version ('Response to Reviewers'; below). We thank the interest in our manuscript, and the reviewers’ comments that allowed us to increase the clarity of the manuscript.

Sincerely,

**Prof. Dr. Daniel Bia**

Physiology Department, School of Medicine.

Universidad de la República, Montevideo, Uruguay

Phone: 5982-9243414 (3313); e-mail: dbia@fmed.edu.uy

---------------------------------------------------------------------------------------------------------------------------------

**Reviewer #1:** The authors have adequately answered most of the Editor’s and Reviewers’ comments. This paper has been improved. However, there are some small issues that still need their attention.

**Authors:** Thanks for the comments and suggestions that contributed to improve the manuscript.

**Reviewer #1:** 1. It is weird that the resolution in the Supplem. Figures is far better than the one in the main Figures. The labels are too blur and not visible for most of the Figures of the main manuscript.

**Authors:** As requested, in the revised version (1) Figures were improved (e.g. font size and graphics symbols were increased and the legends became more visible).

**Reviewer #1:** 2. The manuscript as commented in my first review is extremely long (reaching close to 10,000 words) and thus not easily readable. I was hoping that the authors would be able to shorten it after the revision, but despite their great efforts the length is not fit for publication. In fact, the number of supplemental tables (20) and figures (17) is huge. Despite, the good scientific background of the study the authors have not managed to report their results in a presentable manner. I think the authors should try and shorten the manuscript or if they think this is not feasible try to resubmit their findings in more than 1 articles. One example of ways to reduce the size is the analysis presented in Table 6. There is no reason to use both enter and stepwise analysis in the models. Only the stepwise model would be sufficient.

**Authors:** We understand the position of the Reviewer. In addition, we understand that while Plos One does not limit words, authors must be concise (*). In this context, we reduced the length of the article (approximately in 3,000 words). **Then we consider it currently has a number of words that we believe is not excessive (Main text: Introduction + Methods + Results + Discussion + Conclusion: 5,136 words). See below the word count details.**

**It should be noted that we were asked for additional information and new (complementary) data analysis in the first revision of the original text. Consequently, the effort to shorten the work has been important.**

We consider ed the example given by the reviewer, but eliminating ENTER method from Table 6 would only account for a reduction equal to 25-30 words. Taking into account this and that eliminating the analysis would reduce the quality of the manuscript and remove data already approved by Reviewer 2, we did not modify Table 6.

** Ploos One Instructions for authors: "Length: Manuscripts can be any length. There are no restrictions on word count, number of figures, or amount of supporting information.We encourage you to present and discuss your findings concisely."*

|  | **Word count** | |
| --- | --- | --- |
| **Section** | **Previous revised version** | **New (Actual) revised version** |
| Abstract | 250 | 250 |
| Introduction | 491 | 487 |
| Material and Methods (*) | 1561 | 1493 |
| Results (*) | 1266 | 1181 |
| Discussion | 1907 | 1783 |
| Conclusion | 195 | 192 |
| **SUBTOTAL (Introduction - Conclusion)** | **5420** | **5136** |
| Acknowledgments | 17 | 17 |
| References | 1214 (37 references) | 1188 (37 references) |
| Supporting information | 3512 | 805 |
| **TOTAL WORD FILE** | **10413** | **7396** |

* without considering tables and legend of figures
